# Functional Landscape of PCGF Proteins Reveals Both RING1A/B-Dependent-and RING1A/B-Independent-Specific Activities

**DOI:** 10.1016/j.molcel.2019.04.002

**Published:** 2019-06-06

**Authors:** Andrea Scelfo, Daniel Fernández-Pérez, Simone Tamburri, Marika Zanotti, Elisa Lavarone, Monica Soldi, Tiziana Bonaldi, Karin Johanna Ferrari, Diego Pasini

**Affiliations:** 1 IEO European Institute of Oncology IRCCS, Department of Experimental Oncology, Via Adamello 16, 20139 Milan, Italy; 2 University of Milan, Department of Health Sciences, Via A. di Rudinì, 8, 20142 Milan, Italy

**Keywords:** Polycomb, PCGF, PRC1, USF1, H2A ubiquitination, RING1B, MGA, EZH2, MYC, H3K27me3

## Abstract

Polycomb repressive complexes 1 and 2 (PRC1 and PRC2) control cell identity by establishing facultative heterochromatin repressive domains at common sets of target genes. PRC1, which deposits H2Aub1 through the E3 ligases RING1A/B, forms six biochemically distinct subcomplexes depending on the assembled PCGF protein (PCGF1–PCGF6); however, it is yet unclear whether these subcomplexes have also specific activities. Here we show that PCGF1 and PCGF2 largely compensate for each other, while other PCGF proteins have high levels of specificity for distinct target genes. PCGF2 associates with transcription repression, whereas PCGF3 and PCGF6 associate with actively transcribed genes. Notably, PCGF3 and PCGF6 complexes can assemble and be recruited to several active sites independently of RING1A/B activity (therefore, of PRC1). For chromatin recruitment, the PCGF6 complex requires the combinatorial activities of its MGA-MAX and E2F6-DP1 subunits, while PCGF3 requires an interaction with the USF1 DNA binding transcription factor.

## Highlights

• PRC1 complexes retain high target specificity with little compensatory functions
• PCGF2 defines repressive signatures and PCGF3 and PCGF6 transcription active states
• RING1A/B is dispensable for the assembly and recruitment of PCGF3 and PCGF6 complexes
• PCGF3 and PCGF6 complexes bind target genes through specific DNA binding activities

## Graphical Abstract

**Figure:**
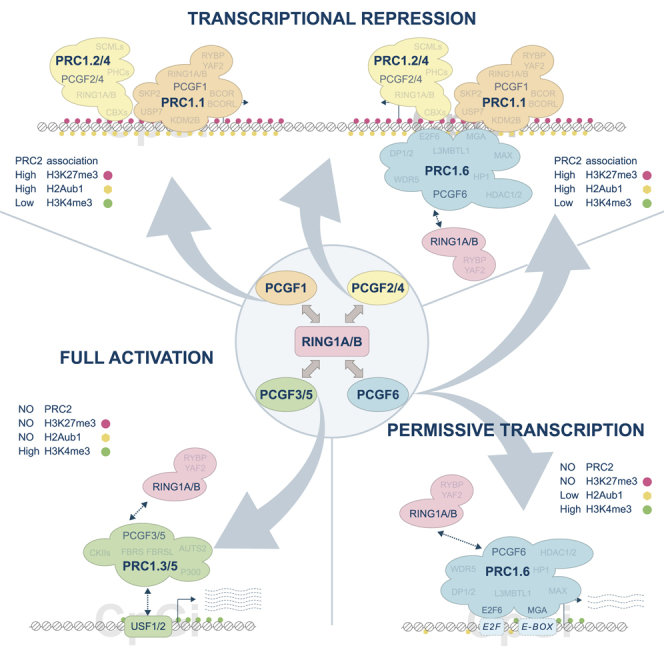

## Teaser

PRC1 encompasses different sub-complexes with distinct biochemical properties. Scelfo et al. dissect these activities, highlighting their little redundancy and high functional specificity. PRC1 sub-complexes associate specifically with transcriptional repression or activation, can function independently of the core RING1A/B enzymes, and can be recruited to chromatin by specific DNA binding activities.

## Introduction

The precise control of specific active and repressed transcriptional states is at the basis of first establishing and then maintaining cellular identity (Bracken and Helin, 2009, Orkin and Hochedlinger, 2011). The Polycomb group (PcG) protein family provides the major repressive mechanism for defining facultative heterochromatin (Bernstein et al., 2006), an essential step for both embryogenesis and homeostatic development of adult tissues (Aloia et al., 2013, Avgustinova and Benitah, 2016). Polycomb proteins exert their functions in two large multiprotein repressive complexes, PRC1 and PRC2, which are defined by specific core activities that modify histone proteins. PRC2 deposits mono-, di-, and tri-methylation on the lysine 27 of histone H3 (H3K27me1/me2/me3), catalyzed by the EZH1/2 methyltransferases (Ferrari et al., 2014, Margueron et al., 2008, Shen et al., 2008); PRC1 mono-ubiquitinates histone H2A lysine 119 (H2Aub1), which is catalyzed by the E3 ligase RING1A or RING1B (de Napoles et al., 2004, Endoh et al., 2008). These two activities control common regulatory pathways by co-associating to a large extent at the same set of target genes (Simon and Kingston, 2013).

Although core enzymatic activities are conserved, PRC1 and PRC2 form distinct subcomplexes defined by the association of ancillary subunits (Scelfo et al., 2015). In PRC1, RING1A/B can interact with one of six distinct, mutually exclusive members of the PCGF protein family (PCGF1–PCGF6), thereby creating six distinct PRC1 subcomplexes (PRC1.1–PRC1.6) that dictate the recruitment of specific ancillary subunits with diverse functional properties (Di Croce and Helin, 2013). Importantly, PCGF2 and PCGF4, or PCGF3 and PCGF5, independently assemble biochemically identical complexes bearing redundant functional properties (Gao et al., 2012). Reasonably, up to four major activities for PRC1 could exist and be active in the same cells, in close relationship with PRC2, but with potentially distinct functions (Pasini and Di Croce, 2016).

PRC1.2 and PRC1.4 are also termed the canonical PRC1 complexes (and the other subcomplexes, non-canonical), based on H3K27me3 recognition deposited by PRC2 (Scelfo et al., 2015). Specifically, the CBX proteins in PRC1.2/PRC1.4 (not present in the other subcomplexes; Gao et al., 2012) bind the H3K27me3 moiety via their chromodomain (Cao et al., 2002, Fischle et al., 2003, Min et al., 2003). How non-canonical subcomplexes are recruited to chromatin remains less well understood. Recruitment of PRC1.1 depends on its KDM2B subunit, which can recognize unmethylated CpG islands (Blackledge et al., 2014). The PRC1.6 complex contains different proteins with DNA binding activity (E2F6-DP1 and MGA-MAX dimers) that could allow direct binding to DNA (Gao et al., 2012). In contrast, the PRC1.3 and PRC1.5 complexes contain no subunits with defined DNA or chromatin binding properties, and their recruitment mechanisms remain unclear.

The activities of non-canonical PRC1 complexes can promote the recruitment and/or stabilization of PRC2 to chromatin (Blackledge et al., 2014, Farcas et al., 2012). This involves the intrinsic ability of PRC2 to bind H2Aub1 deposited by RING1A/B (Cooper et al., 2016, Kalb et al., 2014).

Loss of RING1A/B activity results in pre-implantation lethality at the two-cell stage (Posfai et al., 2012). All distinct PCGF activities play major but distinct roles in development (Akasaka et al., 2001, Almeida et al., 2017, Endoh et al., 2017) (http://www.mousephenotype.org/data/genes/MGI:1917087). Indeed, none of them independently reproduce the loss of RING1A/B activity, suggesting that distinct PCGF activities may act together to determine RING1A/B biological functions. In this context, several questions still remain unanswered. How are the activities of the distinct PRC1 subcomplexes regulated? Do they act redundantly to control similar pathways, or do they (also) have specific functional features?

Here, we provide a comprehensive dissection of the functional landscape of PCGF proteins, characterizing the crosstalk among the different complexes, their relationship with PRC2 activity, and the recruitment mechanisms that mediate their interactions with chromatin and DNA. By combining the development of highly specific PCGF1-6 antibodies with the generation of KO mouse embryonic stem cells (ESC) lines, we mapped the physiological genome-wide occupancy of all PRC1 subcomplexes to determine their functional control. We show that PCGF proteins retain high levels of binding specificity, with little crosstalk among the different complexes with the exception of PCGF1 and PCGF2, which displayed extensive functional overlap. We demonstrate that, while PCGF1 and PCGF2 activities are strongly linked with transcriptional repression, PCGF3 and PCGF6 are mainly associated with active transcriptional states, even in the absence of RING1A/B recruitment. Importantly, the PCGF3- and PCGF6-containing complexes did not require RING1A/B for their assembly and recruitment to chromatin, providing evidence that both complexes are recruited to target genes by intrinsic and specific DNA binding modules.

## Results

### Distinct PRC1 Subcomplexes Regulate Specific Sets of Target Genes

We analyzed both transcription levels and mass spectrometry to determine the relative amounts of distinct PRC1 subcomplexes active in ESCs and found that the PCGF6- and PCGF2-containing complexes were the most abundant forms of PRC1 (Figures S1A and S1B). PCGF4 and PCGF5 were only present in trace amounts, consistent with their poor expression in ESCs. These results agreed with previous measurements (Kloet et al., 2016) and further showed that the functional PRC1 subcomplexes in ESCs are defined by the presence of PCGF1, PCGF2, PCGF3, and PCGF6, confirming that all four functionally distinct forms of PRC1 are present in ESCs.

**Figure 1:**
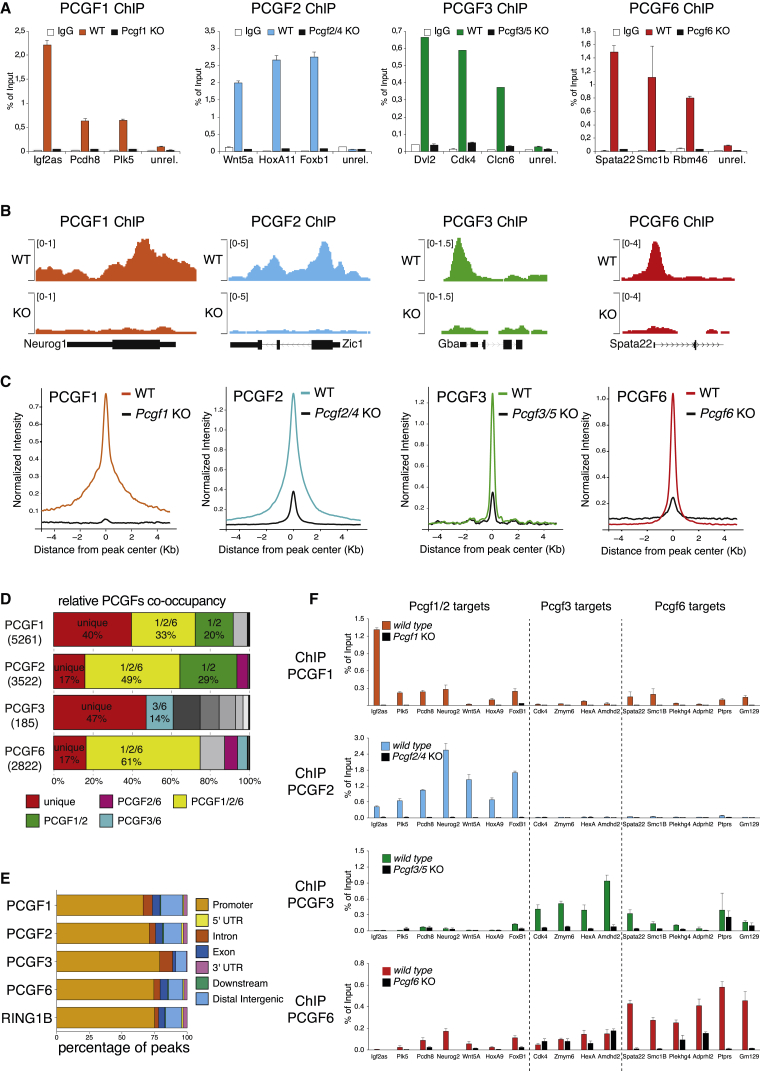
PCGFs Show Specificity in Target Gene Occupancy (A) ChIP-qPCR analysis for the indicated PCGF proteins at selected target regions in wild-type (WT) and in indicated *Pcgf* KO mouse ESCs. IgG served as control for ChIP assay. ChIP enrichments are normalized to input. Data represent mean ± SEM. (B) Genomic snapshots of the indicated ChIP-seq profiles at selected gene loci performed as in (A). (C) ChIP-seq cumulative enrichment deposition centered at peak summit for the indicated PCGF proteins performed as in (A). (D) Percentage of co-occupancy of the target genes identified for each indicated PCGF protein with respect to the other datasets. For simplicity, just regions that represent 14% or more of the total PCGF targets are shown in the legend. (E) Genome-wide functional annotation of peaks generated from the indicated ChIP-seq analyses. Promoters are defined as the region around ±2.5 kb from mm9-annotated TSS, and the downstream regions as the first 3 kb after the TES. (F) ChIP-qPCR analysis for the indicated PCGF proteins at selected target regions in the indicate mESC lines. See also Figure S1 and Tables S2 and S3.

We developed highly specify antibodies for each PCGF protein and engineered ESC lines to individually create *Pcgf1* and *Pcgf6* knockouts (KO) and *Pcgf2/4* and *Pcgf3/5* double-KO (STAR Methods; Table S1) to avoid any potential compensatory effects of redundant PCGF proteins (Figures 1A and 1B) and mapped the PCGF1, PCGF2, PCGF3, and PCGF6 occupancies along the ESC genome by ChIP-seq assays (Figures 1B and 1C). We found that PCGF1 had the most extensive binding repertoire, with 5,261 target genes, followed by PCGF2 (3,522), PCGF6 (2,822), and PCGF3 (185) (Figure 1D). These differences were not due to diverse antibodies efficiencies (Figures S1C and S1D) and did not echo the relative abundance of subcomplexes (Figure S1B). Similar to RING1B, all PCGF proteins preferentially associated to promoter elements (> 75%; Figure 1E) and showed affinity for high CpG dinucleotides density (Figure S1E). PCGF2 occupied broader regions while other PCGF proteins displayed sharper associations, suggesting different modes of chromatin interactions (Figure S1F). By overlapping the enriched genomic regions of each PCGF protein, we found that more frequent combinations of promoter co-occupancy emerged (e.g., PCGF1/2 and PCGF1/2/6) (Figures 1D and S1G). However, these results demonstrated that PCGF proteins also retain high specificity in genomic occupancy, as confirmed by ChIP-qPCR analysis (Figure 1F).

### PCGF Proteins Associate with Distinct Functional Domains

**Figure 2:**
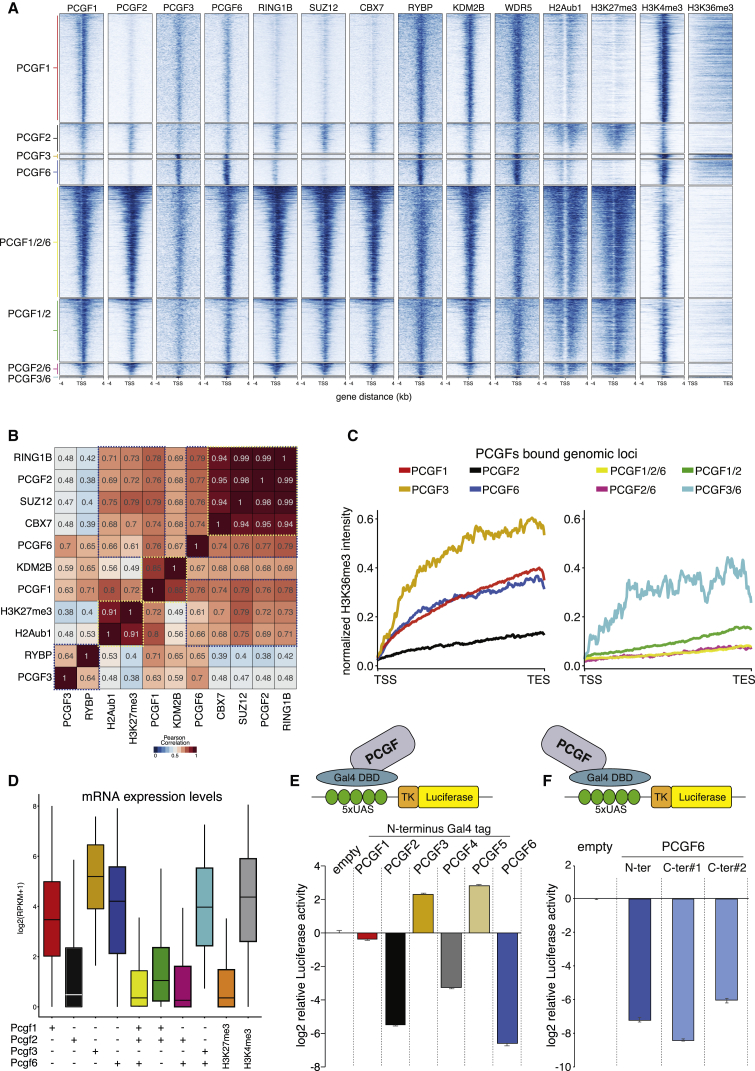
Specific PCGF Activities Define Activating and Repressive Modules (A) Heatmaps representing the normalized ChIP-seq intensities for the indicated PCGF proteins over ±4 kb around the TSS of the indicated loci stratified for PCGF co-occupancy in wild-type (WT) mESCs. H3K36me3 intensity was analyzed over the entire gene length (from TSS to TES). CBX7 and RYBP datasets from mESCs were obtained from Morey et al. (2013) and KDM2B from Farcas et al. (2012). (B) Pearson correlation of ChIP-seq signal over the promoter regions (±4 kb from TSS) of annotated RefSeq coding genes (mm9). (C) Average deposition profile of H3K36me3 in WT mESCs over the gene body (from TSS to TES) of PCGF unique bound promoters (left panel) or promoters co-occupied by at least two PCGF proteins (right panel), as indicated. (D) Boxplots showing the expression levels obtained from RNA-seq analyses performed in WT mESC for the indicated PCGF target genes. H3K27me3- and H3K4me3-positive loci served as controls for repressed and active promoters, respectively. (E) Upper panel: GAL4-TK-luciferase reporter system of 293TRex clones expressing inducible Gal4 (empty) or the indicated Gal4-PCGF fusion protein. Lower panel: Luciferase activity triggered by Gal4-fusion recruitment at GAL4-TK-Luciferase promoter is shown as the fold difference relative to the empty control. Luciferase activity was normalized to protein content. Data represent mean ± SEM. (F) Upper panel: GAL4-TK-luciferase reporter system of 293TRex clones expressing inducible Gal4 (empty) or the indicated Gal4-PCGF6 fusion protein. Lower panel: Luciferase assay (as in E) with PCGF6 N-terminally or C-terminally fused to the DNA binding domain of Gal4. The activity is shown as the fold difference relative to the empty control and was normalized to protein content. Data represent mean ± SEM. See also Figure S2 and Table S4.

We next examined whether distinct PCGF proteins associate to promoter regions that have similar or unique functional properties. First, we defined promoters exclusively-occupied and co-occupied by different PCGFs (Figures 2A and S2A; Tables S2 and S3). Then, we analyzed, on those regions, the presence of general components of the two Polycomb machineries (SUZ12, RING1B, RYBP, and CBX7); WDR5, component of several multiprotein complexes including PRC1.6, COMPASS, and basal transcriptional machineries (Guarnaccia and Tansey, 2018); the unmethylated CpG binding protein KDM2B (Farcas et al., 2012); and histone post-translational modifications (PTMs) associated with activation (H3K4me3 and H3K36me3) or repression (H3K27me3 and H2Aub1) (Figures 2A and S2A). We found that PCGF2 was always associated with a Polycomb repressive signature and that, in the absence of PCGF2, PCGF3, and PCGF6, were associated with a transcriptional permissive status (e.g., high H3K4me3 and H3K36me3), with PCGF3 target genes showing the highest transcriptional activity (Figures 2B and 2C). Similarly, only PCGF1 unique targets, devoid of PCGF2 co-occupancy, displayed a permissive transcriptional activity (Figure 2C), strengthening the correlation between PCGF2 binding and transcriptional repression. These results were further confirmed by transcriptional RNA-seq analyses (Figure 2D).

We next assayed the transcriptional properties of PCGF proteins by independent tethering (as GAL4-PCGF chimeras) to an ectopic artificial promoter that controlled the luciferase gene (as a readout). PCGF2 and PCGF4 strongly repressed luciferase expression, while PCGF3 or PCGF5 activated it (Figure 2E), consistent with their association with repressed and actively transcribed genes, respectively (Figure 2D). PCGF6 strongly repressed luciferase expression (Figures 2E and 2F), suggesting that this complex could have repressive properties also at its transcribed target promoters. MS analysis of the Gal4-PCGF fusions demonstrated normal complexes assembly (Figure S2B; Table S4) to all previously described PRC1 partner proteins (Gao et al., 2012).

Gene ontology for PCGF2-associated genes showed a significant enrichment for development-related processes, irrespective of their co-occupancy with other PCGF proteins (Figure S2C). In contrast, PCGF3- and PCGF6-associated genes were enriched for distinct ontologies related to autophagy or meiosis/spermatogenesis, respectively, denoting distinct functional properties (Figure S2C).

### PCGF Loss Does Not Result in Functional Compensation but Influences PRC1 Subcomplex Activity

**Figure 3:**
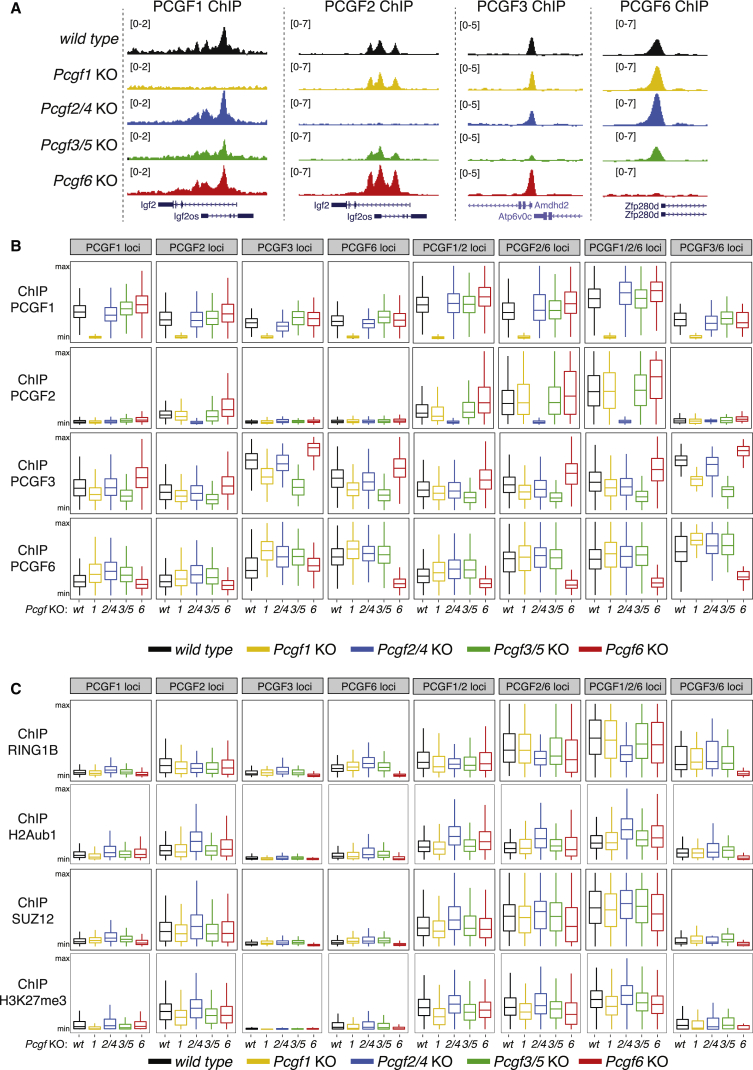
PCGFs Are Specific with Little Compensatory Crosstalk (A) Genomic snapshots of the indicated ChIP-seq profiles at selected target gene loci, performed in WT and in the indicated *Pcgf* KO mESC clones. (B and C) Boxplots of the normalized intensity profiles of ChIP-seq analyses for PCGF1, PCGF2, PCGF3, or PCGF6 (B) and for RING1B, H2AK119ub1 (H2Aub1), SUZ12, or H3K27me3 (C), performed in WT mESCs, *Pcgf1*, *Pcgf2/4*, *Pcgf3/5*, or *Pcgf6* KO ESC clones. Signal enrichment was calculated using a region ±4 kb at unique and co-occupied target genes, as indicated. See also Figures S3 and S4.

To determine whether PRC1 subcomplexes had specific compensatory features, we quantified the chromatin association of PCGF1, PCGF2, PCGF3, and PCGF6 in all the *Pcgf* KO ESC lines by ChIP-seq (Figures 3A). PCGF protein levels and core components of PRC1 and PRC2 remained overall stable (Figures S3A and S3B). Consistently, PCGF proteins’ occupancy was not altered at their specific binding sites (Figures 3B, S3C, and S3D), an effect that was confirmed using a reference genome (ChIP-RX) for signal normalization (Figures S4A and S4B). PCGF proteins did not compensate for each other. For instance, PCGF2 did not bind at PCGF6 target sites in *Pcgf6* KO ESCs (Figures 3B, S4A, and S4B). Similarly, PCGF1 was not affected at PCGF2-target regions in *Pcgf2/4* KO ESCs and vice versa. Overall, these data showed that, in absence of specific PCGF activities, target specificity is largely maintained.

We next quantified binding of RING1B (for PRC1) and SUZ12 (for PRC2), as well as deposition of H2Aub1 and H3K27me3, at each specific group of targets (Figures 3C and S4C). Regions occupied by PCGF2, regardless of which PCGF protein was co-associated, presented much higher RING1B and SUZ12 association as well as H2Aub1 and H3K27me3 deposition than those occupied by PCGF3 and PCGF6 (Figures 3C and S4C). RING1B association was reduced at PCGF2 binding sites in *Pcgf2/4* KO, as well as at PCGF6 binding sites in *Pcgf6* KO. PCGF1 loss had no effect on RING1B association, likely due to PCGF2 compensation. However, RING1B association was only partially lost in *Pcgf2/4* KO, likely compensated by PRC1.1. H2Aub1 deposition was maintained at targets in *Pcgf1* and *Pcgf2/4* KOs, suggesting full compensation between PCGF1 and PCGF2. Differently, RING1B association and H2Aub1 deposition were specifically lost at PCGF3/6 or PCGF6 targets in *Pcgf6* KO ESCs (Figures 3C and S4C). Together, these results revealed that PCGF1 and PCGF2 could compensate for each other specifically at their repressed co-occupied sites but that PCGF6 independently controlled RING1B activity and PRC2 recruitment at sites with substantial transcriptional activity.

### PCGF1/2/4 Module Preserves H2Aub1 Deposition at Repressed Sites but Is Dispensable for ESC Viability

**Figure 4:**
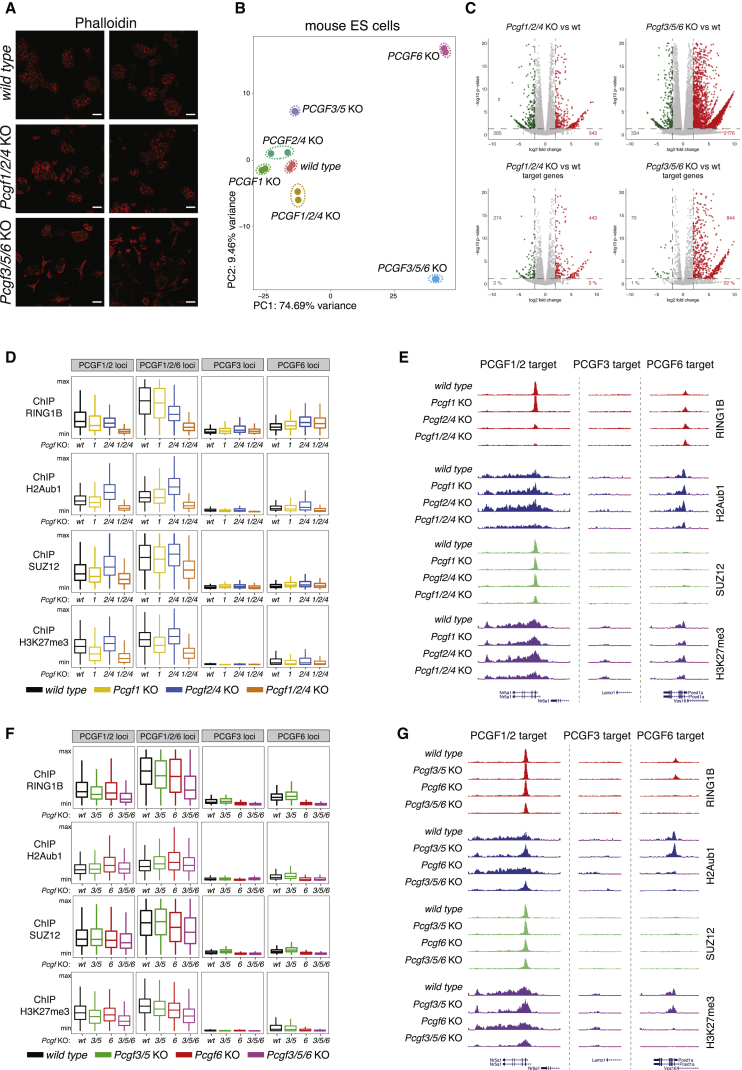
PCGF1 and PCGF2 Compensate H2Aub1 Deposition at Specific Targets and Are Dispensable for ESC Viability (A) Phalloidin immunofluorescence staining in wild-type, *Pcgf1/2/4*, and *Pcgf3/5/6* triple KO mESC. Scale bars correspond to 30 μm. (B) Principal component analysis of gene expression levels from RNA-seq analysis performed in WT mESCs and in the indicated KOs. Dashed lines enclose experimental replicates. (C) Volcano plots of –log10 (p value) against log2 fold change representing the differences in gene expression between *Pcgf1/2/4* and *Pcgf3/5/6* KO mESC clones and WT for all protein coding genes (upper panels) and for PCGF1 and PCGF2 targets or PCGF3 and PCGF6 targets, respectively (bottom panels). (D) Boxplots of normalized ChIP-seq intensity profiles of RING1B, H2AK119ub1 (H2Aub1), SUZ12, and H3K27me3 performed for WT or *Pcgf1*, *Pcgf2/4*, and *Pcgf1/2/4* KO mESC clones, over ±500 bp (or ±4 kb for H2Aub1 and H3K27me3) around the TSS of target genes unique for PCGF3 or PCGF6, or common to PCGF1/2 or PCGF1/2/6. (E) Genomic snapshots of the ChIP-seq profiles quantified in (D) at selected target gene loci (common or unique, as indicated), performed in WT and the indicated *Pcgf* KO mESC clones. (F) Boxplots of normalized ChIP-seq intensity profiles of RING1B, H2AK119ub1 (H2Aub1), SUZ12, and H3K27me3 performed in WT or *Pcgf6*, *Pcgf3/5*, and *Pcgf3/5/6* KO mESC clones over ±500 bp (or ±4 kb for H2Aub1 and H3K27me3) around the TSS of unique or common target genes (as indicated). (G) Genomic snapshots of the ChIP-seq profiles quantified in (F) at selected common or unique target gene loci (as indicated), performed in WT and the indicated *Pcgf* KO mESC clones. See also Figures S5, S6, and S7 and Tables S5 and S6.

Based on these findings, we defined PCGF1/2/4 activity as the PRC1 repressive module and PCGF3/5/6 as the PRC1 activating module, and generated *Pcgf*1/2/4 and *Pcgf3*/5/6 triple KO ESCs (Figures S5A and S5B). While *Pcgf1/2/4* KO showed no effects on cell viability, *Pcgf3/5/6* KO clones displayed severe morphological changes acquiring a flattened fibroblast-like shape (Figures 4A and S5C). Principal component analysis (PCA) from RNA-seq profiles showed that the transcriptome from *Pcgf1/2/4* KO cells was largely unaltered as compared to wild-type, *Pcgf1* KO, or *Pcgf2/4* KO ESCs (Figures 4B, 4C, S5D, and S5E; Table S5). Loss of *Pcgf6* alone induced significant phenotypic changes, in agreement with previous reports (Yang et al., 2016), enhanced by concomitant loss of PCGF3/5 activities (*Pcgf3/5/6* KO). RNA-seq analyses showed alterations in expression of genes implicated with various developmental processes, overall highlighting a compromised ESC identity (Figures 4B, 4C, S5D, and S5E). However, this occurred in the absence of a major induction of early differentiation programs (Figure S6) (Hutchins et al., 2017). In particular, *Pcgf3/5/6* KO ESCs were characterized by extracellular matrix and cornification enriched ontologies (Figure S7A; Table S6) that corresponded to a reorganization of actin fibers (Figure 4A) and massive upregulation of collagens and keratins (Figures S7B and S7C).

Combined loss of the PCGF1/2/4 resulted in complete RING1B displacement and loss of H2Aub1 specifically at all PCGF2-occupied loci (Figures 4D, 4E, S7D, and S7E), remaining unaltered in *Pcgf3/5/6* KOs (Figures 4F, 4G, S7F, and S7G). This further correlated with decreased PRC2 (SUZ12) association and reduced H3K27me3 deposition (Figures 4D, 4E, S7D, and S7E). Lack of H2Aub1 deposition did not result in a significant activation of these targets (Figures 4B and 4C). In contrast, loss of PCGF6 led to diffuse gene upregulation (Figures 4B, 4C, and S7H), which occurs in absence of PCGF1/2 displacement and loss of H2Aub1 from co-occupied promoters (Figure 3B, S4C, S5A, and S5B). This supports Gal4-PCGF6 repressive activity and the H2Aub1-independent transcriptional properties of PRC1 (Illingworth et al., 2015, Pengelly et al., 2015).

### RING1A/B Mediates Complex Assembly and Chromatin Binding for PCGF1 and PCGF2, but Not for PCGF3 and PCGF6

**Figure 5:**
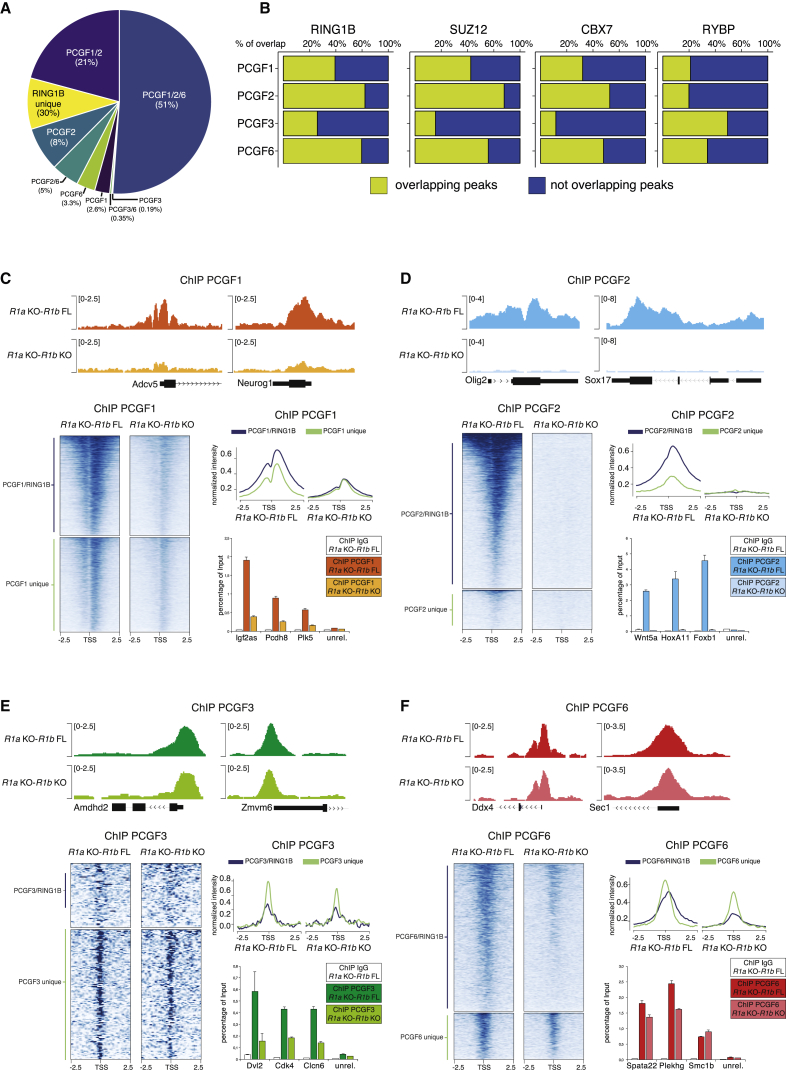
PCGF3 and PCGF6 Activities Are Independent of RING1A/B (A) Percentage of occupancy of the different PCGF proteins at RING1B-bound promoters. (B) Percentage of overlap of RING1B, SUZ12, CBX7, and RYBP at the indicated PCGF-bound promoters. (C) Upper panel: Genomic snapshots of PCGF1 ChIP-seq profiles at selected genomic regions performed in *Ring1A*^*–/–*^*;Ring1B*^*fl/fl*^ (*R1A* KO-*R1B* FL) and *Ring1A*^–/–^;*Ring1B*^–/–^ (*R1A* KO-*R1B* KO) mESCs. Bottom left: Heatmap showing the normalized signal of PCGF1 ChIP-seq in *R1A* KO-*R1B* FL and *R1A* KO-*R1B* KO mESC over ±2.5 Kb of PCGF1/RING1B and PCGF1/2/RING1B common target loci, as well as PCGF1 and PCGF1/2 unique target loci. Bottom right: Cumulative quantification of the heatmaps and PCGF1 ChIP-qPCR analysis at selected regions performed in the same mESCs. IgG served as ChIP negative control. ChIP enrichments were normalized to input. Data represent mean ± SEM. (D) As in (C), for PCGF2 ChIP-seq profiles analyzed at the indicated PCGF2-specific targets. (E) Upper panel: Genomic snapshots of PCGF3 ChIP-seq profiles at selected genomic regions performed in *R1A* KO-*R1B* FL and *R1A* KO-*R1B* KO mESC. Bottom left: Heatmap showing the normalized signal of PCGF3 ChIP-seq in *R1A* KO-*R1B* FL and *R1A* KO-*R1B* KO mESCs over ±2.5 Kb of PCGF3/RING1B common target loci and PCGF3 unique target loci. Bottom right: Cumulative quantification of the heatmaps and PCGF3 ChIP-qPCR analyses at selected regions performed in the same ESCs. IgG served as ChIP negative control. ChIP enrichments were normalized to input. Data represent mean ± SEM. (F) As in (E), for PCGF6 ChIP-seq profiles analyzed at the indicated PCGF6-specific targets. See also Figure S8.

A detailed quantification of RING1B co-occupancy with each PCGF protein showed that 70% of all RING1B binding sites overlapped with at least one PCGF protein (Figure 5A). Approximately 60%, 40%, and 80% of PCGF1-, PCGF6-, and PCGF3-bound genomic loci, respectively, were not significantly enriched for RING1B association (Figure 5B). This lack of RING1B co-occupancy correlated with (1) a lack of repressive marks (H3K27me3 or H2Aub1) and (2) an accumulation of an activating signature (H3K4me3 and H3K36me3) (Figures S8A–S8E). These observations suggested that RING1B was indeed absent from these sites. To gain further evidence for the role of RING1B in regulating different PCGF complexes functions, we performed ChIP-seq analysis for each PCGF protein after RING1A/B loss of function (LoF) using a *Ring1A*^–/–^; *Ring1B*^fl/fl^; *Rosa26::CreERT2* conditional mouse ESC line (Endoh et al., 2008) (termed herein *R1A* KO-*R1B* FL). Treatment with 4-hydroxy tamoxifen (OHT) conditionally deletes *Ring1B*, leading to complete loss of RING1B expression within 48 hr (Figure S8F). ChIP-seq analysis for PCGF1 and PCGF2 at 60 hr after OHT treatment showed global displacement of both proteins from chromatin, demonstrating that RING1A/B expression is essential for PCGF1/2 recruitment (Figures 5C, 5D, S8G, and S8H). PCGF2 levels were strongly destabilized in the absence of RING1A/B, suggesting that RING1A/B are required for the assembly of the PRC1.2 complex (which mediates the stabilization of PCGF2) (Figure S8F). In contrast, similar analyses for PCGF3 and PCGF6 revealed that loss of RING1A/B expression did not affect their genome-wide localization (Figures 5E and 5F). Together, these results strongly suggested that while RING1A/B plays an essential role in the assembly and recruitment of the PRC1.1 and PRC1.2 complexes, it is dispensable for PCGF3 and PCGF6 chromatin association. This reinforces the possibility that several PCGF3 and PCGF6 targets do not require RING1A/B association.

### E2F and the E-Box Motif Cooperate to Recruit the PCGF6 Complex to Target Sites

**Figure 6:**
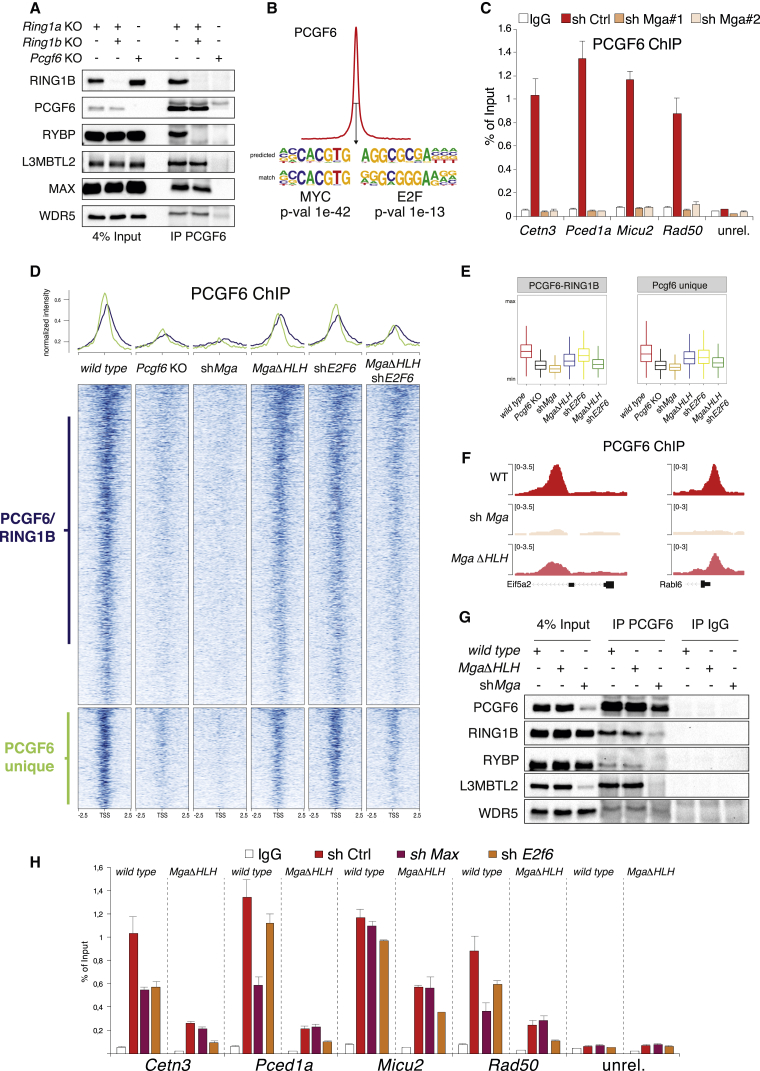
PCGF6 Requires Cooperative E2F and E-Box Recognition for Target Recruitment (A) Western blot analyses using the indicated antibodies in PCGF6 immunoprecipitations from nuclear extracts prepared from the indicated mESC lines. Input served as loading control. (B) *De novo* motif discovery analysis performed underneath the summit of PCGF6 peaks. Sequence weight matrixes of predicted compared to match DNA binding motifs are shown together with p values. (C) PCGF6 ChIP-qPCR analyses on mESC expressing scrambled (sh Ctrl) or *Mga*-specific shRNAs at PCGF6 target and a negative region (neg). IgG served as ChIP negative control. ChIP enrichments are normalized to input. Data represent mean ± SEM. (D) Normalized intensity profiles and heatmap of PCGF6 binding in WT mESCs or *Pcgf6* KO, a sh*Mga*, *MgaΔ*HLH mutant, sh*E2f6*, and a sh*E2f6* in *MgaΔ*HLH mutant around ±2.5 kb of the TSS of common and unique target loci. (E) Boxplots of the normalized intensity profiles of ChIP-seq analyses for PCGF6 in WT or *Pcgf6* KO mESCs, a sh*Mga*, *MgaΔ*HLH mutant, sh*E2f6*, and a sh*E2f6* in *MgaΔ*HLH mutant over ±500 bp respective to the TSS of common and unique target loci. (F) Genomic snapshots of the PCGF6 ChIP-seq profiles at selected genomic regions in WT, sh*Mga*, and *MgaΔ*HLH mESCs. (G) Western blot analyses using the indicated antibodies in PCGF6 immunoprecipitations from nuclear extracts prepared from WT or sh*Mga*, *MgaΔ*HLH mESCs. IgG served as an unrelated control antibody. Input is shown as loading control. (H) PCGF6 ChIP-qPCR analysis on WT mESCs and *MgaΔ*HLH mESCs expressing scrambled (Ctrl), *Max,* or *E2F6*-specific shRNAs at PCGF6 target and a negative region (unrel). IgG served as ChIP negative control. ChIP enrichments are normalized to input. Data represent mean ± SEM. See also Figure S9.

To further explore the properties of the PRC1.6, we investigated its biochemical and recruitment features. Co-immunoprecipitation (coIP) analyses for PCGF6 in *R1A* KO-*R1B* FL ESCs (treated or not with OHT) and *Pcgf6* KO ESCs revealed that, consistently with ChIP-seq results, components of the PRC1.6 remained associated with PCGF6 even in the absence of RING1A/B expression (e.g., L3MBTL2, MAX, and WDR5) (Figure 6A). We performed *de novo* motif discovery on PCGF6 peaks and found that MYC (E-box) and E2F sites were the most enriched and represented DNA motifs (Figure 6B). This strongly agrees with the presence of E2F6-DP1 and MGA-MAX heterodimers among the components of the PCGF6 complex (Hauri et al., 2016, Ogawa et al., 2002). Indeed, PCGF6 occupancy in ESCs almost completely overlapped with MGA genomic distribution (Stielow et al., 2018) (Figure S9A). This result was further confirmed by ChIP-qPCR performed with MGA and E2F6 antibodies (Figure S9B). To further explore the contribution of these DNA binding activities in PCGF6 recruitment, we knocked down MGA using different shRNA sequences (Figures S9C and S9D). Notably, PCGF6 was displaced from all its target sites upon MGA downregulation, as shown by ChIP analysis for PCGF6 (Figure 6C). However, in an ESC line with a MGA C-terminal deletion of the HLH domain (*Mga*ΔHLH), PCGF6 binding was only moderately affected (∼2-fold; Figures 6D–6F). These results demonstrated that E-box recognition was not sufficient to impair PCGF6 recruitment but suggested that MGA has broader structural functions in mediating proper PCGF6 complex assembly. CoIP analysis for PCGF6 in both *Mga*ΔHLH ESCs or after *Mga* shRNA-mediated knockdown revealed that the MGAΔHLH mutation did not affect the association of PCGF6 with its different interacting partners but that downregulation of MGA expression disrupted the PCGF6 complex (Figure 6G).

We next tested whether the E2F6-DP1 dimer provided additional DNA binding affinity to the PCGF6 complex. We induced loss of E2F6 or MAX expressing specific shRNAs in wild-type or *Mga*ΔHLH mutant ESCs (Figures S9E and S9F) and analyzed PCGF6 chromatin association by ChIP. The combined loss of MGA and E2F6 DNA binding activities further reduced PCGF6 recruitment to its target sites (Figures 6D, 6E, 6H, S9G, and S9H). This strongly suggests that E2F and E-box recognition by intrinsic subunits of the PCGF6 complex mediates DNA binding specificity. It is important to highlight that PCGF6 binding was clearly affected but not completely abolished by E2F6 shRNA (Figures 6D, 6E, and 6H). Although this could be a consequence of an incomplete loss of E2F6 expression (Figure S9F), this result may also suggest that additional recruitment mechanisms (i.e., via L3MBTL2; Huang et al., 2018, Trojer et al., 2011) contribute to recruiting the PCGF6 complex to its specific target loci.

### USF1/2 Interacts with the PCGF3 Complex and Mediates Its Chromatin Recruitment

**Figure 7:**
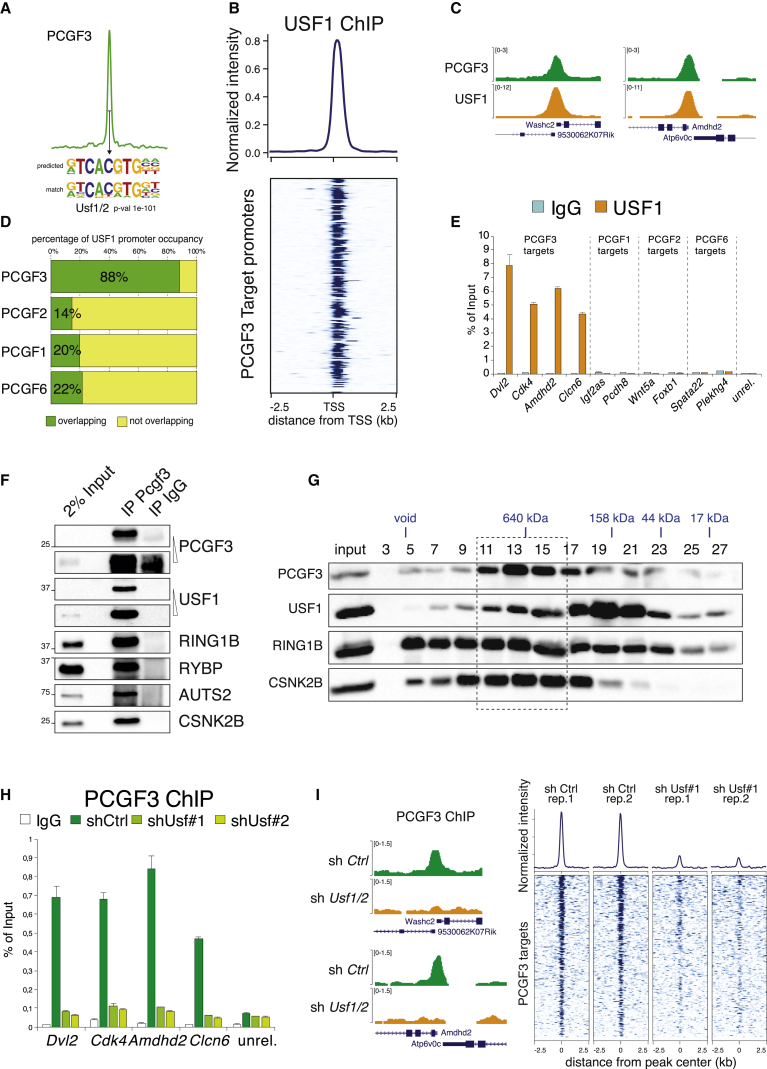
Interactions with USF1/2 Mediate PCGF3 Recruitment to Chromatin (A) *De novo* motif discovery analysis performed underneath the summit of PCGF3 peaks. Sequence weight matrixes of predicted compared to match DNA binding motifs are shown together with relative p values. (B) Normalized ChIP-seq intensity profile of USF1 binding over ±2.5 kb regions of PCGF3 target loci. (C) Genomic snapshots of PCGF3 and USF1 ChIP-seq profiles at PCGF3 targets in WT mESCs. (D) Relative (percentage) of the different PCGF target genes co-occupied by USF1. (E) ChIP-qPCR analysis for USF1 in WT mESCs at representative PCGF1, PCGF2, PCGF3, and PCGF6 targets. IgG served as a negative ChIP control. ChIP enrichments are normalized to input. Data represent mean ± SEM. (F) Wstern blot analyses using the indicated antibodies in PCGF3 immunoprecipitations from nuclear extracts prepared from WT mESCs. Rabbit IgG served as an unrelated control antibody. Inputs are shown as loading control. (G) Western blot analysis with the indicated antibodies of protein fractions from WT mESC nuclear extracts separated by size-exclusion chromatography. Molecular weights are indicated based on the elution profile of markers in the same conditions. (H) PCGF3 ChIP-qPCR analysis on mESCs expressing scrambled (shCtrl) or combined *Usf1*- and *Usf2* (sh*Usf*)-specific shRNAs. IgG served as negative ChIP control. ChIP enrichments are normalized to input. Data represent mean ± SEM. (I) Genomic snapshots of PCGF3 ChIP-seq profiles in scrambled (shCtrl) and combined *Usf1*- and *Usf2* (sh*Usf*)-specific shRNAs expressing mESCs (left panel) and its relative normalized ChIP-seq intensity profiles for two biological replicates (right panels) over a ±2.5 Kb region at PCGF3/RING1B common and PCGF3 unique target loci. See also Figure S10.

PCGF3 associated with high specificity to a restricted number of transcribed target promoters in a RING1A/B (PRC1)-independent manner. As no evident DNA binding activities were previously associated to the PCGF3 complex (Gao et al., 2012), we performed *de novo* motif discovery analyses on PCGF3 peaks. The only enriched motif perfectly matched an E-box variant corresponding to the DNA binding site of USF1/2, an HLH-containing transcription factor that forms a heterodimer similar to MYC-MAX (Kiermaier et al., 1999) (Figure 7A). In accordance, ChIP-seq analyses of USF1 in wild-type ESCs revealed a strong USF1 enrichment at almost all PCGF3-bound loci (88%; Figures 7B–7D and S10A). This enrichment was specific for PCGF3 and overlapped only marginally with other PCGF binding sites (Figures 7D and 7E). As USF1 has not been previously reported to interact with PCGF3, we tested USF1–PCGF3 interaction using milder coIP conditions after digesting DNA with benzonase. USF1 showed a clear DNA-independent association with the PCGF3 complex (Figures 7F and S10B). Size-exclusion chromatography further confirmed this result: USF1 co-eluted in a high molecular weight fractions (>650 kD) together with several components of the PCGF3 complex (Figure 7G). We next downregulated *Usf1* and *Usf2* expression with different shRNAs in wild-type ESCs (Figures S10C and S10D). ChIP-seq analysis under these conditions showed that PCGF3 was displaced from all PCGF3 target sites in the absence of USF1/2 expression (Figures 7H and 7I). In contrast, DNA binding of USF1 was not affected by loss of PCGF3 at target sites (Figures S10E and S10F). Together, these results demonstrated that USF1 and USF2 function as DNA binding hubs that mediate recruitment of the PCGF3 complex to DNA.

## Discussion

### PCGF Proteins Have High Specificity for Target Genes

Our data provide the first genome-wide analysis of different PCGF protein activities at a physiological level. These results clearly showed that PCGF proteins display high binding specificity with little functional overlap, with the exception of PCGF1 and PCGF2. PCGF1 has a broad pervasive binding at a large set of CpG-rich promoters, which correlates with RYBP binding and with the broad occupancy of KDM2B. Importantly, H2Aub1 deposition was affected only when loss of PCGF1 and PCGF2/4 were combined, suggesting full enzymatic compensation of these complexes without altering their overall recruitment. Based on these results, we speculate that PCGF1 and PCGF2 act redundantly and enzymatically engage the same target sites in the absence of any evident competition. PCGF6 also showed substantial overlap with PCGF1/2 but with a marginal role in compensating H2Aub1 deposition at these sites. The remaining PCGF6 sites essentially lacked co-association of any other PCGF protein, and loss of PCGF6 did not result in significant cross-compensations. Finally, PCGF3 had few but very specific binding sites at promoters, and it only marginally overlapped with PCGF6. Together, these results highlight the functional specificity of the distinct PRC1 subcomplexes, demonstrating that while PRC1.1 and PRC1.2 cooperate to regulate the same pathways, PRC1.3 and PRC1.6 retain high target specificity and little crosstalk with the activity of the other complexes. Surprisingly, combined loss of PCGF1/2/4 and lack of H2Aub1 deposition at repressed genes did not induce a significant transcriptional reactivation. In contrast, loss of PCGF6 activity resulted in a clear reactivation of these targets as well as of transcribed PCGF6 unique targets devoid of PCGF1/2 co-association. Together, these results suggest that PCGF6 plays a general major role in repression. Furthermore, this occurred at PCGF1/2 co-occupied promoters without loss of H2Aub1. This highlights a marginal role of H2Aub1 in repression that supports previous reports that challenged the role of H2Aub1 in regulating PcG spatiotemporal control of target genes expression during development (Illingworth et al., 2015, Pengelly et al., 2015).

### PRC1 Subcomplexes Comprise Repressive or Activating Modules

Our results revealed that the presence of PCGF2 always correlated with full transcriptional repression, regardless of which PCGF protein was co-associated, at target loci involved in developmental processes. This is in line with the co-association of a classical Polycomb signature characterized by abundant H2Aub1 deposition, PRC2 binding, and high H3K27me3 levels. In contrast, PCGF6 unique targets presented a permissive transcriptional state, whereas PCGF3 targets a full transcriptional activity. In addition, our data show that PCGF3 and PCGF6 can both exist in a complex in the absence of RING1A/B association, which correlates with lack of classical PcG chromatin signature. While PCGF6-bound promoters presented a much lower enrichment, PCGF3 sites showed nearly undetectable RING1B and H2Aub1 levels at target loci (enriched for autophagy and lysosomal activity ontologies). However, it is also possible that the high transcriptional status of these targets per se prevents accumulation of repressive marks. Overall, these data showed that the levels of PRC1.2 at target sites correspond well to transcriptional activity, while recruitment of PCGF1, PCGF3, and PCGF6 complexes may be less dependent on the transcriptional status of their target genes. This relates to the passive models of PRC2 recruitment proposed by the Helin and Bernstein laboratories, showing that PRC2 promiscuously binds unmethylated CpG islands until it is excluded by active transcription (Mendenhall et al., 2010, Riising et al., 2014). Indeed, both PRC1.1 and PRC1.2 have (direct or indirect) affinities for CpG-dense regions: KDM2B provides a direct affinity for unmethylated CpGs to PRC1.1 (Farcas et al., 2012), and the H3K27me3 deposited by PRC2 provides a docking site for PRC1.2 (Cao et al., 2002, Fischle et al., 2003, Min et al., 2003). In contrast, the requirement of specific DNA binding activities of PCGF3 and PCGF6 complexes to actively transcribed genes may also suggest distinct biological functions that are different from PCGF1/2.

### PCGF3 and PCGF6 Activity Is Linked to Transcription Activation and Does Not Require RING1A/B Association

While PCGF3 and PCGF5 activated transcription in an artificial assay, PCGF6 behaved as a repressor, in apparent contradiction with its physiological association to actively transcribed targets. Since PCGF6 is recruited to DNA by E-box recognition, we envisioned an antagonistic mechanism between PCGF6 and MYC. MYC shares a large fraction of PCGF6 targets, and, upon loss of PCGF6 functions, a substantial set of transcribed PCGF6 targets increased their transcriptional activity. This suggests that PCGF6 may function as “attenuator” of transcription via yet-uncharacterized mechanisms of regulation that may not involve H2Aub1 deposition. We found that RING1A/B activity was dispensable for both assembly and chromatin recruitment of the PCGF3 and PCGF6 complexes, which could provide insight to a not-yet-identified mechanism regulating the activity of these complexes at target sites in the absence of RING1A/B. Indeed, RING1B recruitment and H2Aub1 deposition were very low at uniquely PCGF6 target sites and were only barely detectable at PCGF3 sites. Based on the crystal structure of the RING1B-PCGF4 (BMI1) heterodimer, PCGF proteins should interact with RING1A/B via dimerization of their respective RING domains (Buchwald et al., 2006). In fact, while this interaction is critical for PRC1.1 and PRC1.2 complexes, it seems to have a poor impact on assembly or recruitment of the PRC1.3 and PRC1.6. Only RYBP association was dependent on RING1A/B, suggesting that RING1A/B-RYBP are recruited to the PCGF3 and PCGF6 complexes as a separate module. How these interactions are regulated, and whether they play any role in the biological function of these complexes, remains to be clarified.

### PCGF6 Is Recruited to DNA by Cooperative Binding to E2F and E-Box Elements

Our data showed that the PCGF6 complex’s affinity for DNA comes from the DNA binding activities of its distinct subunits. The complete displacement of PCGF6 from chromatin upon loss of MGA expression agrees with previous reports (Endoh et al., 2017, Stielow et al., 2018) but was a consequence of complex destabilization rather than a loss of DNA interaction. Thus, MGA also plays an important role as a scaffold to assemble the PCGF6 complex, very similar to the role of L3MBTL2 in this same complex (Stielow et al., 2018). Indeed, a minimal C-terminal truncation of the DNA binding domain of MGA can still assemble a normal PRC1.6, only partially reducing its chromatin recruitment. In these conditions, the additional loss of E2F6 further displaced the PCGF6 complex from chromatin, demonstrating that both DNA binding activities are required for efficient PCGF6 complex target recognition.

### PCGF3 Is Recruited to Chromatin by Its Interaction with USF1/2 DNA Binding Transcription Factors

Among the PRC1 subcomplexes, only the PRC1.3 and PRC1.5 complexes do not contain biochemical modules that provide specific affinity for chromatin states (e.g., CBX proteins) and/or DNA elements (e.g., KDM2B, MGA, or E2F6). Through genome-wide location analysis, we identified a strong enrichment for the USF1/2 DNA binding motif that corresponds to a variant E-box that contains a thymidine at the 5′ of the canonical CACGTG E-BOX motif recognized by MYC (**T**CACGTG). This may explain the moderate but specific enrichment of PCGF3 at some PCGF6 sites. We further demonstrate that USF1 interacts with PCGF3 independently of DNA and that its expression was essential for the recruitment of the PCGF3 complex to all its target sites. USF1/2 is functionally linked with active chromatin states (Pognonec and Roeder, 1991) and, together with the role of the PCGF3 component AUTS2 in mediating P300 recruitment (Gao et al., 2014), directly links the PCGF3 complex to transcriptional activation. It remains unclear how this activatory role is linked with PCGF3/5 mediated regulation of X chromosome inactivation (Almeida et al., 2017). In the future, it would be very interesting to dissect the biochemical basis of PCGF3-USF1 interaction and to know how it is regulated to provide functional specificities.

Altogether, our results provide a first comprehensive analysis of the different PRC1 subcomplexes activities, uncovering their functional specificities that classify them in either repressive or activating modules with defined specific mechanisms of recruitment. Importantly, our data further demonstrate that RING1A/B activity is not essential for the assembly or chromatin recruitment of all PRC1 subcomplexes, suggesting a new potential mechanism of regulation for PCGF3/5 and PCGF6. Based on these latter findings, we speculate that PCGF1 and PCGF2 exert Polycomb-related functions, while PCGF3/5 and PCGF6 activities can also exist in functional forms unrelated to classically defined Polycomb activities.

## STAR★Methods

### Key Resources Table

**Table:** 

REAGENT or RESOURCE	SOURCE	IDENTIFIER
**Antibodies**		

Rabbit anti-Pcgf1	this study	N/A
Rabbit anti-Pcgf2	this study	N/A
Rabbit anti-Pcgf3	this study	N/A
Rabbit anti-Pcgf6	this study	N/A
Rabbit anti-Ring1b	Pasini laboratory	N/A
Rabbit anti-Wdr5	Pasini laboratory	N/A
Mouse anti-Vinculin	Pasini laboratory	N/A
Rabbit anti-Rybp	Millipore	Cat# AB3637; RRID: AB_2285466
Rabbit anti-Cbx7	Abcam	Cat# ab21873; RRID: AB_726005
Rabbit anti-Suz12	Cell Signaling Technology	Cat# 3737S; RRID: AB_2196850
Rabbit anti-H3K27me3	Cell Signaling Technology	Cat# C36B11; RRID: AB_2616019
Rabbit anti-H3K36me3	Cell Signaling Technology	Cat# 4909BF; RRID: AB_2616016
Rabbit anti-H3K4me3	Active Motif	Cat# 39159; RRID: AB_2615077
Rabbit anti-H2AK119ub1	Cell Signaling Technology	Cat# 8240; RRID: AB_10891618
Rabbit anti-L3mbtl2	Active Motif	Cat# 39570; RRID: AB_2615062
Rabbit anti-Max	Santa Cruz Biotechnology	Cat# sc-197; RRID: AB_2281783
Mouse anti-Usf1	Santa Cruz Biotechnology	Cat# sc-101197; RRID: AB_1131108
Rabbit anti-Auts2	ProteinTech	Cat# 25001-1-AP
Rabbit anti-Csnk2b	ProteinTech	Cat# 20234-1-AP
VeriBlot IP Detection Reagent (HRP)	Abcam	Cat# ab131366
Rabbit anti-E2F6	Abcam	Cat# ab53061
Rabbit anti-MGA	Bethyl	Cat# A302-864A
Rabbit anti-GAL4	Santa Cruz Biotechnology	Cat# sc-577

**Bacterial and Virus Strains**		

STBL3	Thermo Fisher Scientific	Cat# C737303
TOP10	Thermo Fisher Scientific	Cat# C404010
BL-21	Thermo Fisher Scientific	Cat# C600003

**Experimental Models: Cell Lines**		

Human: HEK293T	ATCC	ATCC CRL-3216
Human: HEK293T 5XGal4TK-Luc-neo Flp-In	Pasini et al., 2010	N/A
Mouse: ES cell line Ring1A−/−;Ring1Bfl/fl; Rosa26::CreERT2	Endoh et al., 2008	N/A Strain of origin 129P2/Ola
Mouse: ES cell line E14	Pasini laboratory	N/A Strain of origin 129P2/Ola
Mouse: ES cell line E14 Pcgf1 −/−	this study	N/A Strain of origin 129P2/Ola
Mouse: ES cell line E14 Pcgf2 −/−	this study	N/A Strain of origin 129P2/Ola
Mouse: ES cell line E14 Pcgf3/5−/−	this study	N/A Strain of origin 129P2/Ola
Mouse: ES cell line E14 Pcgf6 −/−	this study	N/A Strain of origin 129P2/Ola
Mouse: ES cell line E14 Pcgf2/4 −/−	this study	N/A Strain of origin 129P2/Ola
Mouse: ES cell line E14 Pcgf1/2/4 −/−	this study	N/A Strain of origin 129P2/Ola
Mouse: ES cell line E14 Pcgf3/5/6−/−	this study	N/A Strain of origin 129P2/Ola
Drosophila: S2	ATCC	ATCC CRL-1963

**Oligonucleotides**		

qPCR primer sets	this study	available upon request

**Recombinant DNA**		

pX459 2.0	this study; Table S1	Addgene #62988
pLKO.1	this study; Table S1	Addgene #8453

**Chemicals, Peptides, and Recombinant Proteins**		

Leukemia inhibitory factor	Pasini laboratory	N/A
CHIR99021	Stemcell technologies	Cat# 72052
PD0325901	Stemcell technologies	Cat# 72182
Lipofectamine 2000	Invitrogen	Cat# 11668027
Igepal	Sigma-Aldrich	Cat# I8896
EGS	Sigma-Aldrich	Cat# E3257

**Deposited Data**		

E14-Cbx7 ChIP-Seq	Morey et al., 2013	GEO: GSM1041373
E14-Rybp ChIP-Seq	Morey et al., 2013	GEO: GSM1041375
E14-RNAPolII ChIP-Seq	Riising et al., 2014	GEO: GSM1399506
E14-Mga ChIP-seq	Stielow et al., 2018	ArrayExpress: E-MTAB-6007
E14-Kdm2b ChIP-seq	Farcas et al., 2012	GEO: GSM1003694
E14-H2AK119Ub1 ChIP-Seq	this study	GEO: GSE122715
E14-H3K27m3 ChIP-Seq	this study	GEO: GSE122715
E14-H3K36m3 ChIP-Seq	this study	GEO: GSE122715
E14-H3K4m3 ChIP-Seq	this study	GEO: GSE122715
E14-Pcgf1 ChIP-Seq	this study	GEO: GSE122715
E14-Pcgf2 ChIP-Seq	this study	GEO: GSE122715
E14-Pcgf3 ChIP-Seq	this study	GEO: GSE122715
E14-Pcgf6 ChIP-Seq	this study	GEO: GSE122715
E14-Ring1b ChIP-Seq	this study	GEO: GSE122715
E14-Rybp ChIP-Seq	this study	GEO: GSE122715
E14-Suz12 ChIP-Seq	this study	GEO: GSE122715
E14-Usf1 ChIP-Seq	this study	GEO: GSE122715
E14-Wdr5 ChIP-Seq	this study	GEO: GSE122715
MgadHLH-Pcgf6 ChIP-Seq	this study	GEO: GSE122715
Pcgf124KO-H2AK119Ub1 ChIP-Seq	this study	GEO: GSE122715
Pcgf124KO-H3K27m3 ChIP-Seq	this study	GEO: GSE122715
Pcgf124KO-Ring1b ChIP-Seq	this study	GEO: GSE122715
Pcgf124KO-Suz12 ChIP-Seq	this study	GEO: GSE122715
Pcgf356KO-H2AK119Ub1 ChIP-Seq	this study	GEO: GSE122715
Pcgf356KO-H3K27m3 ChIP-Seq	this study	GEO: GSE122715
Pcgf356KO-Ring1b ChIP-Seq	this study	GEO: GSE122715
Pcgf356KO-Suz12 ChIP-Seq	this study	GEO: GSE122715
Pcgf1KO-H2AK119Ub1 ChIP-Seq	this study	GEO: GSE122715
Pcgf1KO-H3K27m3 ChIP-Seq	this study	GEO: GSE122715
Pcgf1KO-H3K36m3 ChIP-Seq	this study	GEO: GSE122715
Pcgf1KO-H3K4m3 ChIP-Seq	this study	GEO: GSE122715
Pcgf1KO-Pcgf1 ChIP-Seq	this study	GEO: GSE122715
Pcgf1KO-Pcgf2 ChIP-Seq	this study	GEO: GSE122715
Pcgf1KO-Pcgf3 ChIP-Seq	this study	GEO: GSE122715
Pcgf1KO-Pcgf6 ChIP-Seq	this study	GEO: GSE122715
Pcgf1KO-Ring1b ChIP-Seq	this study	GEO: GSE122715
Pcgf1KO-Suz12 ChIP-Seq	this study	GEO: GSE122715
Pcgf24KO-H2AK119Ub1 ChIP-Seq	this study	GEO: GSE122715
Pcgf24KO-H3K27m3 ChIP-Seq	this study	GEO: GSE122715
Pcgf24KO-H3K36m3 ChIP-Seq	this study	GEO: GSE122715
Pcgf24KO-H3K4m3 ChIP-Seq	this study	GEO: GSE122715
Pcgf24KO-Pcgf1 ChIP-Seq	this study	GEO: GSE122715
Pcgf24KO-Pcgf2 ChIP-Seq	this study	GEO: GSE122715
Pcgf24KO-Pcgf3 ChIP-Seq	this study	GEO: GSE122715
Pcgf24KO-Pcgf6 ChIP-Seq	this study	GEO: GSE122715
Pcgf24KO-Ring1b ChIP-Seq	this study	GEO: GSE122715
Pcgf24KO-Suz12 ChIP-Seq	this study	GEO: GSE122715
Pcgf35KO-H2AK119Ub1 ChIP-Seq	this study	GEO: GSE122715
Pcgf35KO-H3K27m3 ChIP-Seq	this study	GEO: GSE122715
Pcgf35KO-H3K36m3 ChIP-Seq	this study	GEO: GSE122715
Pcgf35KO-H3K4m3 ChIP-Seq	this study	GEO: GSE122715
Pcgf35KO-Pcgf1 ChIP-Seq	this study	GEO: GSE122715
Pcgf35KO-Pcgf2 ChIP-Seq	this study	GEO: GSE122715
Pcgf35KO-Pcgf3 ChIP-Seq	this study	GEO: GSE122715
Pcgf35KO-Pcgf6 ChIP-Seq	this study	GEO: GSE122715
Pcgf35KO-Ring1b ChIP-Seq	this study	GEO: GSE122715
Pcgf35KO-Suz12 ChIP-Seq	this study	GEO: GSE122715
Pcgf6KO-H2AK119Ub1 ChIP-Seq	this study	GEO: GSE122715
Pcgf6KO-H3K27m3 ChIP-Seq	this study	GEO: GSE122715
Pcgf6KO-H3K36m3 ChIP-Seq	this study	GEO: GSE122715
Pcgf6KO-H3K4m3 ChIP-Seq	this study	GEO: GSE122715
Pcgf6KO-Pcgf1 ChIP-Seq	this study	GEO: GSE122715
Pcgf6KO-Pcgf2 ChIP-Seq	this study	GEO: GSE122715
Pcgf6KO-Pcgf3 ChIP-Seq	this study	GEO: GSE122715
Pcgf6KO-Pcgf6 ChIP-Seq	this study	GEO: GSE122715
Pcgf6KO-Ring1b ChIP-Seq	this study	GEO: GSE122715
Pcgf6KO-Suz12 ChIP-Seq	this study	GEO: GSE122715
RingdKO-Pcgf1 ChIP-Seq	this study	GEO: GSE122715
RingdKO-Pcgf2 ChIP-Seq	this study	GEO: GSE122715
RingdKO-Pcgf3 ChIP-Seq	this study	GEO: GSE122715
RingdKO-Pcgf6 ChIP-Seq	this study	GEO: GSE122715
RingFL-Pcgf1 ChIP-Seq	this study	GEO: GSE122715
RingFL-Pcgf2 ChIP-Seq	this study	GEO: GSE122715
RingFL-Pcgf3 ChIP-Seq	this study	GEO: GSE122715
RingFL-Pcgf6 ChIP-Seq	this study	GEO: GSE122715
shCtr-Pcgf3-rep1 ChIP-Seq	this study	GEO: GSE122715
shCtr-Pcgf3-rep2 ChIP-Seq	this study	GEO: GSE122715
shE2F6MgaDHLH-Pcgf6 ChIP-Seq	this study	GEO: GSE122715
shE2F6-Pcgf6 ChIP-Seq	this study	GEO: GSE122715
shMGA-Pcgf6 ChIP-Seq	this study	GEO: GSE122715
shUsf1-Pcgf3 ChIP-Seq	this study	GEO: GSE122715
shUsf2-Pcgf3 ChIP-Seq	this study	GEO: GSE122715
E14-Myc ChIP-Seq	this study	GEO: GSE122715
Input-E14-293T-DX ChIP-Seq	this study	GEO: GSE122715
Input-E14-293T ChIP-Seq	this study	GEO: GSE122715
Input-E14-293T-Gal4P6 ChIP-Se	this study	GEO: GSE122715
Input-Pcgf1KO-293T-DX ChIP-Se	this study	GEO: GSE122715
Input-Pcgf1KO-293T ChIP-Seq	this study	GEO: GSE122715
Input-Pcgf1KO-293T-Gal4P6 ChI	this study	GEO: GSE122715
Input-Pcgf24KO-293T-DX ChIP-S	this study	GEO: GSE122715
Input-Pcgf24KO-293T ChIP-Seq	this study	GEO: GSE122715
Input-Pcgf24KO-293T-Gal4P6 Ch	this study	GEO: GSE122715
Input-Pcgf35KO-293T-DX ChIP-S	this study	GEO: GSE122715
Input-Pcgf35KO-293T ChIP-Seq	this study	GEO: GSE122715
Input-Pcgf35KO-293T-Gal4P6 Ch	this study	GEO: GSE122715
Input-Pcgf6KO-293T-DX ChIP-Se	this study	GEO: GSE122715
Input-Pcgf6KO-293T ChIP-Seq	this study	GEO: GSE122715
Input-Pcgf6KO-293T-Gal4P6 ChI	this study	GEO: GSE122715
Pcgf1-E14-293T ChIP-Seq	this study	GEO: GSE122715
Pcgf1-Pcgf1KO-293T ChIP-Seq	this study	GEO: GSE122715
Pcgf1-Pcgf24KO-293T ChIP-Seq	this study	GEO: GSE122715
Pcgf1-Pcgf35KO-293T ChIP-Seq	this study	GEO: GSE122715
Pcgf1-Pcgf6KO-293T ChIP-Seq	this study	GEO: GSE122715
Pcgf2-E14-293T ChIP-Seq	this study	GEO: GSE122715
Pcgf2-Pcgf1KO-293T ChIP-Seq	this study	GEO: GSE122715
Pcgf2-Pcgf24KO-293T ChIP-Seq	this study	GEO: GSE122715
Pcgf2-Pcgf35KO-293T ChIP-Seq	this study	GEO: GSE122715
Pcgf2-Pcgf6KO-293T ChIP-Seq	this study	GEO: GSE122715
Pcgf3-E14-rep2 ChIP-Seq	this study	GEO: GSE122715
Pcgf3-Pcgf1KO-rep2 ChIP-Seq	this study	GEO: GSE122715
Pcgf3-Pcgf24KO-rep2 ChIP-Seq	this study	GEO: GSE122715
Pcgf3-Pcgf35KO-rep2 ChIP-Seq	this study	GEO: GSE122715
Pcgf3-Pcgf6KO-rep2 ChIP-Seq	this study	GEO: GSE122715
Pcgf6-E14-293T ChIP-Seq	this study	GEO: GSE122715
Pcgf6-Pcgf1KO-293T ChIP-Seq	this study	GEO: GSE122715
Pcgf6-Pcgf24KO-293T ChIP-Seq	this study	GEO: GSE122715
Pcgf6-Pcgf35KO-293T ChIP-Seq	this study	GEO: GSE122715
Pcgf6-Pcgf6KO-293T ChIP-Seq	this study	GEO: GSE122715
E14-rep1 RNA-Seq	this study	GEO: GSE122715
E14-rep2 RNA-Seq	this study	GEO: GSE122715
P124KO-rep1 RNA-Seq	this study	GEO: GSE122715
P124KO-rep2 RNA-Seq	this study	GEO: GSE122715
P1KO-rep1 RNA-Seq	this study	GEO: GSE122715
P1KO-rep2 RNA-Seq	this study	GEO: GSE122715
P24KO-rep1 RNA-Seq	this study	GEO: GSE122715
P24KO-rep2 RNA-Seq	this study	GEO: GSE122715
P356KO-rep1 RNA-Seq	this study	GEO: GSE122715
P356KO-rep2 RNA-Seq	this study	GEO: GSE122715
P35KO-rep1 RNA-Seq	this study	GEO: GSE122715
P35KO-rep2 RNA-Seq	this study	GEO: GSE122715
P6KO-rep1 RNA-Seq	this study	GEO: GSE122715
P6KO-rep2 RNA-Seq	this study	GEO: GSE122715

**Software and Algorithms**		

Bowtie v1.2.2	Langmead et al., 2009	http://bowtie-bio.sourceforge.net/index.shtml
PICARD	N/A	http://broadinstitute.github.io/picard
MACS2 v2.1.1	Zhang et al., 2008	https://github.com/taoliu/MACS
ChIPpeakAnno v3.15	Zhu et al., 2010	https://bioconductor.org/packages/release/bioc/html/ChIPpeakAnno.html
VennDiagram v1.6.20	Chen and Boutros, 2011	https://www.rdocumentation.org/packages/VennDiagram
ClusterProfiler	Yu et al., 2012	http://bioconductor.org/packages/release/bioc/html/clusterProfiler.html
HOMER	Heinz et al., 2010	http://homer.ucsd.edu/
DeepTools 2.0	Ramírez et al., 2016	https://deeptools.readthedocs.io/en/latest/
TopHat v2.1.1	Trapnell et al., 2009	https://ccb.jhu.edu/software/tophat/
HTseq-count v0.8.0	Anders et al., 2015	https://www.huber.embl.de/HTSeq
FIMO	Grant et al., 2011	http://meme-suite.org/doc/fimo.html

### Contact for Reagent and Resource Sharing

Further information and requests for resources and reagents should be directed to and will be fulfilled by the Lead Contact, Diego Pasini (diego.pasini@ieo.it).

### Experimental Model and Subject Details

#### Cell culture and cell manipulation

All ESC lines (E14 and derivatives) were grown in a 0.1% gelatin-coated dish in GMEM supplemented with 15% FBS (Euroclone), 2 mM L-glutamine, 100 U/mL penicillin, 0.1 mg/mL streptomycin, 0.1 mM non-essential amino acids, 1 mM sodium pyruvate, 50 μm β-mercaptoethanol (GIBCO), leukemia inhibitory factor, 3 μm GSK3β inhibitor (CHIR99021, STEMCELL Technologies), 1 μm MEK1/2 Inhibitor (PD0325901, STEMCELL Technologies).

For knockdown experiments, cells were transduced with 5 μg/mL polybrene and lentivirus particles delivering pLKO.1 vector expressing specific shRNAs for 16 hr; cells were then puromycin-selected (2 μg/mL) and grown for 72 hr prior harvesting.

To generate stable Pcgf KO cell lines, 10 μg pX459 2.0 plasmids (Addgene) encoding Cas9 and sgRNAs were transfected using Lipofectamine 2000 (Invitrogen), according to the manufacturer's instruction. Puromycin selection was performed for 30 hr at 2 μg/mL, 2000 cells were seeded into a 15-cm dish, and clones were isolated 10 days later. Clones were screened by PCR for genomic DNA, and Western Blot for protein lysates. PCR from positive clones were Sanger-sequenced to confirm genome editing. For double- or triple KO cell lines, single KO clones were subjected to genome editing following the same procedure.

The *Ring1A*^–/–^;*Ring1B*^fl/fl^;*Rosa26::CreERT2* conditional mESC line was described previously (Endoh et al., 2008).

Gal4-DBD-Pcgf inducible cell lines were obtained by transfecting the specific pCDNA4/TO-*Gal4*-*Pcgf* vector into 293TREx containing a stably integrated (Flp-In) 5 × Gal4TK-Luc-neo construct described previously (Pasini et al., 2010). Transfected cells were seeded at limiting dilutions, and isolated clones were screened by western blot for either Gal4 or Pcgf. The Gal4-DBD-*Pcgf* chimera was expressed using by 1 μg/mL doxycycline (Sigma) medium added for 48 hr before collecting for ChIP and luciferase reporter gene assay. For the latter, cells were lysed in passive lysis buffer (Promega) and then quantified with Bradford protein assay (Bio-Rad), and the luciferase assay (Promega) was performed on a GloMax instrument (Promega) according to the manufacturer’s instructions.

#### Plasmid Generation

Coding sequences for mouse PCGF proteins were amplified from E14 cDNA and cloned into pCR8/Gateway/TOPO/TA (Invitrogen) following the manufacturer’s protocol and sequenced verified. Different plasmids were subcloned in the desired compatible vectors by Gateway technology using LR recombinase (Invitrogen). pGEX-4T-*Pcgf* was generated to produce GST fusion proteins; and pCDNA4/TO-Gal4-*Pcgf*, for 293TREx Flp-In Gal4-*Pcgf*. For CRISPR/Cas9 genome editing, sgRNAs were cloned into the pX459 2.0 vector (Addgene). Details for sgRNAs and relative gene target are given in Table S1.

#### Protein Purification and Antibody Generation

GST-PCGF fusion proteins were produced in *E. coli* (BL21) according to standard protocols, purified from crude lysates through fast-flow glutathione Sepharose 4B resin (GE Healthcare), and eluted with elution buffer containing 10 mM reduced glutathione. Eluted proteins were dialyzed against PBS, verified by SDS-PAGE Coomassie staining, and used to immunize rabbits (carried out at Eurogentech). Antibodies from crude sera were immunoaffinity purified and tested for specificity.

#### Immunoblot, Immunoprecipitation, and Size-Exclusion Chromatography

Western blots were used to analyze lysates obtained with high-salt lysis buffer (20 mM Tris-HCl, pH 7.6, 300 mM NaCl, 10% glycerol, 0.2% [v/v] IGEPAL [Sigma-Aldrich]). For histone modification analyses, lysates were sonicated by Bioruptor (4 pulses at high intensity, 30 s on/off).

For immunoprecipitation experiments, nuclei were purified using nuclear preparation buffer (10 mM Tris, pH 7.6, 2 mM MgCl_2_, 0.34 M sucrose, 0.25% [v/v] IGEPAL), lysed in high-salt lysis buffer supplemented with EDTA-free cocktail proteases inhibitors (Roche), and incubated for 5 min at 37°C with 25 U/mL benzonase (Merck); 200–500 μg nuclear lysates were incubated with 2 μg of antibody for 2 hr at 4°C, and protein A magnetic beads (Invitrogen) were added for 45 min to recover immunoprecipitated complex. Beads were washed three times in high-salt buffer prior elution with loading sample buffer.

Size-exclusion chromatography of nuclear lysate was performed over a Superose 6 column (GE Healthcare) in 150 mM NaCl-salt buffer mounted on a AKTA chromatography system (GE Healthcare).

For GAL4 immunoprecipitation, 293TRex GAL4-PCGFs nuclear extracts (3 mg) were incubated with 14 μg of anti GAL4 antibody for 3 h at 4 °C and then 40 μl slurry of Sepharose Protein G beads were added for 2 h at 4 °C. IgG were used as negative control. Beads were washed with nuclear extraction buffer and immunocomplexes were eluted in Laemmli buffer and resolved on NuPAGE 4%–12% precast protein gels (Invitrogen).

Size-exclusion chromatography of nuclear lysate was performed over a Superose 6 column (GE Healthcare) in 150 mM NaCl-salt buffer mounted on a AKTA chromatography system (GE Healthcare).

#### Sample preparation and mass spectrometry analysis

Proteins from GAL4-PCGFs purification were separated by SDS–PAGE, using 4%–12% NuPAGE Novex Bis–Tris gels (Invitrogen) and NuPAGE MES SDS running buffer (Invitrogen) and then stained with Coomassie Blue using InstantBlue Comassie (Expedeon). Bands from gel were cut and digested with trypsin (Promega) and incubated for 16 h at 37°C for protein digestion. Then, peptide extraction was carried out and the resulting peptides mixture were combined, reduced in volume in a vacuum concentrator, desalted and concentrated using StageTip (Proxeon Biosystems) columns, washed with 30 μL of 0,1% Formic acid (FA) and finally eluted with 40 μL of 80% MeCN in 0,1% FA. The samples were concentrated in vacuum concentrator (Eppendorf concentrator 5301) for 5 min and peptides were dissolved in 7 μL of 0,1% FA. Approximately 5 μL of purified peptide mixture were analyzed on a LC–ESI–MS-MS Q Exactive HF hybrid quadrupole-Orbitrap mass spectrometer (Thermo Fisher Scientific). Full scan MS spectra were acquired in a range of m/z 300–1800.

#### Peptides and proteins identification by database searching

Raw data files were analyzed using the peptide search engine Andromeda integrated into the MaxQuant software environment (version 1.5.2.8) with the following parameters: uniprot_cp_hum_2017_01 as protein database, methionine oxidation, Protein N-term Acetylation as variable modifications and carbamidomethylation as fixed, peptide false discovery rate (FDR) 0.01, minimum peptides 2, at least 1 unique, minimum length peptide 6 amino acids. iBAQ intensity values as calculated by MaxQuant were used to estimate relative abundance of proteins. LFQ ratio values are listed in Table S4.

#### ChIP and ChIP-seq

ChIP assays were carried out as described previously (Ferrari et al., 2014). Briefly, 1% formaldehyde cross-linked chromatin (1 mg) was sonicated to an average size of 300–600 bp and incubated overnight at 4°C with 1–8 μg of the indicated antibodies. For ChIP-seq, column-purified DNA from a ChIP experiment was quantified using the Qubit dsDNA HS Assay Kit (Thermo Scientific, Q32854) and 10 ng DNA were processed at IEO NGS core unit employing an automated platform (Beckman Coulter) with the Illumina ChIP-seq sample prep kit (IP-102-1001) and multiplexing oligonucleotide kit (PE400-1001). DNA libraries were quality-checked and quantified on an automated sample processing workstation (Caliper Life Sciences) and used for cluster generation and sequencing by the HiSeq 2000 (Illumina) at 50 bp read length. For PCGF1 ChIP, chromatin was crosslinked for 50 min at RT with 2 mM EGS (Sigma) and then for 10 min with 1% formaldehyde. Quantitative ChIP experiments were performed relative to a reference exogenous genome (ChIP-Rx) (Orlando et al., 2014). For histone modifications, a total of 5% of *Drosophila* chromatin from S2 cell line was added to each ChIP reaction, while for PCGF1 and PCGF2 a total of 50% of human chromatin from 293T cell line was used and, for PCGF6 ChIP-Rx, a total of 50% of human chromatin from 293T cell line expressing GAL4-PCGF6 fusion protein was added to each PCGF6 ChIP reaction. Spiked chromatins were sheared at a size of 200–300 bp.

For re-ChIP assays, immunoprecipitated DNA after the first ChIP was eluted with 50 μL of 10 mM DTT for 30 min at 37°C and then diluted 10 × with ChIP buffer, allowing the second ChIP overnight.

#### ChIP-seq Analysis

Reads were aligned to the mouse reference genome mm9, or mm9 and dm6 for histone ChIP-Rx, or mm9 and hg38 for PCGF ChIP-Rx samples, using Bowtie v1.2.2 (Langmead et al., 2009) with default parameters without allowing for multi-mapping (–m 1). PCR duplicates were removed using PICARD (http://broadinstitute.github.io/picard/). Ambiguous reads mapping to both mm9 and dm6 or hg38 were discarded. Peaks were called using MACS2 v2.1.1 (Zhang et al., 2008) with parameters -g mm–nomodel -p 1e-10 –B. Due to a higher IP efficiency in PCGF1 rep2, we used a more stringent p value cutoff (10e-30) to make the data comparable to PCGF1 rep1. To avoid false positives, peaks were discarded if they were in both PCGF wild-type samples and the respective PCGF knockout samples. Then, peaks from biological replicates were merged together. A list containing the final PCGF peaks used in the analyses can be found in Table S2. Genomic peak annotation was performed with the R package ChIPpeakAnno v3.15 (Zhu et al., 2010), considering the region ± 2.5 kb around the TSS as the promoter. Scanning of PCGF6 targets for E-box and E2F sites downloaded from JASPAR (Khan et al., 2018) was done using FIMO (Grant et al., 2011) with default parameters. All downstream analyses were performed considering peaks overlapping with promoter regions, unless otherwise specified. Peak lists were then transformed to gene target lists (Table S3), and overlaps were performed using the R package VennDiagram v1.6.20 (Chen and Boutros, 2011). Gene ontology analyses of PCGF targets were performed using the Bioconductor package clusterProfiler (Yu et al., 2012) setting as threshold an adjusted p value and q-value of 0.01. Motif discovery was performed using HOMER (Heinz et al., 2010) with default parameters using as input the regions ± 25 bp around peak summits reported by MACS2.

For heatmap and intensity plot representation of ChIP-seq signal, BigWig files with input signal subtracted were generated using the function bamCompare from deepTools 2.0 (Ramírez et al., 2016) with parameters–ratio subtract –bs 50–extendReads 200. To normalize for differences in sample library size, a scaling factor for each sample was calculated as (1/total mapped reads)^∗^1000000 and was applied during BigWig file generation with the parameter –scaleFactors from bamCompare. For ChIP-Rx samples the scaling factor was calculated as described in (Orlando et al., 2014). Heatmaps were performed using the functions computeMatrix followed by plotHeatmap from deepTools excluding blacklisted regions by ENCODE (Consortium, 2012) (Consortium, 2012). To homogenize the scale of all heatmaps, boxplots and intensity plots, signal intensity was scaled to 0–1 (represented by min-max in the boxplot figures) by applying the formula 1/(P98 – P5) to all matrices generated by computeMatrix. In order to minimize any difference in the IP and library preparation efficiencies between the two batches of biological replicates, the matrices generated from replicates 1 and replicates 2 were averaged and plotted as a single heatmap.

#### RNA-seq

RNA-seq was performed with minor modifications according to the SMART-seq2 protocol (Picelli et al., 2014). Briefly, poly-A containing mRNA molecules from 2 μg of total extracted RNA were copied into first-strand cDNA by reverse transcription and template-switching using oligo(dT) primers and an LNA-containing template-switching oligo (TSO); resulting cDNA was pre-amplified, purified, and tagmented with in-house produced Tn5 transposase. cDNA fragments generated after tagmentation were gap-repaired, amplified by PCR, and cleaned to obtain the final cDNA library.

#### RNA-seq Analysis

Reads were aligned to the mouse reference genome mm9 using TopHat v2.1.1 (Trapnell et al., 2009) with parameters–no-coverage-search and–library-type fr-unstranded. PCR duplicates were removed using PICARD (http://broadinstitute.github.io/picard/). Gene counts were calculated using HTseq-count v0.8.0 (Anders et al., 2015) with parameters–stranded = no–mode = intersection-nonempty using RefSeq mm9 annotation downloaded from UCSC. Differential expression analyses were performed using the R package DESeq2 v1.20 (Love et al., 2014) using default parameters. Genes with an absolute log2 fold change of 2 and FDR < 0.05 were considered as differentially expressed (Table S5). Gene ontology analysis of differentially expressed genes was performed using the Bioconductor package clusterProfiler (Yu et al., 2012) with default parameters. Full results of gene ontology analyses are provided in Table S6.

#### Quantitative Real-Time PCR

Total RNA was extracted from cells using the Quick-RNA kit (Zymo) according to manufacturer’s protocol. RNA was used to generate cDNA by reverse transcriptase PCR using the M-MuLV Reverse Transcriptase (Promega). Relative mRNA expression levels were determined using the Go-Taq SYBR Green (Promega) on a Bio-Rad Real-Time PCR System with selected primer pairs. Expression levels were normalized to *Gapdh*, used as a control housekeeping gene, and computed as described previously (Ferrari et al., 2014).

#### Data availability

ChIP-seq and RNA-seq datasets are available at GEO database this accession number: GSE122715

## Supplemental Information

Document S1. Figures S1– S10

Table S1. shRNA and sgDNA Sequences, Related to STAR Methods List of oligo sequences used for RNAi and CRISPR/cas9 manipulations of mESCs.

Table S2. PCGF-Enriched Genomic Regions, Related to Figure 1 List of peaks called for the different PCGF ChIP in WT mESCs.

Table S3. PCGF Target Genes, Related to Figure 1 List of target promoters bound by each PCGF protein in WT mESCs.

Table S4. Gal4-PCGF Fusion Protein MS Results, Related to Figure 2 Values of the LFQ ratios of the different PRC1.1–PRC1.6 components obtained by MS/MS analyses in the Gal4-PCGF1-6 fusion immuno-purifications (anti-Gal4) from 293TRex cells.

Table S5. Gene Expression in PCGF KO ESC, Related to Figure 4 Expression values of all RefSeq genes in the different PCGF KO mESC lines.

Table S6. Gene Ontology of Differentially Expressed Genes, Related to Figure 4 List of significant GO terms enriched in differentially expressed genes in the different PCGF KO mESC lines.

Document S2. Article plus Supplemental Information
