# Supplementary material for: Functional Landscape of PCGF Proteins Reveals Both RING1A/B-Dependent-and RING1A/B-Independent-Specific Activities
Source: Mol Cell. 2019 Jun 6;74(5):1037–1052.e7. doi: 10.1016/j.molcel.2019.04.002 (PMC6561742; doi:10.1016/j.molcel.2019.04.002)
Supplement: Document S1. Figures S1– S10 [file mmc1.pdf]

Molecular Cell, Volume *74*

Supplemental Information

Functional Landscape of PCGF Proteins Reveals Both RING1A/B-Dependent- and RING1A/B-Independent-Specific Activities

Andrea Scelfo, Daniel Fernández-Pérez, Simone Tamburri, Marika Zanotti, Elisa Lavarone, Monica Soldi, Tiziana Bonaldi, Karin Johanna Ferrari, and Diego Pasini

Figure S1

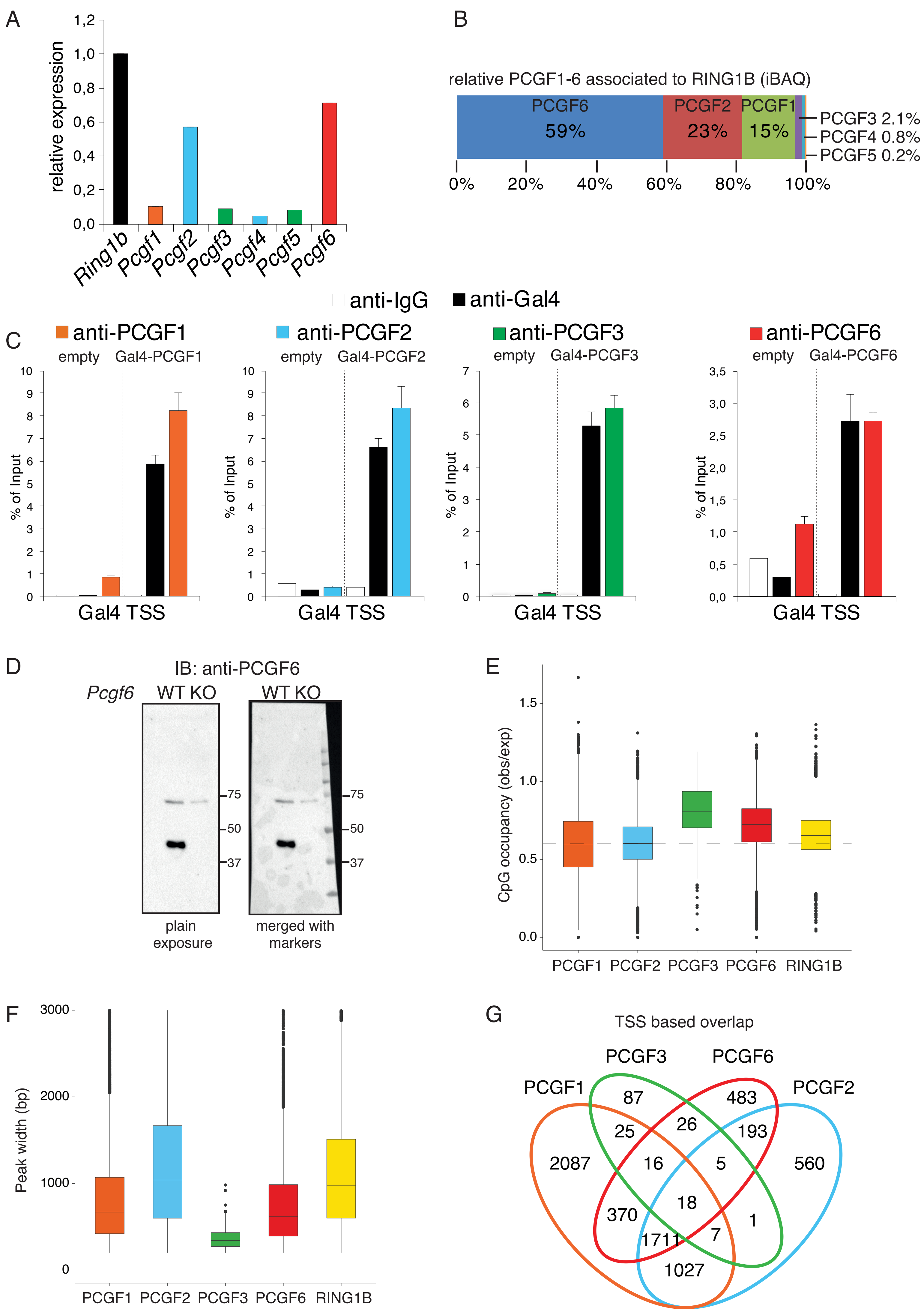
A
relative expression
Ring1b
Pcgf1
Pcgf2
Pcgf3
Pcgf4
Pcgf5
Pcgf6
B
relative PCGF1-6 associated to RING1B (iBAQ)
PCGF6 59%
PCGF2 23%
PCGF1 15%
PCGF3 2.1%
PCGF4 0.8%
PCGF5 0.2%
anti-IgG
anti-Gal4
C
anti-PCGF1
anti-PCGF2
anti-PCGF3
anti-PCGF6
empty
Gal4-PCGF1
Gal4-PCGF2
Gal4-PCGF3
Gal4-PCGF6
% of Input
Gal4 TSS
D
IB: anti-PCGF6
Pcgf6
WT KO
plain exposure
merged with markers
E
CpG occupancy (obs/exp)
PCGF1 PCGF2 PCGF3 PCGF6 RING1B
F
Peak width (bp)
G
TSS based overlap
PCGF1
PCGF3
PCGF6
PCGF2

Figure S2

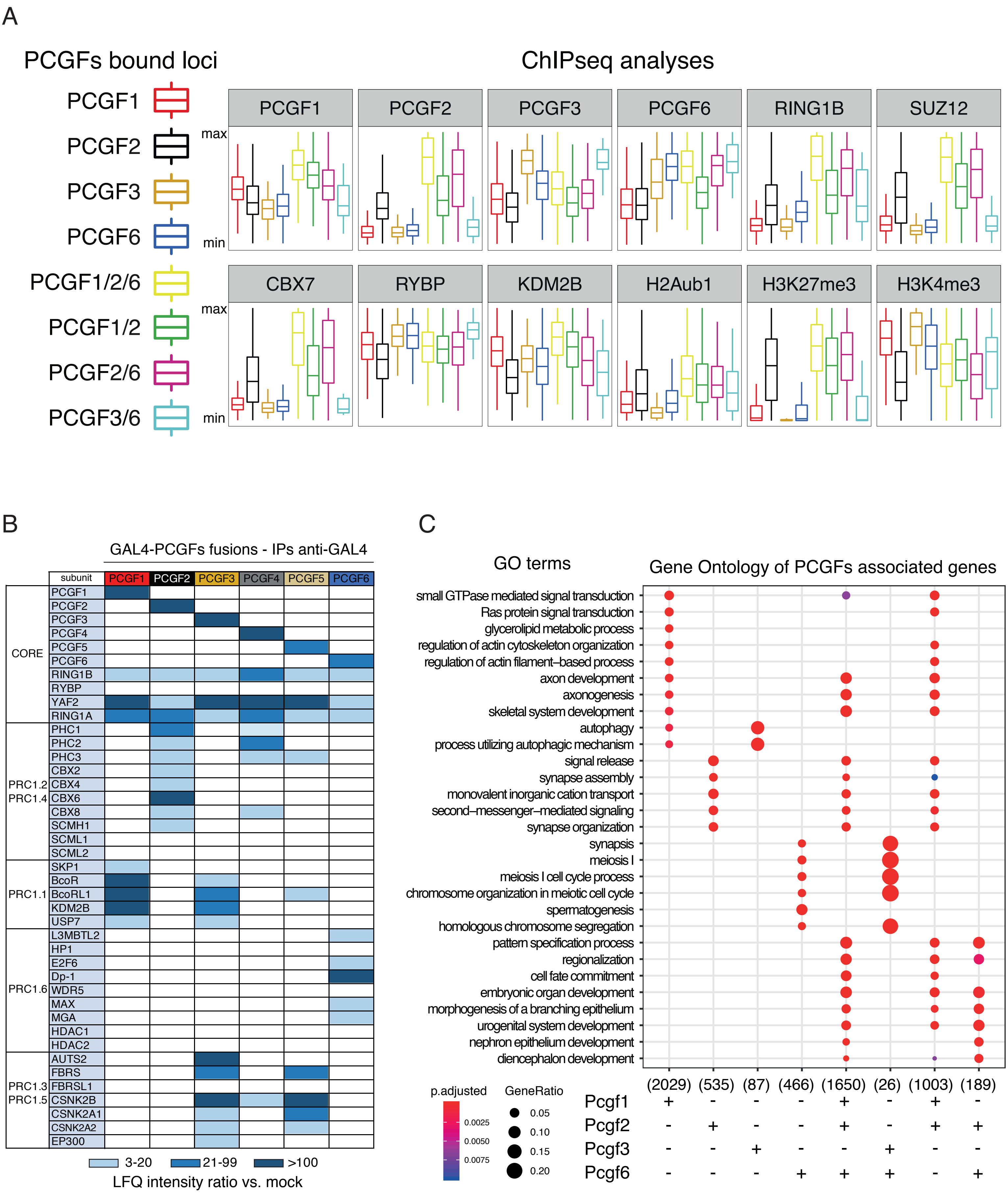
A
PCGFs bound loci
PCGF1
PCGF2
PCGF3
PCGF6
PCGF1/2/6
PCGF1/2
PCGF2/6
PCGF3/6
ChIPseq analyses
max
min
PCGF1
PCGF2
PCGF3
PCGF6
RING1B
SUZ12
CBX7
RYBP
KDM2B
H2Aub1
H3K27me3
H3K4me3
B
GAL4-PCGFs fusions - IPs anti-GAL4
subunit
PCGF1
PCGF2
PCGF3
PCGF4
PCGF5
PCGF6
CORE
PCGF1
PCGF2
PCGF3
PCGF4
PCGF5
PCGF6
RING1B
RYBP
YAF2
RING1A
PRC1.2
PRC1.4
PHC1
PHC2
PHC3
CBX2
CBX4
CBX6
CBX8
SCMH1
SCML1
SCML2
PRC1.1
SKP1
BcoR
BcoRL1
KDM2B
USP7
PRC1.6
L3MBTL2
HP1
E2F6
Dp-1
WDR5
MAX
MGA
HDAC1
HDAC2
PRC1.3
PRC1.5
AUTS2
FBRS
FBRSL1
CSNK2B
CSNK2A1
CSNK2A2
EP300
3-20
21-99
>100
LFQ intensity ratio vs. mock
C
GO terms
Gene Ontology of PCGFs associated genes
small GTPase mediated signal transduction
Ras protein signal transduction
glycerolipid metabolic process
regulation of actin cytoskeleton organization
regulation of actin filament–based process
axon development
axonogenesis
skeletal system development
autophagy
process utilizing autophagic mechanism
signal release
synapse assembly
monovalent inorganic cation transport
second–messenger–mediated signaling
synapse organization
synapsis
meiosis I
meiosis I cell cycle process
chromosome organization in meiotic cell cycle
spermatogenesis
homologous chromosome segregation
pattern specification process
regionalization
cell fate commitment
embryonic organ development
morphogenesis of a branching epithelium
urogenital system development
nephron epithelium development
diencephalon development
(2029) (535) (87) (466) (1650) (26) (1003) (189)
p.adjusted
0.0025
0.0050
0.0075
GeneRatio
0.05
0.10
0.15
0.20
Pcgf1 + - - - + - + -
Pcgf2 - + - - + - + +
Pcgf3 - - + - - + - -
Pcgf6 - - - + + + - +

Figure S3

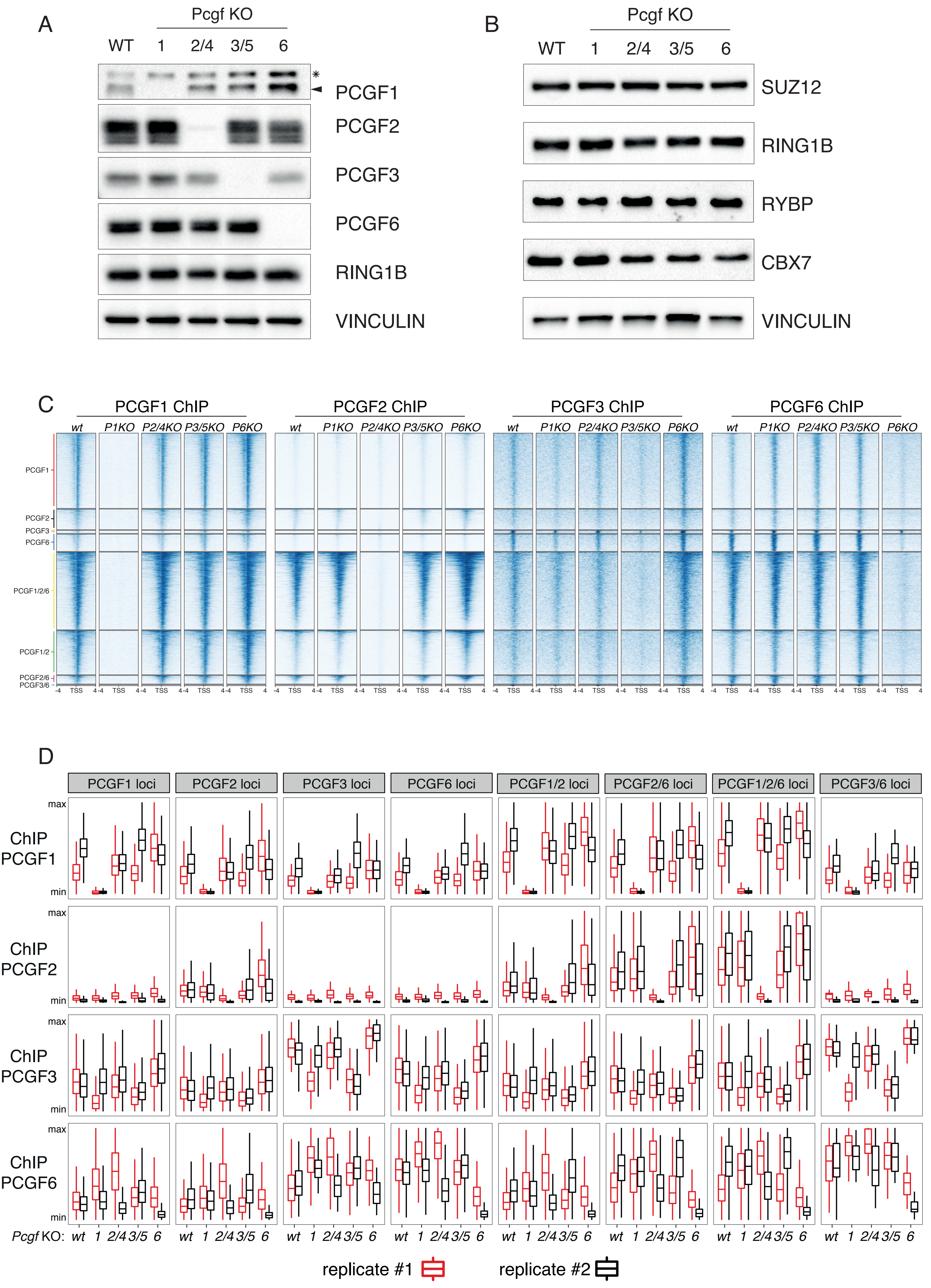
A
Pcgf KO
WT
1
2/4
3/5
6
*
PCGF1
PCGF2
PCGF3
PCGF6
RING1B
VINCULIN
B
Pcgf KO
WT
1
2/4
3/5
6
SUZ12
RING1B
RYBP
CBX7
VINCULIN
C
PCGF1 ChIP
PCGF2 ChIP
PCGF3 ChIP
PCGF6 ChIP
wt
P1KO
P2/4KO
P3/5KO
P6KO
PCGF1
PCGF2
PCGF3
PCGF6
PCGF1/2/6
PCGF1/2
PCGF2/6
PCGF3/6
-4
TSS
4
D
PCGF1 loci
PCGF2 loci
PCGF3 loci
PCGF6 loci
PCGF1/2 loci
PCGF2/6 loci
PCGF1/2/6 loci
PCGF3/6 loci
max
min
ChIP PCGF1
ChIP PCGF2
ChIP PCGF3
ChIP PCGF6
Pcgf KO: wt 1 2/4 3/5 6
replicate #1
replicate #2

Figure S4

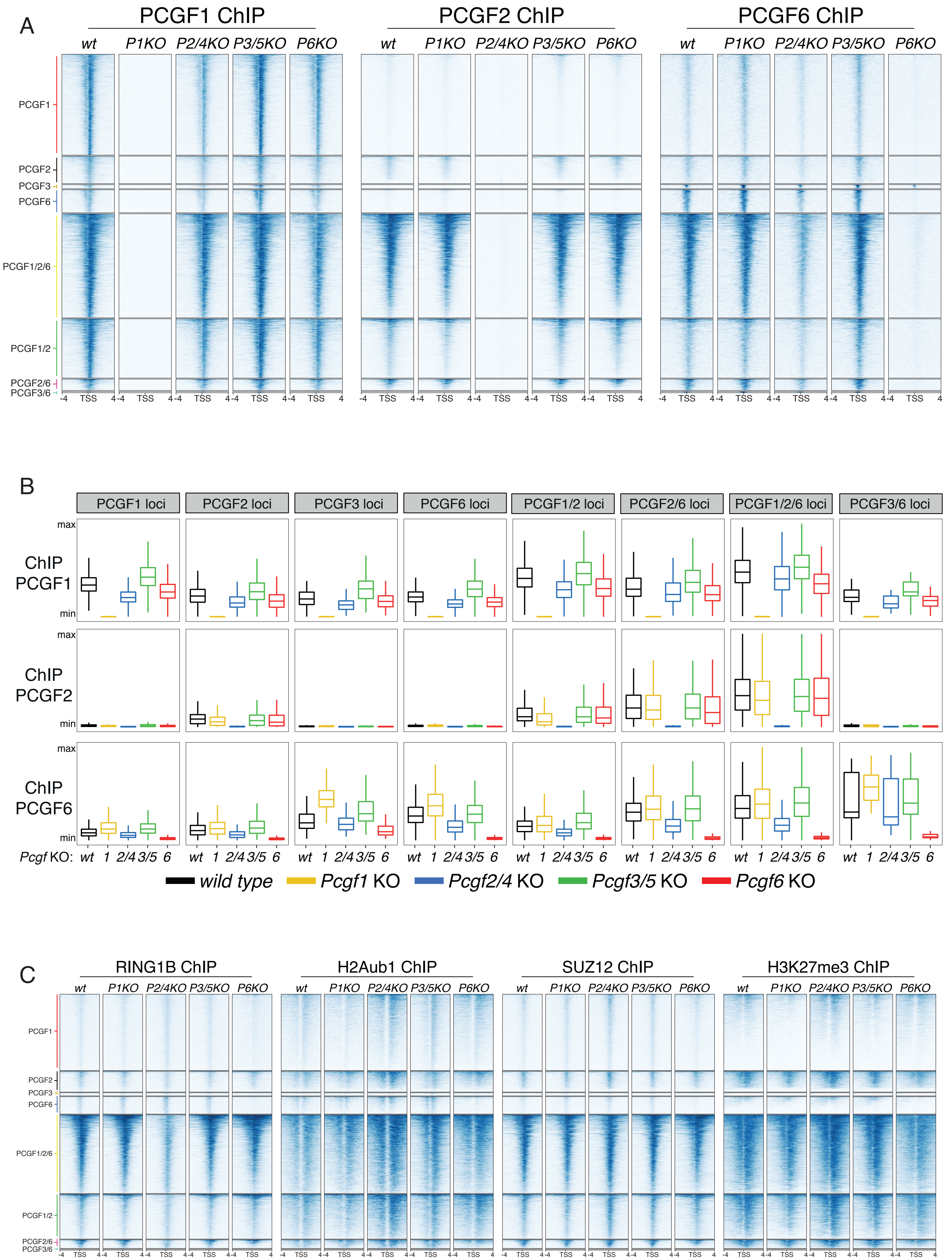
A
PCGF1 ChIP
PCGF2 ChIP
PCGF6 ChIP
wt
P1KO
P2/4KO
P3/5KO
P6KO
PCGF1
PCGF2
PCGF3
PCGF6
PCGF1/2/6
PCGF1/2
PCGF2/6
PCGF3/6
-4
TSS
4
B
PCGF1 loci
PCGF2 loci
PCGF3 loci
PCGF6 loci
PCGF1/2 loci
PCGF2/6 loci
PCGF1/2/6 loci
PCGF3/6 loci
max
min
ChIP PCGF1
ChIP PCGF2
ChIP PCGF6
Pcgf KO: wt 1 2/4 3/5 6
wild type
Pcgf1 KO
Pcgf2/4 KO
Pcgf3/5 KO
Pcgf6 KO
C
RING1B ChIP
H2Aub1 ChIP
SUZ12 ChIP
H3K27me3 ChIP
wt
P1KO
P2/4KO
P3/5KO
P6KO
PCGF1
PCGF2
PCGF3
PCGF6
PCGF1/2/6
PCGF1/2
PCGF2/6
PCGF3/6
-4
TSS
4

Figure S5

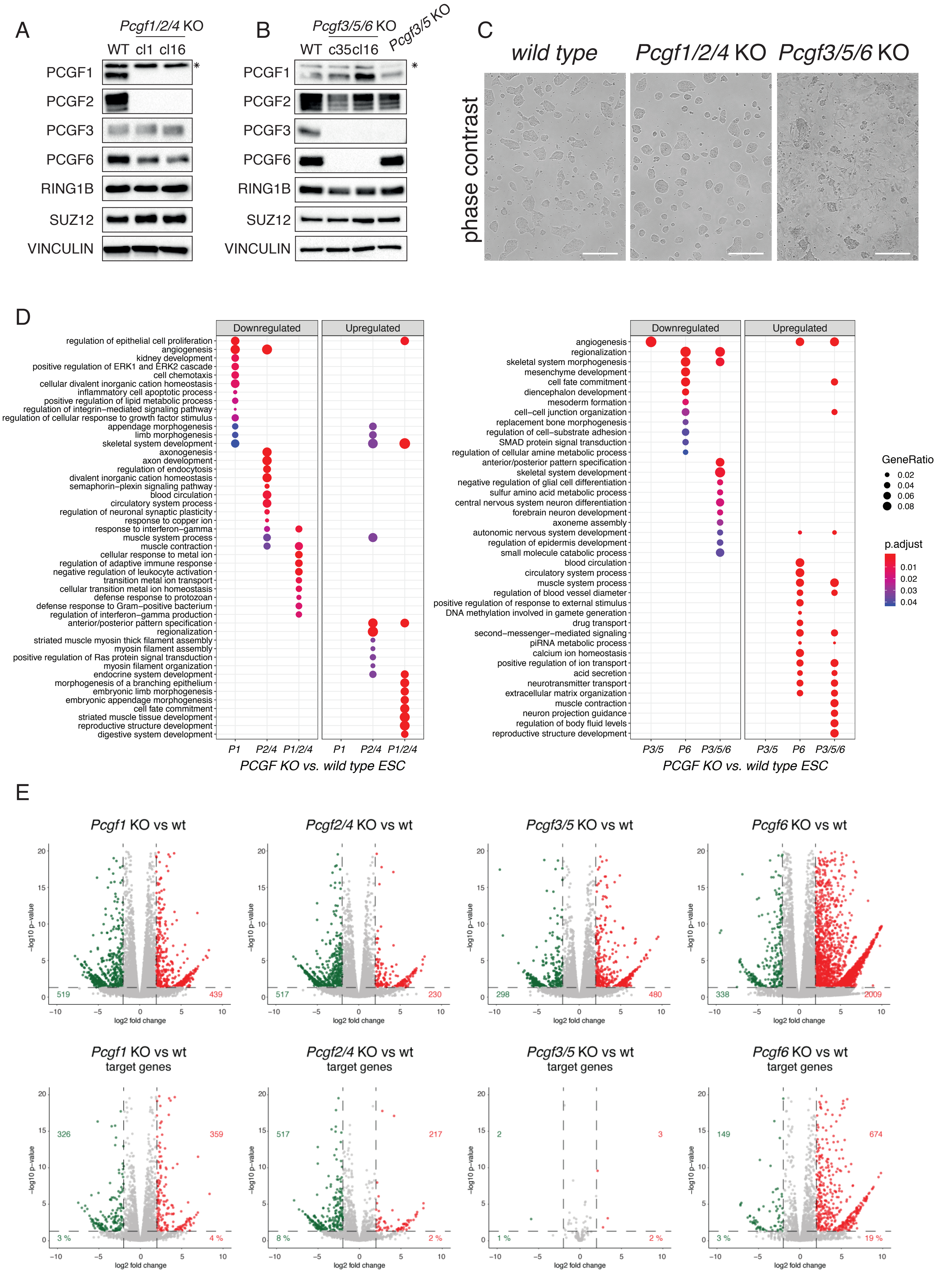

A
Pcgf1/2/4 KO
WT cl1 cl16
PCGF1
PCGF2
PCGF3
PCGF6
RING1B
SUZ12
VINCULIN
*
B
Pcgf3/5/6 KO
Pcgf3/5 KO
WT c35cl16
PCGF1
PCGF2
PCGF3
PCGF6
RING1B
SUZ12
VINCULIN
*
C
wild type
Pcgf1/2/4 KO
Pcgf3/5/6 KO
phase contrast
D
Downregulated
Upregulated
regulation of epithelial cell proliferation
angiogenesis
kidney development
positive regulation of ERK1 and ERK2 cascade
cell chemotaxis
cellular divalent inorganic cation homeostasis
inflammatory cell apoptotic process
positive regulation of lipid metabolic process
regulation of integrin−mediated signaling pathway
regulation of cellular response to growth factor stimulus
appendage morphogenesis
limb morphogenesis
skeletal system development
axonogenesis
axon development
regulation of endocytosis
divalent inorganic cation homeostasis
semaphorin−plexin signaling pathway
blood circulation
circulatory system process
regulation of neuronal synaptic plasticity
response to copper ion
response to interferon−gamma
muscle system process
muscle contraction
cellular response to metal ion
regulation of adaptive immune response
negative regulation of leukocyte activation
transition metal ion transport
cellular transition metal ion homeostasis
defense response to protozoan
defense response to Gram−positive bacterium
regulation of interferon−gamma production
anterior/posterior pattern specification
regionalization
striated muscle myosin thick filament assembly
myosin filament assembly
positive regulation of Ras protein signal transduction
myosin filament organization
endocrine system development
morphogenesis of a branching epithelium
embryonic limb morphogenesis
embryonic appendage morphogenesis
cell fate commitment
striated muscle tissue development
reproductive structure development
digestive system development
P1 P2/4 P1/2/4
P1 P2/4 P1/2/4
PCGF KO vs. wild type ESC
Downregulated
Upregulated
angiogenesis
regionalization
skeletal system morphogenesis
mesenchyme development
cell fate commitment
diencephalon development
mesoderm formation
cell−cell junction organization
replacement bone morphogenesis
regulation of cell−substrate adhesion
SMAD protein signal transduction
regulation of cellular amine metabolic process
anterior/posterior pattern specification
skeletal system development
negative regulation of glial cell differentiation
sulfur amino acid metabolic process
central nervous system neuron differentiation
forebrain neuron development
axoneme assembly
autonomic nervous system development
regulation of epidermis development
small molecule catabolic process
blood circulation
circulatory system process
muscle system process
regulation of blood vessel diameter
positive regulation of response to external stimulus
DNA methylation involved in gamete generation
drug transport
second−messenger−mediated signaling
piRNA metabolic process
calcium ion homeostasis
positive regulation of ion transport
acid secretion
neurotransmitter transport
extracellular matrix organization
muscle contraction
neuron projection guidance
regulation of body fluid levels
reproductive structure development
P3/5 P6 P3/5/6
P3/5 P6 P3/5/6
PCGF KO vs. wild type ESC
GeneRatio
0.02
0.04
0.06
0.08
p.adjust
0.01
0.02
0.03
0.04
E
Pcgf1 KO vs wt
519
439
Pcgf2/4 KO vs wt
517
230
Pcgf3/5 KO vs wt
298
480
Pcgf6 KO vs wt
338
2009
−log10 p−value
log2 fold change
Pcgf1 KO vs wt
target genes
326
359
3 %
4 %
Pcgf2/4 KO vs wt
target genes
517
217
8 %
2 %
Pcgf3/5 KO vs wt
target genes
2
3
1 %
2 %
Pcgf6 KO vs wt
target genes
149
674
3 %
19 %

Figure S6

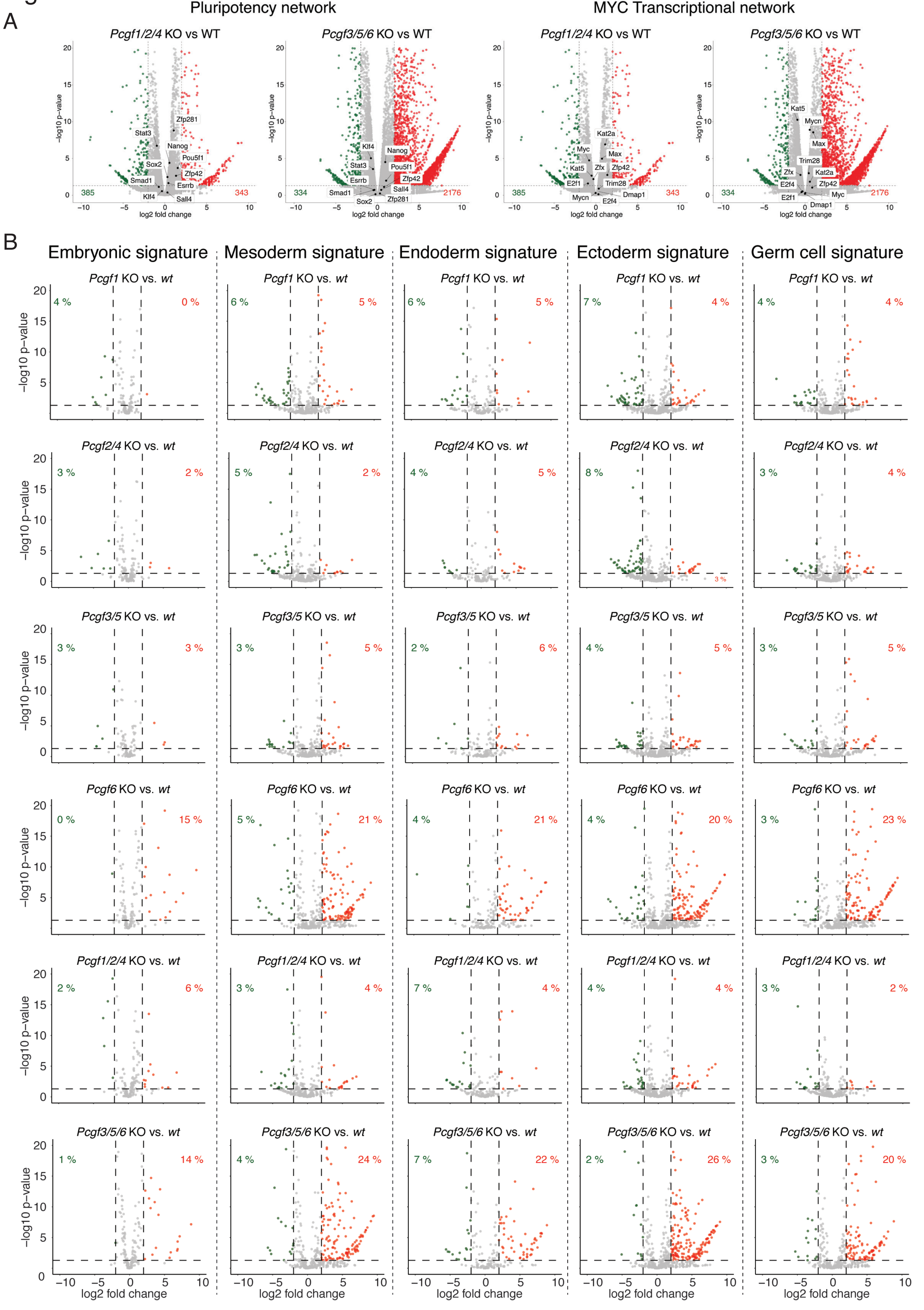
A
Pluripotency network
MYC Transcriptional network
Pcgf1/2/4 KO vs WT
Pcgf3/5/6 KO vs WT
Pcgf1/2/4 KO vs WT
Pcgf3/5/6 KO vs WT
-log10 p-value
log2 fold change
385
343
334
2176
385
343
334
2176
Zfp281
Stat3
Nanog
Pou5f1
Sox2
Zfp42
Smad1
Esrrb
Klf4
Sall4
Klf4
Nanog
Stat3
Pou5f1
Esrrb
Zfp42
Smad1
Sall4
Sox2
Zfp281
Kat2a
Myc
Max
Kat5
Zfx
Zfp42
E2f1
Trim28
Mycn
E2f4
Dmap1
Kat5
Mycn
Max
Trim28
Zfx
Kat2a
E2f4
Zfp42
E2f1
Myc
Dmap1
B
Embryonic signature
Mesoderm signature
Endoderm signature
Ectoderm signature
Germ cell signature
Pcgf1 KO vs. wt
Pcgf2/4 KO vs. wt
Pcgf3/5 KO vs. wt
Pcgf6 KO vs. wt
Pcgf1/2/4 KO vs. wt
Pcgf3/5/6 KO vs. wt
−log10 p−value
log2 fold change

Figure S7

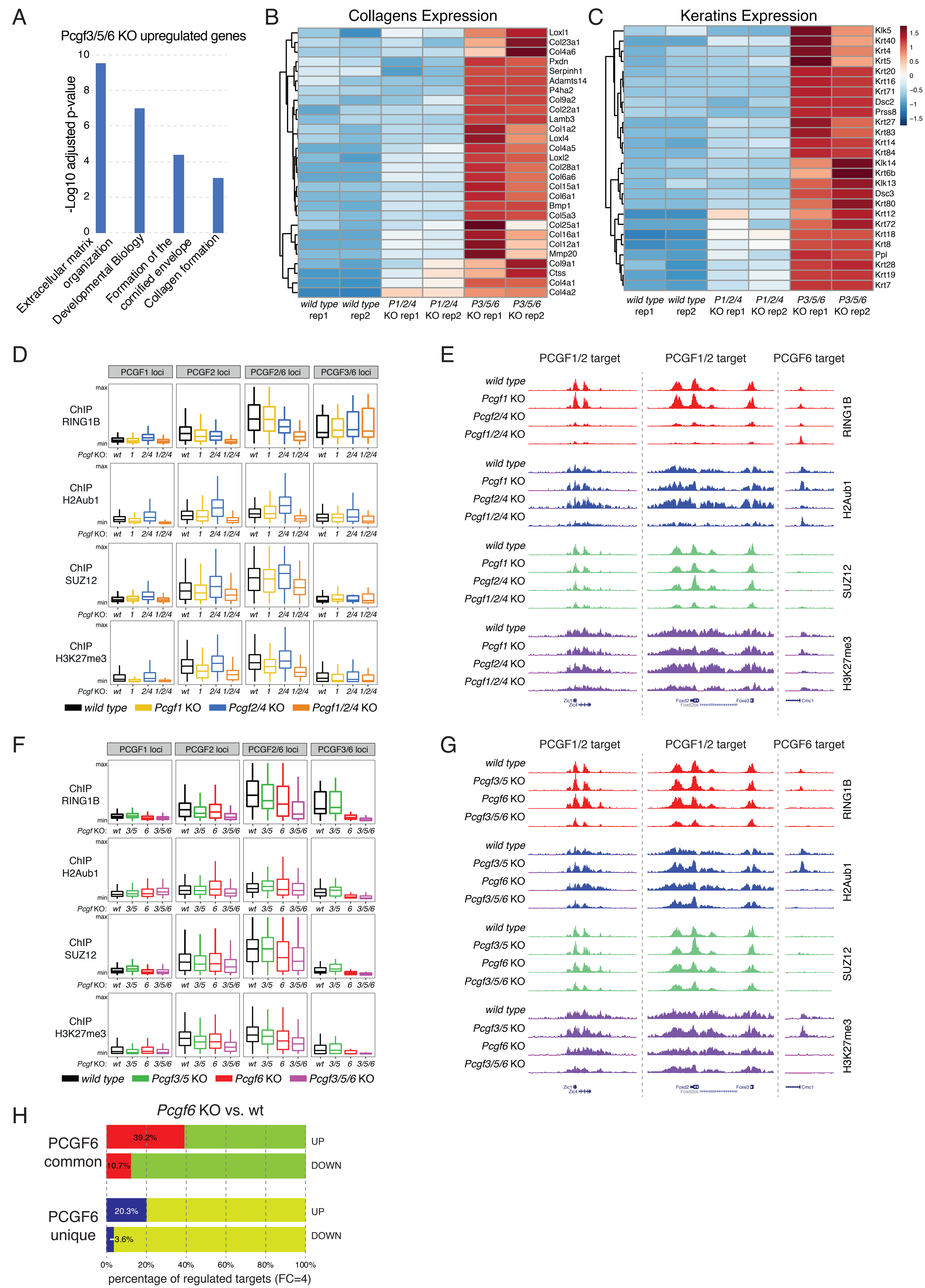
A
Pcgf3/5/6 KO upregulated genes
-Log10 adjusted p-value
Extracellular matrix organization
Developmental Biology
Formation of the cornified envelope
Collagen formation
B
Collagens Expression
Loxl1
Col23a1
Col4a6
Pxdn
Serpinh1
Adamts14
P4ha2
Col9a2
Col22a1
Lamb3
Col1a2
Loxl4
Col4a5
Loxl2
Col28a1
Col6a6
Col15a1
Col6a1
Bmp1
Col5a3
Col25a1
Col16a1
Col12a1
Mmp20
Col9a1
Ctss
Col4a1
Col4a2
wild type rep1
wild type rep2
P1/2/4 KO rep1
P1/2/4 KO rep2
P3/5/6 KO rep1
P3/5/6 KO rep2
C
Keratins Expression
Klk5
Krt40
Krt4
Krt5
Krt20
Krt16
Krt71
Dsc2
Prss8
Krt27
Krt83
Krt14
Krt84
Klk14
Krt6b
Klk13
Dsc3
Krt80
Krt12
Krt72
Krt18
Krt8
Ppl
Krt28
Krt19
Krt7
D
PCGF1 loci
PCGF2 loci
PCGF2/6 loci
PCGF3/6 loci
ChIP RING1B
ChIP H2Aub1
ChIP SUZ12
ChIP H3K27me3
Pcgf KO: wt 1 2/4 1/2/4
wild type
Pcgf1 KO
Pcgf2/4 KO
Pcgf1/2/4 KO
E
PCGF1/2 target
PCGF1/2 target
PCGF6 target
RING1B
H2Aub1
SUZ12
H3K27me3
F
Pcgf KO: wt 3/5 6 3/5/6
Pcgf3/5 KO
Pcgf6 KO
Pcgf3/5/6 KO
G
H
Pcgf6 KO vs. wt
PCGF6 common
PCGF6 unique
UP
DOWN
39.2%
10.7%
20.3%
3.6%
0%
20%
40%
60%
80%
100%
percentage of regulated targets (FC=4)

Figure S8

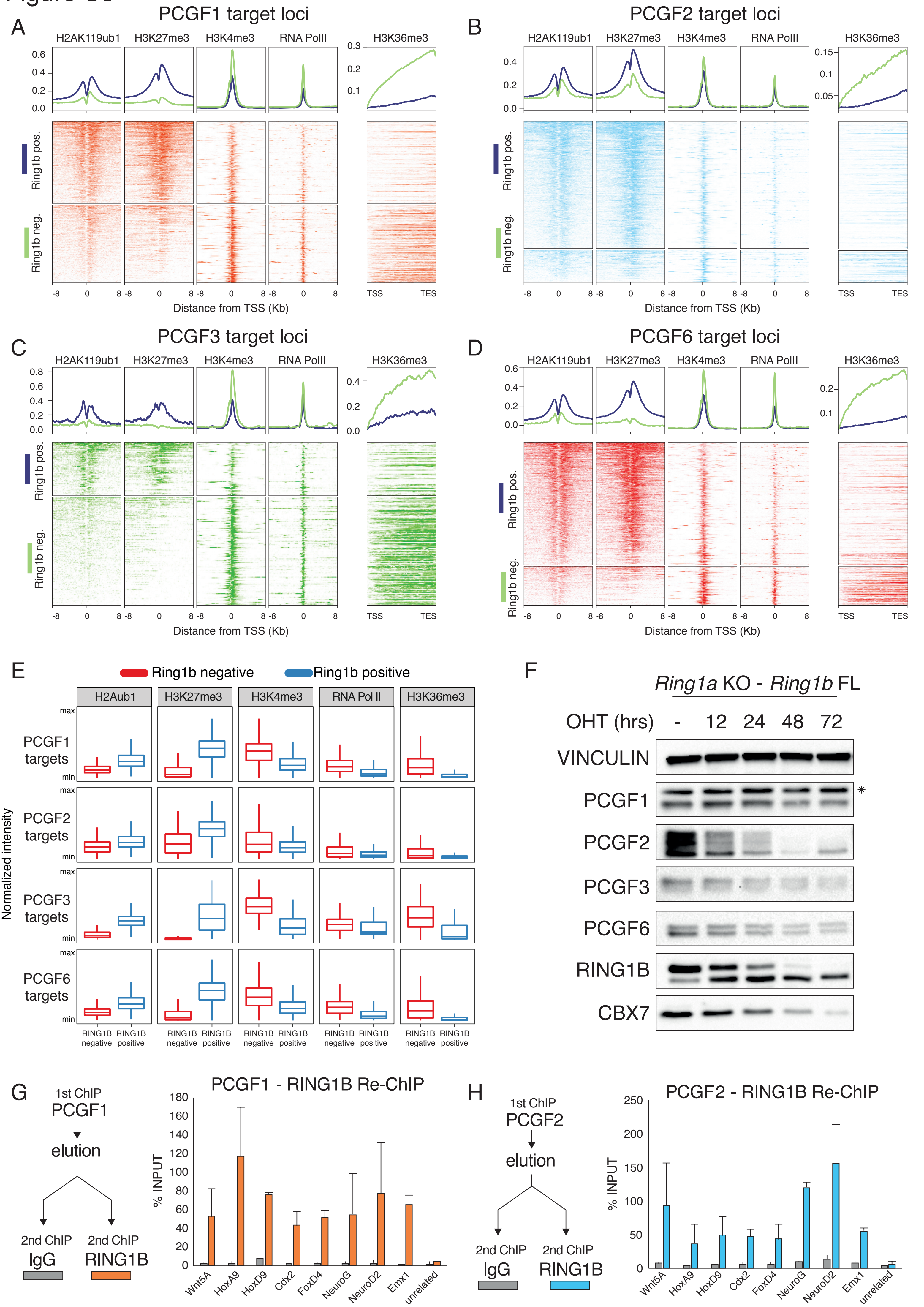
A
PCGF1 target loci
H2AK119ub1
H3K27me3
H3K4me3
RNA PolII
H3K36me3
Ring1b pos.
Ring1b neg.
TSS
TES
Distance from TSS (Kb)
B
PCGF2 target loci
C
PCGF3 target loci
D
PCGF6 target loci
E
Ring1b negative
Ring1b positive
H2Aub1
H3K27me3
H3K4me3
RNA Pol II
H3K36me3
PCGF1 targets
PCGF2 targets
PCGF3 targets
PCGF6 targets
Normalized intensity
max
min
RING1B negative
RING1B positive
F
Ring1a KO - Ring1b FL
OHT (hrs) - 12 24 48 72
VINCULIN
PCGF1
PCGF2
PCGF3
PCGF6
RING1B
CBX7
G
1st ChIP
PCGF1
elution
2nd ChIP
IgG
2nd ChIP
RING1B
PCGF1 - RING1B Re-ChIP
% INPUT
Wnt5A
HoxA9
HoxD9
Cdx2
FoxD4
NeuroG
NeuroD2
Emx1
unrelated
H
1st ChIP
PCGF2
elution
2nd ChIP
IgG
2nd ChIP
RING1B
PCGF2 - RING1B Re-ChIP
% INPUT

Figure S9

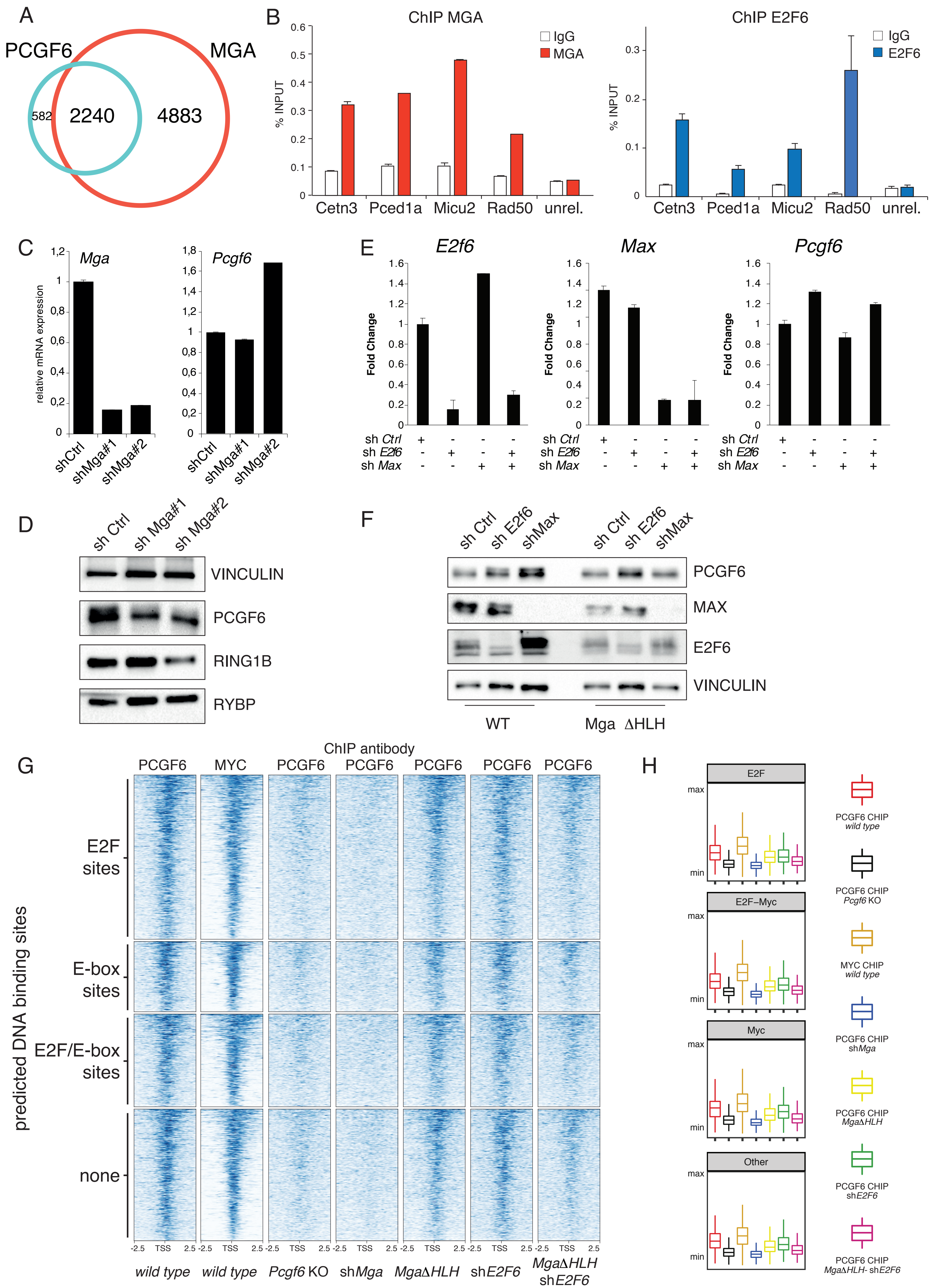
A
PCGF6
MGA
582
2240
4883
B
ChIP MGA
% INPUT
IgG
MGA
Cetn3
Pced1a
Micu2
Rad50
unrel.
ChIP E2F6
IgG
E2F6
C
Mga
Pcgf6
relative mRNA expression
shCtrl
shMga#1
shMga#2
E
E2f6
Max
Pcgf6
Fold Change
sh Ctrl
sh E2f6
sh Max
D
sh Ctrl
sh Mga#1
sh Mga#2
VINCULIN
PCGF6
RING1B
RYBP
F
sh Ctrl
sh E2f6
shMax
PCGF6
MAX
E2F6
VINCULIN
WT
Mga ΔHLH
G
ChIP antibody
PCGF6
MYC
predicted DNA binding sites
E2F sites
E-box sites
E2F/E-box sites
none
-2.5
TSS
2.5
wild type
Pcgf6 KO
shMga
MgaΔHLH
shE2F6
MgaΔHLH shE2F6
H
E2F
E2F–Myc
Myc
Other
max
min
PCGF6 CHIP wild type
PCGF6 CHIP Pcgf6 KO
MYC CHIP wild type
PCGF6 CHIP shMga
PCGF6 CHIP MgaΔHLH
PCGF6 CHIP shE2F6
PCGF6 CHIP MgaΔHLH- shE2F6

Figure S10

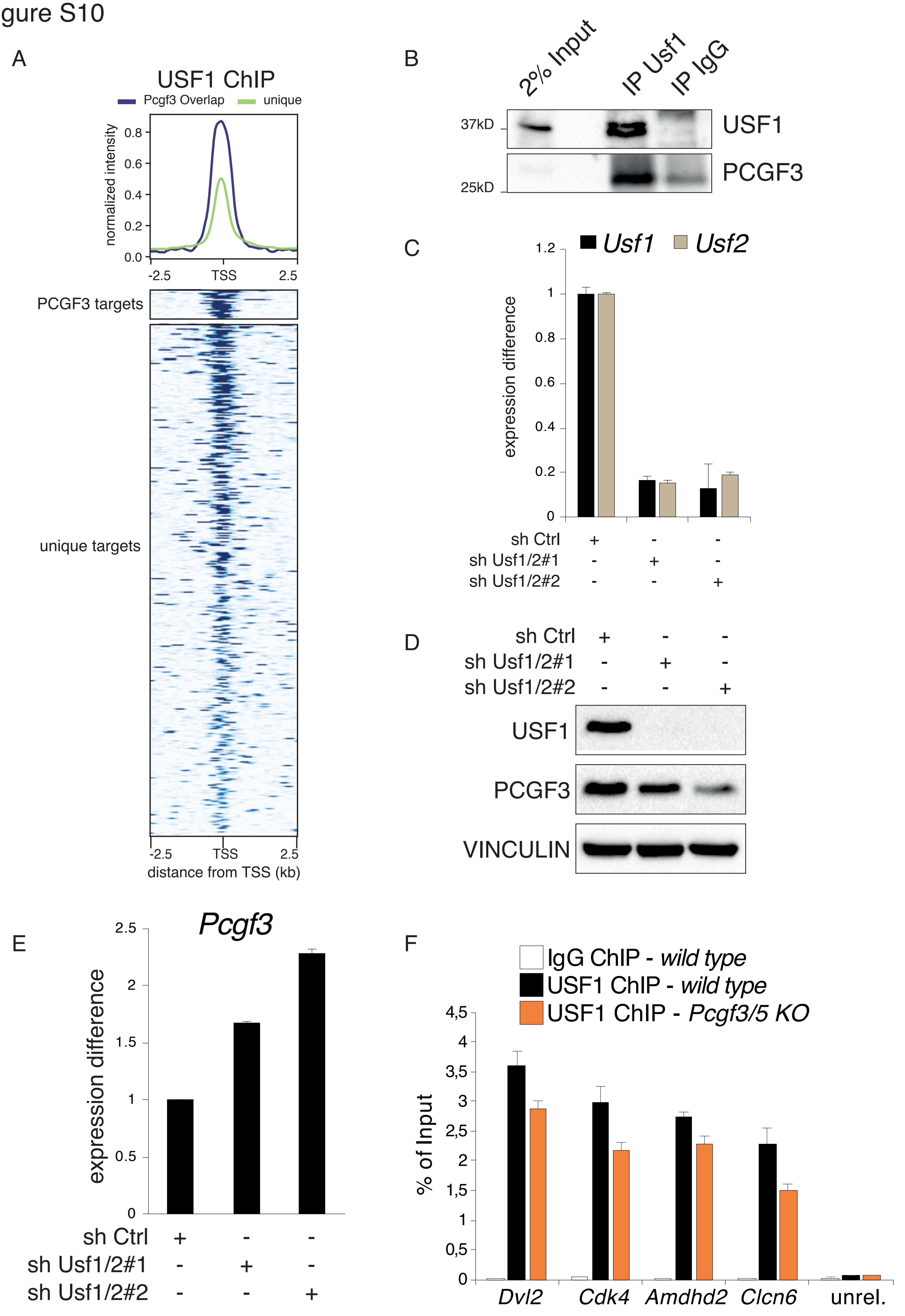
A
USF1 ChIP
Pcgf3 Overlap
unique
normalized intensity
0.8
0.6
0.4
0.2
0.0
-2.5
TSS
2.5
PCGF3 targets
unique targets
distance from TSS (kb)
B
2% Input
IP Usf1
IP IgG
37kD
25kD
USF1
PCGF3
C
Usf1
Usf2
expression difference
1.2
1
0.8
0.6
0.4
0.2
0
sh Ctrl + - -
sh Usf1/2#1 - + -
sh Usf1/2#2 - - +
D
sh Ctrl + - -
sh Usf1/2#1 - + -
sh Usf1/2#2 - - +
USF1
PCGF3
VINCULIN
E
Pcgf3
expression difference
2.5
2
1.5
1
0.5
0
sh Ctrl + - -
sh Usf1/2#1 - + -
sh Usf1/2#2 - - +
F
IgG ChIP - wild type
USF1 ChIP - wild type
USF1 ChIP - Pcgf3/5 KO
% of Input
4,5
4
3,5
3
2,5
2
1,5
1
0,5
0
Dvl2
Cdk4
Amdhd2
Clcn6
unrel.

Supplemental Figure Legends

Figure S1. Genome-wide PCGF Binding Specificity. Related to Figure 1

(A) RT-qPCR analysis showing the mRNA expression levels of the indicated *Pcgf* genes in WT mESC relative to *Ring1b* expression. *Gapdh* served as normalizing expression control. Data represent mean ± SEM.

(B) Relative abundance based on intensity Based Absolute Quantification (iBAQ) values of PCGF peptides identified in RING1B-Flag-bio tandem purifications coupled to mass-spectrometry experiment.

(C) ChIP-qPCR analysis at the *Gal4*-TK-Luciferase promoter of ChIP experiments in the indicated 293Trex clones expressing inducible Gal4 (empty) or Gal4-PCGF fusion proteins, comparing independently the efficiency of the GAL4 to the PCGF1, PCGF2, PCGF3 or PCGF6 antibodies. Rabbit IgG served as a negative control. ChIP enrichments are presented as percentage of input. Data represent mean ± SEM.

(D) Uncropped western blot images of immunoblotting with PCGF6 antibody on WT and *Pcgf6* KO mESC extracts.

(E) Observed/expected ratio of CpG dinucleotides contained within the peaks of the indicated ChIP-seq analyses. Ratio of 0.6 (black-dashed line) or higher is associated with the presence of CpG islands.

(F) Average width of the peaks identified in the indicated ChIP-seq profiles.

(G) Venn diagram showing the overlap between the target genes for the different indicated PCGF proteins.

Figure S2. Genomic and Proteomic Landscape of PRC1 Family Complexes. Related to Figure 2

(A) Boxplots of the input subtracted normalized intensity profiles of ChIP-seq analyses for the indicated proteins and histone modifications over ±4 kb of the indicated loci stratified for PCGF co-occupancy in wild-type WT mESCs. CBX7 and RYBP datasets from mESCs were obtained from (Morey et al., 2013) and KDM2B dataset from (Farcas et al., 2012).

(B) Heatmap of PRC1-associated complexes. The GAL4-PCGF (1-6) PRC1 subunits indicated at the top of the table were immunoprecipitated and the recovered proteins were subjected to Liquid Chromatography-Mass Spectrometry (LC-MS). The LFQ (Label Free quantification) ratio is color-coded and displayed as a heatmap with color intensity representing the degree of enrichment obtained in every specific immunoprecipitation. Proteins are grouped in accordance to which GAL4-PCGF they associate with.

(C) Gene ontology analysis for the indicated PCGF unique and co-occupied target genes. The most represented categories are highlighted. Dot size is proportional to the number of genes corresponding to that gene ontology category, color scale indicates statistical significance (adjusted p-value < 0.01 and q-value < 0.01).

Figure S3. Functional Cross-talk Between Different PCGF Proteins. Related to Figure 3

(A, B) Western blot analyses with the indicated antibodies of protein lysates from WT and indicated *Pcgf* KO mESC clones. Vinculin served as a loading control. *, unspecific signal.

(C) Heatmaps of the input subtracted normalized intensity profiles of ChIP-seq analyses for PCGF1, PCGF2, PCGF3, or PCGF6 performed in WT mESCs, *Pcgf1*, *Pcgf2/4*, *Pcgf3/5*, or *Pcgf6* KO ESC clones. The regions plotted correspond to ±4 kb around TSS of unique and co-occupied target genes, as indicated.

(D) Boxplots of the input subtracted normalized intensity profiles from two distinct biological replicates of ChIP-seq analyses for PCGF1, PCGF2, PCGF3, or PCGF6 performed in WT, *Pcgf1*, *Pcgf2/4*, *Pcgf3/5*, or *Pcgf6* KO mESC clones. Signal enrichment was calculated over ±500 bp around the TSS of the indicated loci stratified for PCGF co-occupancy in WT mESCs.

Figure S4: Chip-Seq Normalization by Spike-in RX Human. Related to Figure 3

(A) Heatmaps of the spike-in input subtracted normalized intensity profiles of ChIP-seq analyses for PCGF1, PCGF2, or PCGF6 performed in WT mESCs, *Pcgf1*, *Pcgf2/4*, *Pcgf3/5*, or *Pcgf6* KO ESC clones. The regions plotted correspond to ±4 kb around TSS of unique and co-occupied target genes, as indicated.

(B) Boxplots of the spike-in input subtracted normalized intensity profiles of ChIP-seq analyses for PCGF1, PCGF2, or PCGF6 performed in WT mESCs, *Pcgf1*, *Pcgf2/4*, *Pcgf3/5*, or *Pcgf6* KO ESC clones. The regions plotted correspond to ±500 bp around TSS of unique and co-occupied target genes, as indicated.

(C) Heatmaps of the input subtracted normalized intensity profiles of ChIP-seq analyses for RING1B, H2Aub1, SUZ12, or H3K27me3 performed in WT mESCs, *Pcgf1*, *Pcgf2/4*, *Pcgf3/5*, or *Pcgf6* KO ESC clones. The regions plotted correspond to ±4 kb around TSS of unique and co-occupied target genes, as indicated.

Figure S5. Phenotypic and Transcriptomic Analyses of *Pcgf* KOs mESC. Related to Figure 4.

(A, B) Western blot analyses with the indicated antibodies of protein lysates from WT and indicated *Pcgf* KO mESC clones. Vinculin served as a loading control. *, unspecific signal.

(C) Representative phase-contrast field of WT, *Pcgf1/2/4* and *Pcgf3/5/6* triple KO mESCs. Scale bars correspond to 200 μm.

(D) Gene ontology analysis for the differentially expressed genes in the indicated *Pcgf* KOs. The most represented categories are highlighted. Dot size is proportional to the number of genes corresponding to that Gene ontology category, color scale indicates statistical significance (adjusted p-value < 0.01 and q-value < 0.01).

(E) Volcano plots of –log10 (P-value) against log2 fold change representing the differences in gene expression between *Pcgf1*, *Pcgf2/4*, *Pcgf3/5*, or *Pcgf6* KO mESC clones and WT for all protein coding genes (upper panels) and for PCGF1, PCGF2, PCGF3 and PCGF6 targets, respectively (bottom panels).

Figure S6. Transcriptomic Analysis of Pluripotency and Differentiation Signatures in *Pcgf* KOs mESC. Related to Figure 4.

(A) Volcano plots of –log10 (P-value) against log2 fold change representing pluripotency related genes, MYC transcriptional network genes from RNA-seq analyses performed in WT and *Pcgf1/2/4* or *Pcgf3/5/6* triple KO mESCs.

(B) Volcano plots of –log10 (P-value) against log2 fold change of genes from multiple embryonic layers signatures obtained from (Hutchins et al., 2017) from RNA-seq analyses performed in WT and *Pcgf1, Pcgf2/4, Pcgf3/5, Pcgf6, Pcgf1/2/4* or *Pcgf3/5/6* KO mESCs.

Figure S7. Analysis of Polycomb Signature at PCGF Targets *i*n Triple KO mESCs. Related to Figure 4.

(A) Gene ontology enrichments based on adjusted p-values ($-\log_{10}$ p) of genes upregulated in *Pcgf3/5/6* triple KO mESCs.

(B) Z-score expression heatmaps for collagen related genes from RNAseq analyses performed in WT, *Pcgf1/2/4* or *Pcgf3/5/6* triple KO mESCs

(C) As in (B) for several indicated keratins related genes.

(D) Boxplots of the input subtracted normalized ChIP-seq intensity profiles of RING1B, H2AK119ub1 (H2Aub1), SUZ12, H3K27me3 performed in WT and *Pcgf1*, *Pcgf2/4* and *Pcgf1/2/4* KO mESC clones. Signal enrichment is calculated over ±500 bp (±4 kb for H2Aub1 and H3K27me3) around the TSS of the indicated group of PCGF target genes.

(E) Genomic snapshots of the ChIP-seq profiles quantified in (A) at selected PCGF1/2 common and PCGF6 unique target gene loci, performed in WT and in the indicated KO mESCs.

(F) Boxplots of the input subtracted normalized ChIP-seq intensity profiles of RING1B, H2AK119ub1 (H2Aub1), SUZ12, H3K27me3 performed in WT, *Pcgf6*, *Pcgf3/5*, and *Pcgf3/5/6* KO mESC clones. Signal enrichment is calculated over ±500 bp (±4 kb for H2Aub1 and H3K27me3) around the TSS of the indicated group of PCGF target genes.

(G) Genomic snapshots of the ChIPseq profiles quantified in C at selected PCGF1/2 common and PCGF6 unique target gene loci performed in WT and the indicated KO mESCs.

(H) Percentage of deregulated PCGF6 targets genes co-occupied by RING1B (first two bars) or PCGF6 unique respect to RING1B (last two bars).

Figure S8. PCGF3 and PCGF6 Recruit Modest RING1B Activity and Present Features of Active Transcription. Related to Figure 5.

(A–D) Heatmap analysis representing the input subtracted normalized ChIP-seq intensities over ±8 kb centered at TSS of PCGF1 (A), PCGF2 (B), PCGF3 (C), PCGF6 (D) target loci stratified for RING1B co-occupancy in WT mESCs. H3K36me3 intensities are shown along the entire gene length (from TSS to TES).

(E) Boxplots of the input subtracted normalized intensity profiles of the indicated ChIPseq analyses performed in A-D. Signal enrichment is calculated over ±8 kb around the TSS of the indicated group of PCGF target genes.

(F) Western blot analysis with the indicated antibodies of protein lysates prepared from *Ring1A*$^{-/-}$;*Ring1B*$^{fl/fl}$;*Rosa26::CreERT2* (R1A KO-R1B FL) mESC treated with 4-OHT at the indicated time points. Vinculin served as loading control. *, unspecific signal.

(G, H) qPCR analysis at selected target regions of sequential RING1B ChIP (re-ChIP) experiments upon PCGF1 (G) or PCGF2 (H) ChIP in WT mESC. IgG served as control for ChIP assay. ChIP enrichments are

normalized to input. Data represent mean ± SEM.

Figure S9. Loss of E2F6, MGA, or MAX Does Not Affect PCGF6 Expression. Related to Figure 6.

(A) Venn diagram of PCGF6 (blue) and MGA (red) target genes.

(B) ChIP-qPCR validation of MGA and E2F6 binding at PCGF6 targets. IgG served as control for ChIP assay. ChIP enrichments are normalized to input. Data represent mean ± SEM.

(C) RT-qPCR analysis of *Mga* and *Pcgf6* mRNA levels in WT mESC expressing scramble (shCtrl) or specific shRNAs targeting *Mga*. *Gapdh* served as normalizing expression control. Fold change over shCtrl is shown. Data represent mean ± SEM.

(D) Western blot analysis with the indicated antibodies of protein lysates from cells described in C. Vinculin served as loading control.

(E) RT-qPCR analysis of *E2f6*, *Max* and *Pcgf6* mRNA levels in WT mESC expressing scramble (sh Ctrl) or shRNAs specifically targeting *E2f6* and *Max* in the indicated combinations. *Gapdh* served as normalizing expression control. Fold change over sh Ctrl is shown. Data represent mean ± SEM.

(F) Western blot analysis with the indicated antibodies of protein lysates from WT and *MgaΔHLH* mESC mutant expressing scramble (sh Ctrl) or shRNAs targeting specifically *E2f6* and *Max*. Vinculin served as loading control.

(G) Heatmaps of input subtracted normalized intensity profiles of MYC in WT mESC and PCGF6 binding in WT or *Pcgf6* KO, a sh*Mga*, *Mga*ΔHLH mutant, sh*E2f6*, and a sh*E2f6* in *Mga*ΔHLH mutant mESCs around ±2.5 kb of the TSS of PCGF6 targets stratified by the presence of E2F, E-box, E2F and E-box and unrelated binding motifs predicted by FIMO.

(H) Boxplots of the input subtracted normalized intensity profiles of ChIP-seq analyses performed in (G) over ±500 bp respect to the TSS.

Figure S10. USF1 Interacts with PCGF3, which is not Affected by USF1 Loss. Related to Figure 7.

(A) Input subtracted normalized intensity profiles and heatmap of USF1 binding in WT mESCs around ±2.5 kb of the TSS of USF1-Pcgf3 common and USF1 unique target loci.

(B) Western blot analysis with the indicated antibodies of USF1 immuno-precipitations using WT mESC nuclear extracts. Mouse IgG served as unrelated antibody. Input is shown as loading control.

(C) RT-qPCR analysis of *Usf1* and *Usf2* mRNA levels in WT mESC expressing scramble (sh Ctrl) or shRNAs targeting specifically *Usf1* and *Usf2* in the indicated combinations. *Gapdh* served as normalizing expression control. Fold-change over sh Ctrl is shown. Data represent mean ± SEM.

(D) Western blot analysis with the indicated antibodies of protein lysates from cells described in (C). Vinculin served as loading control.

(E) RT-qPCR analysis of *Pcgf3* mRNA levels in WT mESC expressing scramble (sh Ctrl) or shRNAs specifically targeting *Usf1* and *Usf2* in the indicated combinations. *Gapdh* served as normalizing expression control. Fold change over sh Ctrl is shown. Data represent mean ± SEM.

(F) USF1 ChIP-qPCR analysis at selected PCGF3 target regions performed in WT and *Pcgf3/5* KO mESCs. IgG served as a negative control for ChIP. ChIP enrichments are normalized to input. Data represent mean ± SEM.

Supplemental Table Legends

Table S1. shRNA and sgDNA sequences. Related to Star Methods

List of oligo sequences used for RNAi and CRISPR/cas9 manipulations of mESCs.

Table S2. PCGF Enriched Genomic Regions. Related to Figure 1

List of peaks called for the different PCGF ChIP in WT mESCs.

Table S3. PCGF Target genes. Related to Figure 1

List of target promoters bound by each PCGF protein in WT mESCs.

Table S4. Gal4-PCGF fusion proteins MS results. Related to Figure 2

Values of the LFQ ratios of the different PRC1.1-6 components obtained by MS/MS analyses in the Gal4-PCGF1-6 fusions immuno-purifications (anti-Gal4) from 293TRex cells.

Table S5. Gene Expression in PCGF KO ESC. Related to Figure 4

Expression values of all RefSeq genes in the different PCGF KO mESC lines.

Table S6. Gene Ontology of Differentially Expressed Genes. Related to Figure 4

List of significant GO terms enriched in differentially expressed genes in the different PCGF KO mESC lines.
