# Supplementary material for: Functional Landscape of PCGF Proteins Reveals Both RING1A/B-Dependent-and RING1A/B-Independent-Specific Activities
Source: Mol Cell. 2019 Jun 6;74(5):1037–1052.e7. doi: 10.1016/j.molcel.2019.04.002 (PMC6561742; doi:10.1016/j.molcel.2019.04.002)
Supplement: Document S2. Article plus Supplemental Information [file mmc8.pdf]

Article

Molecular Cell

# Functional Landscape of PCGF Proteins Reveals Both RING1A/B-Dependent-and RING1A/B-Independent-Specific Activities

## Graphical Abstract

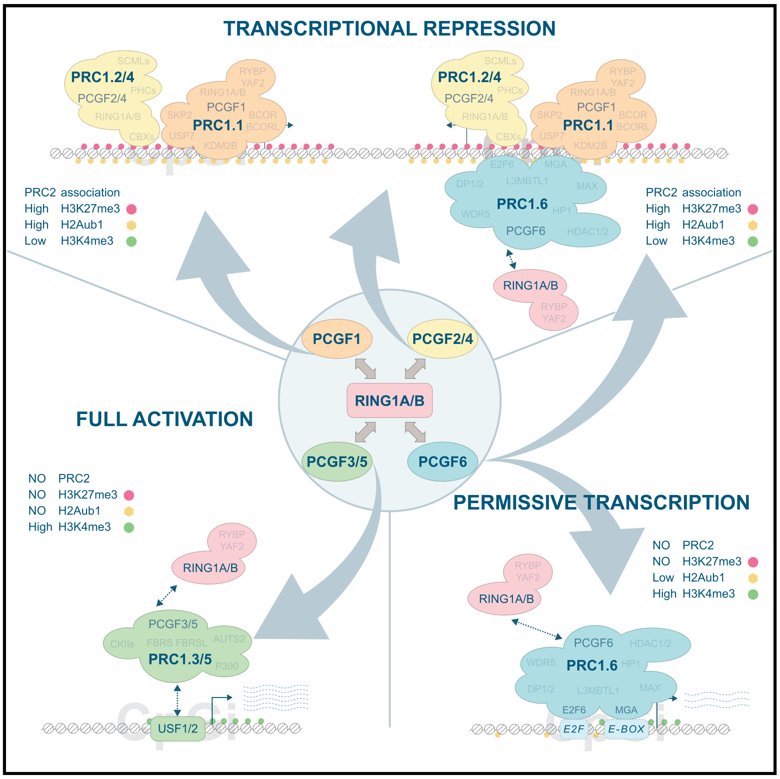


## Highlights

- PRC1 complexes retain high target specificity with little compensatory functions
- PCGF2 defines repressive signatures and PCGF3 and PCGF6 transcription active states
- RING1A/B is dispensable for the assembly and recruitment of PCGF3 and PCGF6 complexes
- PCGF3 and PCGF6 complexes bind target genes through specific DNA binding activities

## Authors

Andrea Scelfo, Daniel Fernández-Pérez, Simone Tamburri, ..., Tiziana Bonaldi, Karin Johanna Ferrari, Diego Pasini

## Correspondence

karinjohanna.ferrari@ieo.it (K.J.F.), diego.pasini@ieo.it (D.P.)

## In Brief

PRC1 encompasses different sub-complexes with distinct biochemical properties. Scelfo et al. dissect these activities, highlighting their little redundancy and high functional specificity. PRC1 sub-complexes associate specifically with transcriptional repression or activation, can function independently of the core RING1A/B enzymes, and can be recruited to chromatin by specific DNA binding activities.

Check for updates

Scelfo et al., 2019, Molecular Cell *74*, 1037–1052
June 6, 2019 © 2019 The Authors. Published by Elsevier Inc.
https://doi.org/10.1016/j.molcel.2019.04.002

CellPress

# Functional Landscape of PCGF Proteins Reveals Both RING1A/B-Dependent- and RING1A/B-Independent-Specific Activities

Andrea Scelfo,[1,3,5] Daniel Fernández-Pérez,[1,2,3] Simone Tamburri,[1,3] Marika Zanotti,[1] Elisa Lavarone,[1] Monica Soldi,[1] Tiziana Bonaldi,[1] Karin Johanna Ferrari,[1,4,*] and Diego Pasini[1,2,4,6,*]
[1]IEO European Institute of Oncology IRCCS, Department of Experimental Oncology, Via Adamello 16, 20139 Milan, Italy
[2]University of Milan, Department of Health Sciences, Via A. di Rudinì, 8, 20142 Milan, Italy
[3]These authors contributed equally
[4]These authors contributed equally
[5]Present address: Institut Curie, PSL Research University, UMR 144, 26 rue d'Ulm, F-75005 Paris, France
[6]Lead Contact
*Correspondence: karinjohanna.ferrari@ieo.it (K.J.F.), diego.pasini@ieo.it (D.P.)
https://doi.org/10.1016/j.molcel.2019.04.002

## SUMMARY

**Polycomb repressive complexes 1 and 2 (PRC1 and PRC2) control cell identity by establishing facultative heterochromatin repressive domains at common sets of target genes. PRC1, which deposits H2Aub1 through the E3 ligases RING1A/B, forms six biochemically distinct subcomplexes depending on the assembled PCGF protein (PCGF1–PCGF6); however, it is yet unclear whether these subcomplexes have also specific activities. Here we show that PCGF1 and PCGF2 largely compensate for each other, while other PCGF proteins have high levels of specificity for distinct target genes. PCGF2 associates with transcription repression, whereas PCGF3 and PCGF6 associate with actively transcribed genes. Notably, PCGF3 and PCGF6 complexes can assemble and be recruited to several active sites independently of RING1A/B activity (therefore, of PRC1). For chromatin recruitment, the PCGF6 complex requires the combinatorial activities of its MGA-MAX and E2F6-DP1 subunits, while PCGF3 requires an interaction with the USF1 DNA binding transcription factor.**

## INTRODUCTION

The precise control of specific active and repressed transcriptional states is at the basis of first establishing and then maintaining cellular identity (Bracken and Helin, 2009; Orkin and Hochedlinger, 2011). The Polycomb group (PcG) protein family provides the major repressive mechanism for defining facultative heterochromatin (Bernstein et al., 2006), an essential step for both embryogenesis and homeostatic development of adult tissues (Aloia et al., 2013; Avgustinova and Benitah, 2016). Polycomb proteins exert their functions in two large multiprotein repressive complexes, PRC1 and PRC2, which are defined by specific core activities that modify histone proteins. PRC2 deposits mono-, di-, and tri-methylation on the lysine 27 of histone H3 (H3K27me1/me2/me3), catalyzed by the EZH1/2 methyltransferases (Ferrari et al., 2014; Margueron et al., 2008; Shen et al., 2008); PRC1 mono-ubiquitinates histone H2A lysine 119 (H2Aub1), which is catalyzed by the E3 ligase RING1A or RING1B (de Napoles et al., 2004; Endoh et al., 2008). These two activities control common regulatory pathways by co-associating to a large extent at the same set of target genes (Simon and Kingston, 2013).

Although core enzymatic activities are conserved, PRC1 and PRC2 form distinct subcomplexes defined by the association of ancillary subunits (Scelfo et al., 2015). In PRC1, RING1A/B can interact with one of six distinct, mutually exclusive members of the PCGF protein family (PCGF1–PCGF6), thereby creating six distinct PRC1 subcomplexes (PRC1.1–PRC1.6) that dictate the recruitment of specific ancillary subunits with diverse functional properties (Di Croce and Helin, 2013). Importantly, PCGF2 and PCGF4, or PCGF3 and PCGF5, independently assemble biochemically identical complexes bearing redundant functional properties (Gao et al., 2012). Reasonably, up to four major activities for PRC1 could exist and be active in the same cells, in close relationship with PRC2, but with potentially distinct functions (Pasini and Di Croce, 2016).

PRC1.2 and PRC1.4 are also termed the canonical PRC1 complexes (and the other subcomplexes, non-canonical), based on H3K27me3 recognition deposited by PRC2 (Scelfo et al., 2015). Specifically, the CBX proteins in PRC1.2/PRC1.4 (not present in the other subcomplexes; Gao et al., 2012) bind the H3K27me3 moiety via their chromodomain (Cao et al., 2002; Fischle et al., 2003; Min et al., 2003). How non-canonical subcomplexes are recruited to chromatin remains less well understood. Recruitment of PRC1.1 depends on its KDM2B subunit, which can recognize unmethylated CpG islands (Blackledge et al., 2014). The PRC1.6 complex contains different proteins with DNA binding activity (E2F6-DP1 and MGA-MAX dimers) that could allow direct binding to DNA (Gao et al., 2012). In contrast, the PRC1.3 and PRC1.5 complexes contain no subunits with defined DNA or chromatin binding properties, and their recruitment mechanisms remain unclear.


This is an open access article under the CC BY license (http://creativecommons.org/licenses/by/4.0/).

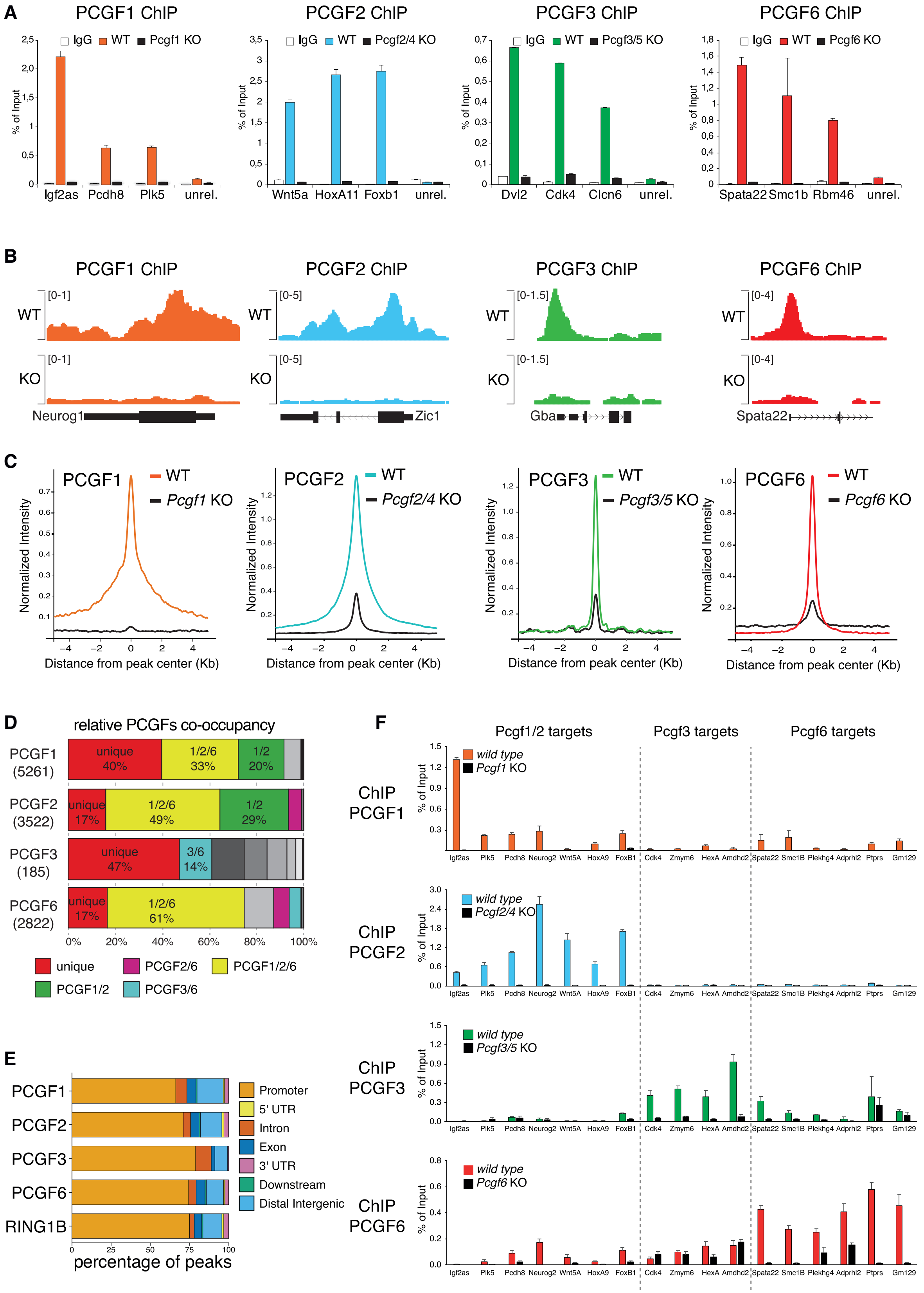


(legend on next page)

The activities of non-canonical PRC1 complexes can promote the recruitment and/or stabilization of PRC2 to chromatin (Blackledge et al., 2014; Farcas et al., 2012). This involves the intrinsic ability of PRC2 to bind H2Aub1 deposited by RING1A/B (Cooper et al., 2016; Kalb et al., 2014).

Loss of RING1A/B activity results in pre-implantation lethality at the two-cell stage (Posfai et al., 2012). All distinct PCGF activities play major but distinct roles in development (Akasaka et al., 2001; Almeida et al., 2017; Endoh et al., 2017) (http://www.mousephenotype.org/data/genes/MGI:1917087). Indeed, none of them independently reproduce the loss of RING1A/B activity, suggesting that distinct PCGF activities may act together to determine RING1A/B biological functions. In this context, several questions still remain unanswered. How are the activities of the distinct PRC1 subcomplexes regulated? Do they act redundantly to control similar pathways, or do they (also) have specific functional features?

Here, we provide a comprehensive dissection of the functional landscape of PCGF proteins, characterizing the crosstalk among the different complexes, their relationship with PRC2 activity, and the recruitment mechanisms that mediate their interactions with chromatin and DNA. By combining the development of highly specific PCGF1-6 antibodies with the generation of KO mouse embryonic stem cells (ESC) lines, we mapped the physiological genome-wide occupancy of all PRC1 subcomplexes to determine their functional control. We show that PCGF proteins retain high levels of binding specificity, with little crosstalk among the different complexes with the exception of PCGF1 and PCGF2, which displayed extensive functional overlap. We demonstrate that, while PCGF1 and PCGF2 activities are strongly linked with transcriptional repression, PCGF3 and PCGF6 are mainly associated with active transcriptional states, even in the absence of RING1A/B recruitment. Importantly, the PCGF3- and PCGF6-containing complexes did not require RING1A/B for their assembly and recruitment to chromatin, providing evidence that both complexes are recruited to target genes by intrinsic and specific DNA binding modules.

## RESULTS

### Distinct PRC1 Subcomplexes Regulate Specific Sets of Target Genes

We analyzed both transcription levels and mass spectrometry to determine the relative amounts of distinct PRC1 subcomplexes active in ESCs and found that the PCGF6- and PCGF2-containing complexes were the most abundant forms of PRC1 (Figures S1A and S1B). PCGF4 and PCGF5 were only present in trace amounts, consistent with their poor expression in ESCs. These results agreed with previous measurements (Kloet et al., 2016) and further showed that the functional PRC1 subcomplexes in ESCs are defined by the presence of PCGF1, PCGF2, PCGF3, and PCGF6, confirming that all four functionally distinct forms of PRC1 are present in ESCs.

We developed highly specify antibodies for each PCGF protein and engineered ESC lines to individually create *Pcgf1* and *Pcgf6* knockouts (KO) and *Pcgf2/4* and *Pcgf3/5* double-KO (STAR Methods; Table S1) to avoid any potential compensatory effects of redundant PCGF proteins (Figures 1A and 1B) and mapped the PCGF1, PCGF2, PCGF3, and PCGF6 occupancies along the ESC genome by ChIP-seq assays (Figures 1B and 1C). We found that PCGF1 had the most extensive binding repertoire, with 5,261 target genes, followed by PCGF2 (3,522), PCGF6 (2,822), and PCGF3 (185) (Figure 1D). These differences were not due to diverse antibodies efficiencies (Figures S1C and S1D) and did not echo the relative abundance of subcomplexes (Figure S1B). Similar to RING1B, all PCGF proteins preferentially associated to promoter elements (> 75%; Figure 1E) and showed affinity for high CpG dinucleotides density (Figure S1E). PCGF2 occupied broader regions while other PCGF proteins displayed sharper associations, suggesting different modes of chromatin interactions (Figure S1F). By overlapping the enriched genomic regions of each PCGF protein, we found that more frequent combinations of promoter co-occupancy emerged (e.g., PCGF1/2 and PCGF1/2/6) (Figures 1D and S1G). However, these results demonstrated that PCGF proteins also retain high specificity in genomic occupancy, as confirmed by ChIP-qPCR analysis (Figure 1F).

### PCGF Proteins Associate with Distinct Functional Domains

We next examined whether distinct PCGF proteins associate to promoter regions that have similar or unique functional properties. First, we defined promoters exclusively-occupied and co-occupied by different PCGFs (Figures 2A and S2A; Tables S2 and S3). Then, we analyzed, on those regions, the presence of general components of the two Polycomb machineries (SUZ12, RING1B, RYBP, and CBX7); WDR5, component of several multiprotein complexes including PRC1.6, COMPASS, and basal transcriptional machineries (Guarnaccia and Tansey, 2018); the unmethylated CpG binding protein KDM2B (Farcas et al., 2012); and histone post-translational modifications (PTMs) associated with activation (H3K4me3 and H3K36me3) or repression (H3K27me3 and H2Aub1) (Figures 2A and S2A). We found that PCGF2 was always associated with a Polycomb repressive signature and

**Figure 1. PCGFs Show Specificity in Target Gene Occupancy**

(A) ChIP-qPCR analysis for the indicated PCGF proteins at selected target regions in wild-type (WT) and in indicated *Pcgf* KO mouse ESCs. IgG served as control for ChIP assay. ChIP enrichments are normalized to input. Data represent mean ± SEM.

(B) Genomic snapshots of the indicated ChIP-seq profiles at selected gene loci performed as in (A).

(C) ChIP-seq cumulative enrichment deposition centered at peak summit for the indicated PCGF proteins performed as in (A).

(D) Percentage of co-occupancy of the target genes identified for each indicated PCGF protein with respect to the other datasets. For simplicity, just regions that represent 14% or more of the total PCGF targets are shown in the legend.

(E) Genome-wide functional annotation of peaks generated from the indicated ChIP-seq analyses. Promoters are defined as the region around ±2.5 kb from mm9-annotated TSS, and the downstream regions as the first 3 kb after the TES.

(F) ChIP-qPCR analysis for the indicated PCGF proteins at selected target regions in the indicate mESC lines.

See also Figure S1 and Tables S2 and S3.

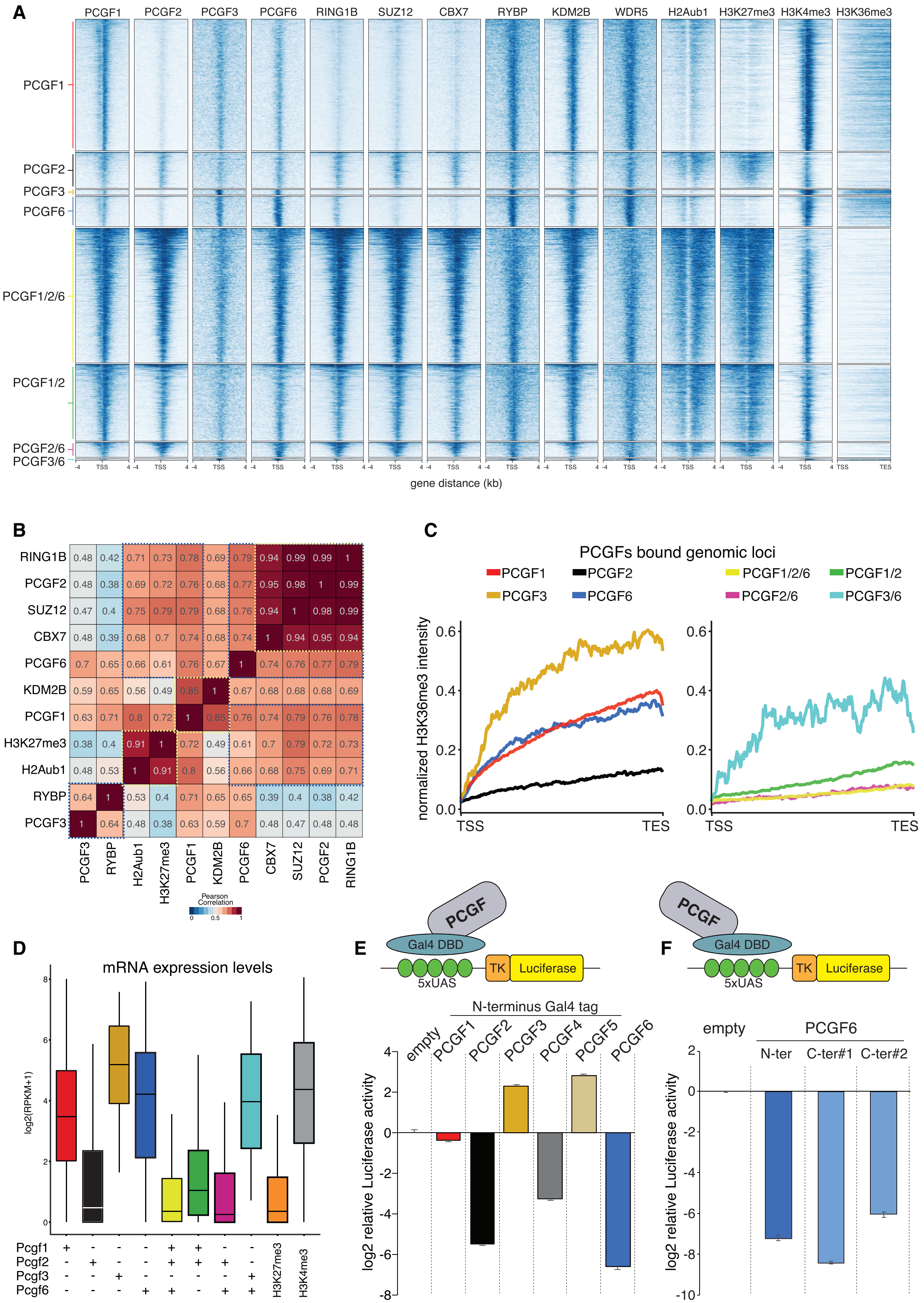


(legend on next page)

that, in the absence of PCGF2, PCGF3, and PCGF6, were associated with a transcriptional permissive status (e.g., high H3K4me3 and H3K36me3), with PCGF3 target genes showing the highest transcriptional activity (Figures 2B and 2C). Similarly, only PCGF1 unique targets, devoid of PCGF2 co-occupancy, displayed a permissive transcriptional activity (Figure 2C), strengthening the correlation between PCGF2 binding and transcriptional repression. These results were further confirmed by transcriptional RNA-seq analyses (Figure 2D).

We next assayed the transcriptional properties of PCGF proteins by independent tethering (as GAL4-PCGF chimeras) to an ectopic artificial promoter that controlled the luciferase gene (as a readout). PCGF2 and PCGF4 strongly repressed luciferase expression, while PCGF3 or PCGF5 activated it (Figure 2E), consistent with their association with repressed and actively transcribed genes, respectively (Figure 2D). PCGF6 strongly repressed luciferase expression (Figures 2E and 2F), suggesting that this complex could have repressive properties also at its transcribed target promoters. MS analysis of the Gal4-PCGF fusions demonstrated normal complexes assembly (Figure S2B; Table S4) to all previously described PRC1 partner proteins (Gao et al., 2012).

Gene ontology for PCGF2-associated genes showed a significant enrichment for development-related processes, irrespective of their co-occupancy with other PCGF proteins (Figure S2C). In contrast, PCGF3- and PCGF6-associated genes were enriched for distinct ontologies related to autophagy or meiosis/spermatogenesis, respectively, denoting distinct functional properties (Figure S2C).

## PCGF Loss Does Not Result in Functional Compensation but Influences PRC1 Subcomplex Activity

To determine whether PRC1 subcomplexes had specific compensatory features, we quantified the chromatin association of PCGF1, PCGF2, PCGF3, and PCGF6 in all the *Pcgf* KO ESC lines by ChIP-seq (Figures 3A). PCGF protein levels and core components of PRC1 and PRC2 remained overall stable (Figures S3A and S3B). Consistently, PCGF proteins' occupancy was not altered at their specific binding sites (Figures 3B, S3C, and S3D), an effect that was confirmed using a reference genome (ChIP-RX) for signal normalization (Figures S4A and S4B). PCGF proteins did not compensate for each other. For instance, PCGF2 did not bind at PCGF6 target sites in *Pcgf6* KO ESCs (Figures 3B, S4A, and S4B). Similarly, PCGF1 was not affected at PCGF2-target regions in *Pcgf2/4* KO ESCs and vice versa. Overall, these data showed that, in absence of specific PCGF activities, target specificity is largely maintained.

We next quantified binding of RING1B (for PRC1) and SUZ12 (for PRC2), as well as deposition of H2Aub1 and H3K27me3, at each specific group of targets (Figures 3C and S4C). Regions occupied by PCGF2, regardless of which PCGF protein was co-associated, presented much higher RING1B and SUZ12 association as well as H2Aub1 and H3K27me3 deposition than those occupied by PCGF3 and PCGF6 (Figures 3C and S4C). RING1B association was reduced at PCGF2 binding sites in *Pcgf2/4* KO, as well as at PCGF6 binding sites in *Pcgf6* KO. PCGF1 loss had no effect on RING1B association, likely due to PCGF2 compensation. However, RING1B association was only partially lost in *Pcgf2/4* KO, likely compensated by PRC1.1. H2Aub1 deposition was maintained at targets in *Pcgf1* and *Pcgf2/4* KOs, suggesting full compensation between PCGF1 and PCGF2. Differently, RING1B association and H2Aub1 deposition were specifically lost at PCGF3/6 or PCGF6 targets in *Pcgf6* KO ESCs (Figures 3C and S4C). Together, these results revealed that PCGF1 and PCGF2 could compensate for each other specifically at their repressed co-occupied sites but that PCGF6 independently controlled RING1B activity and PRC2 recruitment at sites with substantial transcriptional activity.

## PCGF1/2/4 Module Preserves H2Aub1 Deposition at Repressed Sites but Is Dispensable for ESC Viability

Based on these findings, we defined PCGF1/2/4 activity as the PRC1 repressive module and PCGF3/5/6 as the PRC1 activating module, and generated *Pcgf*1/2/4 and *Pcgf3*/5/6 triple KO ESCs (Figures S5A and S5B). While *Pcgf1/2/4* KO showed no effects on cell viability, *Pcgf3/5/6* KO clones displayed severe morphological changes acquiring a flattened fibroblast-like shape (Figures 4A and S5C). Principal component analysis (PCA) from RNA-seq profiles showed that the transcriptome from *Pcgf1/2/4* KO cells was largely unaltered as compared to wild-type, *Pcgf1* KO, or *Pcgf2/4* KO ESCs (Figures 4B, 4C, S5D, and S5E; Table S5). Loss of *Pcgf6* alone induced significant phenotypic changes, in agreement with previous reports (Yang et al., 2016), enhanced by concomitant loss of PCGF3/5 activities (*Pcgf3/5/6* KO). RNA-seq analyses showed alterations in

**Figure 2. Specific PCGF Activities Define Activating and Repressive Modules**

(A) Heatmaps representing the normalized ChIP-seq intensities for the indicated PCGF proteins over ±4 kb around the TSS of the indicated loci stratified for PCGF co-occupancy in wild-type (WT) mESCs. H3K36me3 intensity was analyzed over the entire gene length (from TSS to TES). CBX7 and RYBP datasets from mESCs were obtained from Morey et al. (2013) and KDM2B from Farcas et al. (2012).

(B) Pearson correlation of ChIP-seq signal over the promoter regions (±4 kb from TSS) of annotated RefSeq coding genes (mm9).

(C) Average deposition profile of H3K36me3 in WT mESCs over the gene body (from TSS to TES) of PCGF unique bound promoters (left panel) or promoters co-occupied by at least two PCGF proteins (right panel), as indicated.

(D) Boxplots showing the expression levels obtained from RNA-seq analyses performed in WT mESC for the indicated PCGF target genes. H3K27me3- and H3K4me3-positive loci served as controls for repressed and active promoters, respectively.

(E) Upper panel: GAL4-TK-luciferase reporter system of 293TRex clones expressing inducible Gal4 (empty) or the indicated Gal4-PCGF fusion protein. Lower panel: Luciferase activity triggered by Gal4-fusion recruitment at GAL4-TK-Luciferase promoter is shown as the fold difference relative to the empty control. Luciferase activity was normalized to protein content. Data represent mean ± SEM.

(F) Upper panel: GAL4-TK-luciferase reporter system of 293TRex clones expressing inducible Gal4 (empty) or the indicated Gal4-PCGF6 fusion protein. Lower panel: Luciferase assay (as in E) with PCGF6 N-terminally or C-terminally fused to the DNA binding domain of Gal4. The activity is shown as the fold difference relative to the empty control and was normalized to protein content. Data represent mean ± SEM.

See also Figure S2 and Table S4.

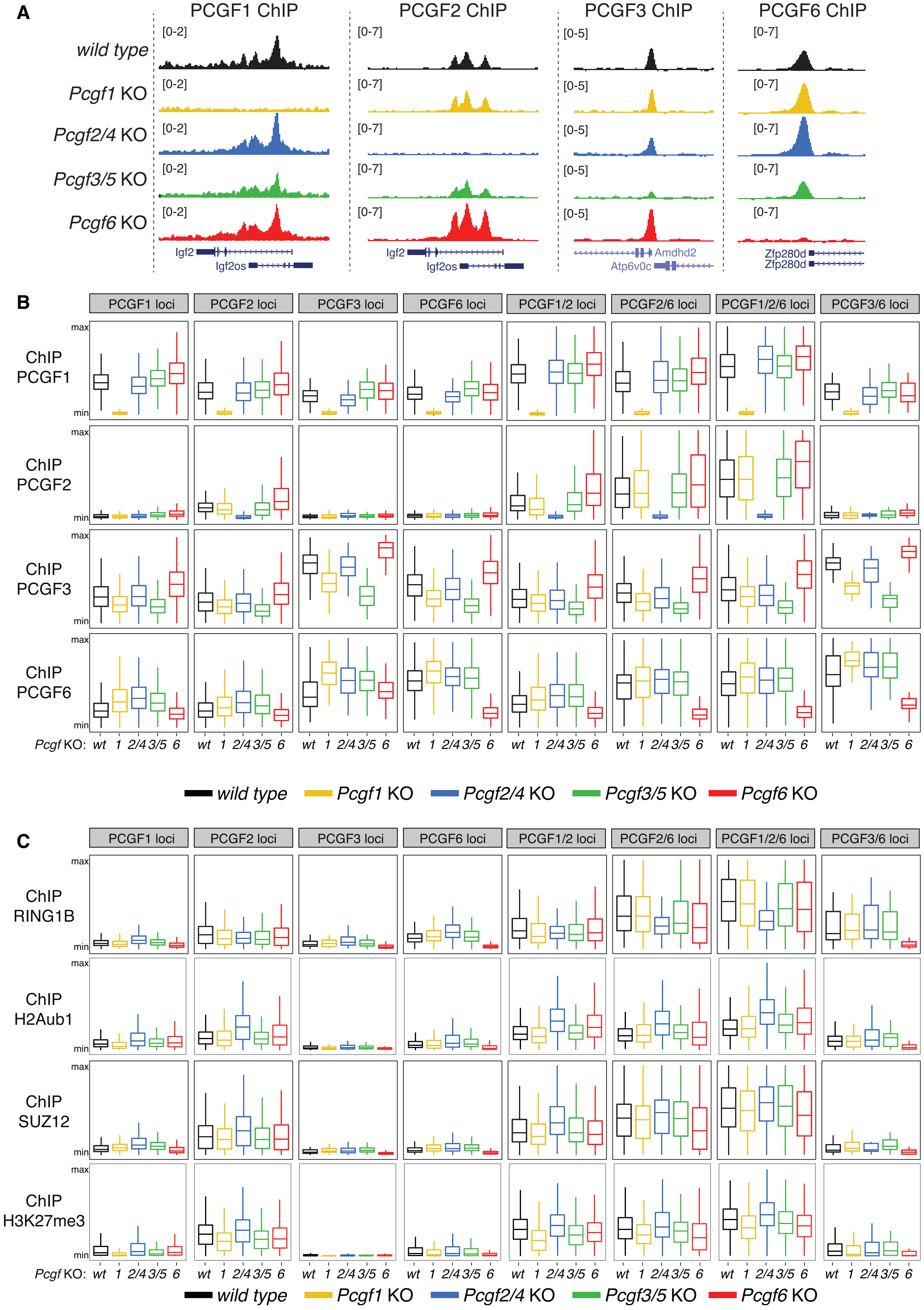


(legend on next page)

expression of genes implicated with various developmental processes, overall highlighting a compromised ESC identity (Figures 4B, 4C, S5D, and S5E). However, this occurred in the absence of a major induction of early differentiation programs (Figure S6) (Hutchins et al., 2017). In particular, *Pcgf3/5/6* KO ESCs were characterized by extracellular matrix and cornification enriched ontologies (Figure S7A; Table S6) that corresponded to a reorganization of actin fibers (Figure 4A) and massive upregulation of collagens and keratins (Figures S7B and S7C).

Combined loss of the PCGF1/2/4 resulted in complete RING1B displacement and loss of H2Aub1 specifically at all PCGF2-occupied loci (Figures 4D, 4E, S7D, and S7E), remaining unaltered in *Pcgf3/5/6* KOs (Figures 4F, 4G, S7F, and S7G). This further correlated with decreased PRC2 (SUZ12) association and reduced H3K27me3 deposition (Figures 4D, 4E, S7D, and S7E). Lack of H2Aub1 deposition did not result in a significant activation of these targets (Figures 4B and 4C). In contrast, loss of PCGF6 led to diffuse gene upregulation (Figures 4B, 4C, and S7H), which occurs in absence of PCGF1/2 displacement and loss of H2Aub1 from co-occupied promoters (Figure 3B, S4C, S5A, and S5B). This supports Gal4-PCGF6 repressive activity and the H2Aub1-independent transcriptional properties of PRC1 (Illingworth et al., 2015; Pengelly et al., 2015).

## RING1A/B Mediates Complex Assembly and Chromatin Binding for PCGF1 and PCGF2, but Not for PCGF3 and PCGF6

A detailed quantification of RING1B co-occupancy with each PCGF protein showed that 70% of all RING1B binding sites overlapped with at least one PCGF protein (Figure 5A). Approximately 60%, 40%, and 80% of PCGF1-, PCGF6-, and PCGF3-bound genomic loci, respectively, were not significantly enriched for RING1B association (Figure 5B). This lack of RING1B co-occupancy correlated with (1) a lack of repressive marks (H3K27me3 or H2Aub1) and (2) an accumulation of an activating signature (H3K4me3 and H3K36me3) (Figures S8A–S8E). These observations suggested that RING1B was indeed absent from these sites. To gain further evidence for the role of RING1B in regulating different PCGF complexes functions, we performed ChIP-seq analysis for each PCGF protein after RING1A/B loss of function (LoF) using a *Ring1A*$^{-/-}$; *Ring1B*$^{fl/fl}$; *Rosa26::CreERT2* conditional mouse ESC line (Endoh et al., 2008) (termed herein *R1A* KO-*R1B* FL). Treatment with 4-hydroxy tamoxifen (OHT) conditionally deletes *Ring1B*, leading to complete loss of RING1B expression within 48 hr (Figure S8F). ChIP-seq analysis for PCGF1 and PCGF2 at 60 hr after OHT treatment showed global displacement of both proteins from chromatin, demonstrating that RING1A/B expression is essential for PCGF1/2 recruitment (Figures 5C, 5D, S8G, and S8H). PCGF2 levels were strongly destabilized in the absence of RING1A/B, suggesting that RING1A/B are required for the assembly of the PRC1.2 complex (which mediates the stabilization of PCGF2) (Figure S8F). In contrast, similar analyses for PCGF3 and PCGF6 revealed that loss of RING1A/B expression did not affect their genome-wide localization (Figures 5E and 5F). Together, these results strongly suggested that while RING1A/B plays an essential role in the assembly and recruitment of the PRC1.1 and PRC1.2 complexes, it is dispensable for PCGF3 and PCGF6 chromatin association. This reinforces the possibility that several PCGF3 and PCGF6 targets do not require RING1A/B association.

## E2F and the E-Box Motif Cooperate to Recruit the PCGF6 Complex to Target Sites

To further explore the properties of the PRC1.6, we investigated its biochemical and recruitment features. Co-immunoprecipitation (coIP) analyses for PCGF6 in *R1A* KO-*R1B* FL ESCs (treated or not with OHT) and *Pcgf6* KO ESCs revealed that, consistently with ChIP-seq results, components of the PRC1.6 remained associated with PCGF6 even in the absence of RING1A/B expression (e.g., L3MBTL2, MAX, and WDR5) (Figure 6A). We performed *de novo* motif discovery on PCGF6 peaks and found that MYC (E-box) and E2F sites were the most enriched and represented DNA motifs (Figure 6B). This strongly agrees with the presence of E2F6-DP1 and MGA-MAX heterodimers among the components of the PCGF6 complex (Hauri et al., 2016; Ogawa et al., 2002). Indeed, PCGF6 occupancy in ESCs almost completely overlapped with MGA genomic distribution (Stielow et al., 2018) (Figure S9A). This result was further confirmed by ChIP-qPCR performed with MGA and E2F6 antibodies (Figure S9B). To further explore the contribution of these DNA binding activities in PCGF6 recruitment, we knocked down MGA using different shRNA sequences (Figures S9C and S9D). Notably, PCGF6 was displaced from all its target sites upon MGA downregulation, as shown by ChIP analysis for PCGF6 (Figure 6C). However, in an ESC line with a MGA C-terminal deletion of the HLH domain (*Mga*ΔHLH), PCGF6 binding was only moderately affected (~2-fold; Figures 6D–6F). These results demonstrated that E-box recognition was not sufficient to impair PCGF6 recruitment but suggested that MGA has broader structural functions in mediating proper PCGF6 complex assembly. CoIP analysis for PCGF6 in both *Mga*ΔHLH ESCs or after *Mga* shRNA-mediated knockdown revealed that the MGAΔHLH mutation did not affect the association of PCGF6 with its different interacting partners but that downregulation of MGA expression disrupted the PCGF6 complex (Figure 6G).

We next tested whether the E2F6-DP1 dimer provided additional DNA binding affinity to the PCGF6 complex. We induced loss of E2F6 or MAX expressing specific shRNAs in wild-type or *Mga*ΔHLH mutant ESCs (Figures S9E and S9F) and analyzed

**Figure 3. PCGFs Are Specific with Little Compensatory Crosstalk**

(A) Genomic snapshots of the indicated ChIP-seq profiles at selected target gene loci, performed in WT and in the indicated *Pcgf* KO mESC clones.

(B and C) Boxplots of the normalized intensity profiles of ChIP-seq analyses for PCGF1, PCGF2, PCGF3, or PCGF6 (B) and for RING1B, H2AK119ub1 (H2Aub1), SUZ12, or H3K27me3 (C), performed in WT mESCs, *Pcgf1*, *Pcgf2/4*, *Pcgf3/5*, or *Pcgf6* KO ESC clones. Signal enrichment was calculated using a region ±4 kb at unique and co-occupied target genes, as indicated.

See also Figures S3 and S4.

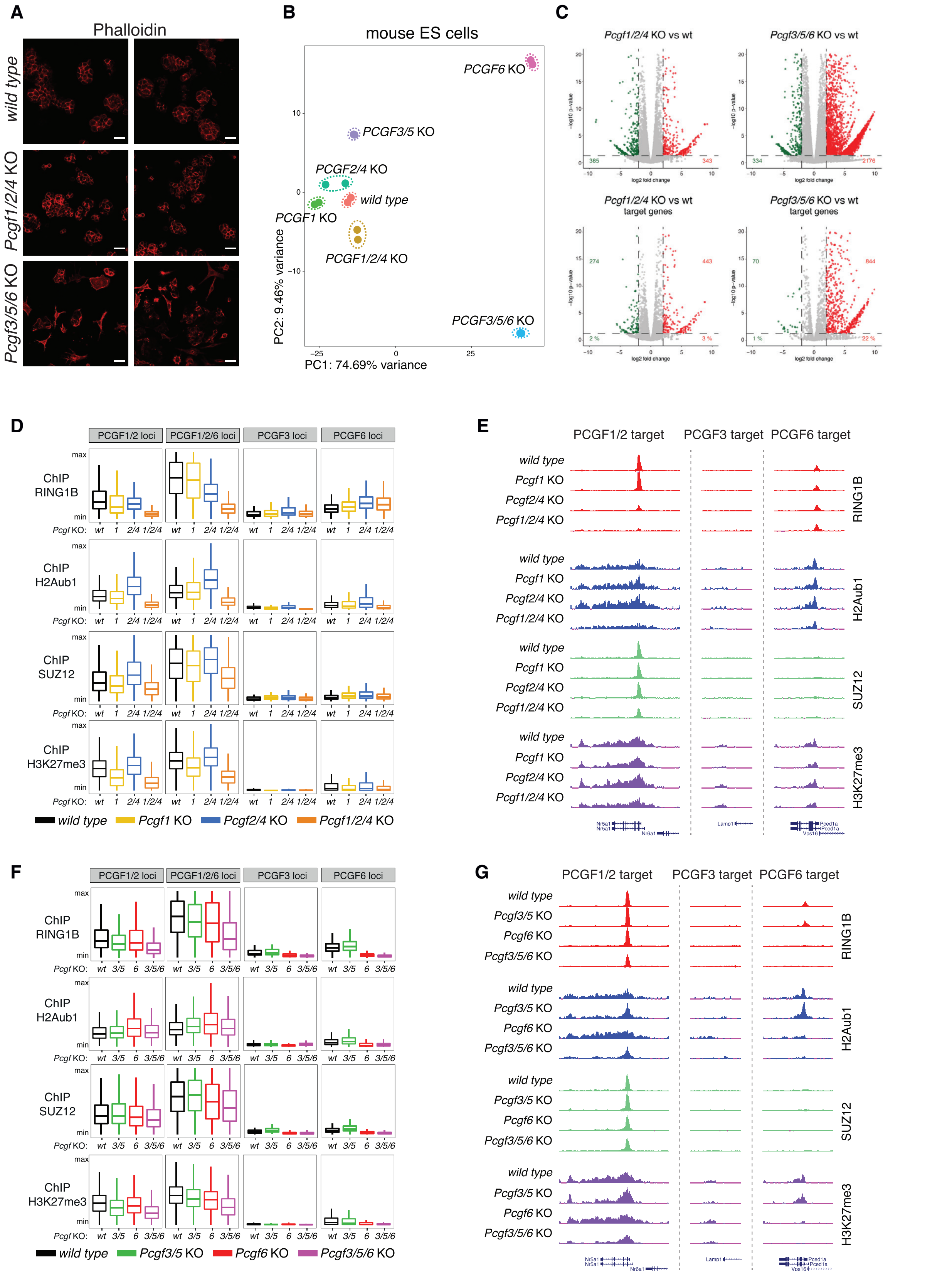


(legend on next page)

PCGF6 chromatin association by ChIP. The combined loss of MGA and E2F6 DNA binding activities further reduced PCGF6 recruitment to its target sites (Figures 6D, 6E, 6H, S9G, and S9H). This strongly suggests that E2F and E-box recognition by intrinsic subunits of the PCGF6 complex mediates DNA binding specificity. It is important to highlight that PCGF6 binding was clearly affected but not completely abolished by E2F6 shRNA (Figures 6D, 6E, and 6H). Although this could be a consequence of an incomplete loss of E2F6 expression (Figure S9F), this result may also suggest that additional recruitment mechanisms (i.e., via L3MBTL2; Huang et al., 2018; Trojer et al., 2011) contribute to recruiting the PCGF6 complex to its specific target loci.

### USF1/2 Interacts with the PCGF3 Complex and Mediates Its Chromatin Recruitment

PCGF3 associated with high specificity to a restricted number of transcribed target promoters in a RING1A/B (PRC1)-independent manner. As no evident DNA binding activities were previously associated to the PCGF3 complex (Gao et al., 2012), we performed *de novo* motif discovery analyses on PCGF3 peaks. The only enriched motif perfectly matched an E-box variant corresponding to the DNA binding site of USF1/2, an HLH-containing transcription factor that forms a heterodimer similar to MYC-MAX (Kiermaier et al., 1999) (Figure 7A). In accordance, ChIP-seq analyses of USF1 in wild-type ESCs revealed a strong USF1 enrichment at almost all PCGF3-bound loci (88%; Figures 7B–7D and S10A). This enrichment was specific for PCGF3 and overlapped only marginally with other PCGF binding sites (Figures 7D and 7E). As USF1 has not been previously reported to interact with PCGF3, we tested USF1–PCGF3 interaction using milder coIP conditions after digesting DNA with benzonase. USF1 showed a clear DNA-independent association with the PCGF3 complex (Figures 7F and S10B). Size-exclusion chromatography further confirmed this result: USF1 co-eluted in a high molecular weight fractions (>650 kD) together with several components of the PCGF3 complex (Figure 7G). We next downregulated *Usf1* and *Usf2* expression with different shRNAs in wild-type ESCs (Figures S10C and S10D). ChIP-seq analysis under these conditions showed that PCGF3 was displaced from all PCGF3 target sites in the absence of USF1/2 expression (Figures 7H and 7I). In contrast, DNA binding of USF1 was not affected by loss of PCGF3 at target sites (Figures S10E and S10F). Together, these results demonstrated that USF1 and USF2 function as DNA binding hubs that mediate recruitment of the PCGF3 complex to DNA.

## DISCUSSION

### PCGF Proteins Have High Specificity for Target Genes

Our data provide the first genome-wide analysis of different PCGF protein activities at a physiological level. These results clearly showed that PCGF proteins display high binding specificity with little functional overlap, with the exception of PCGF1 and PCGF2. PCGF1 has a broad pervasive binding at a large set of CpG-rich promoters, which correlates with RYBP binding and with the broad occupancy of KDM2B. Importantly, H2Aub1 deposition was affected only when loss of PCGF1 and PCGF2/4 were combined, suggesting full enzymatic compensation of these complexes without altering their overall recruitment. Based on these results, we speculate that PCGF1 and PCGF2 act redundantly and enzymatically engage the same target sites in the absence of any evident competition. PCGF6 also showed substantial overlap with PCGF1/2 but with a marginal role in compensating H2Aub1 deposition at these sites. The remaining PCGF6 sites essentially lacked co-association of any other PCGF protein, and loss of PCGF6 did not result in significant cross-compensations. Finally, PCGF3 had few but very specific binding sites at promoters, and it only marginally overlapped with PCGF6. Together, these results highlight the functional specificity of the distinct PRC1 subcomplexes, demonstrating that while PRC1.1 and PRC1.2 cooperate to regulate the same pathways, PRC1.3 and PRC1.6 retain high target specificity and little crosstalk with the activity of the other complexes. Surprisingly, combined loss of PCGF1/2/4 and lack of H2Aub1 deposition at repressed genes did not induce a significant transcriptional reactivation. In contrast, loss of PCGF6 activity resulted in a clear reactivation of these targets as well as of transcribed PCGF6 unique targets devoid of PCGF1/2 co-association. Together, these results suggest that PCGF6 plays a general major role in repression. Furthermore, this occurred at PCGF1/2 co-occupied promoters without loss of H2Aub1. This highlights a marginal role of H2Aub1 in repression that supports previous reports that challenged the role of H2Aub1 in regulating PcG spatiotemporal control of target genes expression during development (Illingworth et al., 2015; Pengelly et al., 2015).

**Figure 4. PCGF1 and PCGF2 Compensate H2Aub1 Deposition at Specific Targets and Are Dispensable for ESC Viability**

(A) Phalloidin immunofluorescence staining in wild-type, *Pcgf1/2/4*, and *Pcgf3/5/6* triple KO mESC. Scale bars correspond to 30 μm.

(B) Principal component analysis of gene expression levels from RNA-seq analysis performed in WT mESCs and in the indicated KOs. Dashed lines enclose experimental replicates.

(C) Volcano plots of –log10 (p value) against log2 fold change representing the differences in gene expression between *Pcgf1/2/4* and *Pcgf3/5/6* KO mESC clones and WT for all protein coding genes (upper panels) and for PCGF1 and PCGF2 targets or PCGF3 and PCGF6 targets, respectively (bottom panels).

(D) Boxplots of normalized ChIP-seq intensity profiles of RING1B, H2AK119ub1 (H2Aub1), SUZ12, and H3K27me3 performed for WT or *Pcgf1*, *Pcgf2/4*, and *Pcgf1/2/4* KO mESC clones, over ±500 bp (or ±4 kb for H2Aub1 and H3K27me3) around the TSS of target genes unique for PCGF3 or PCGF6, or common to PCGF1/2 or PCGF1/2/6.

(E) Genomic snapshots of the ChIP-seq profiles quantified in (D) at selected target gene loci (common or unique, as indicated), performed in WT and the indicated *Pcgf* KO mESC clones.

(F) Boxplots of normalized ChIP-seq intensity profiles of RING1B, H2AK119ub1 (H2Aub1), SUZ12, and H3K27me3 performed in WT or *Pcgf6*, *Pcgf3/5*, and *Pcgf3/5/6* KO mESC clones over ±500 bp (or ±4 kb for H2Aub1 and H3K27me3) around the TSS of unique or common target genes (as indicated).

(G) Genomic snapshots of the ChIP-seq profiles quantified in (F) at selected common or unique target gene loci (as indicated), performed in WT and the indicated *Pcgf* KO mESC clones.

See also Figures S5, S6, and S7 and Tables S5 and S6.

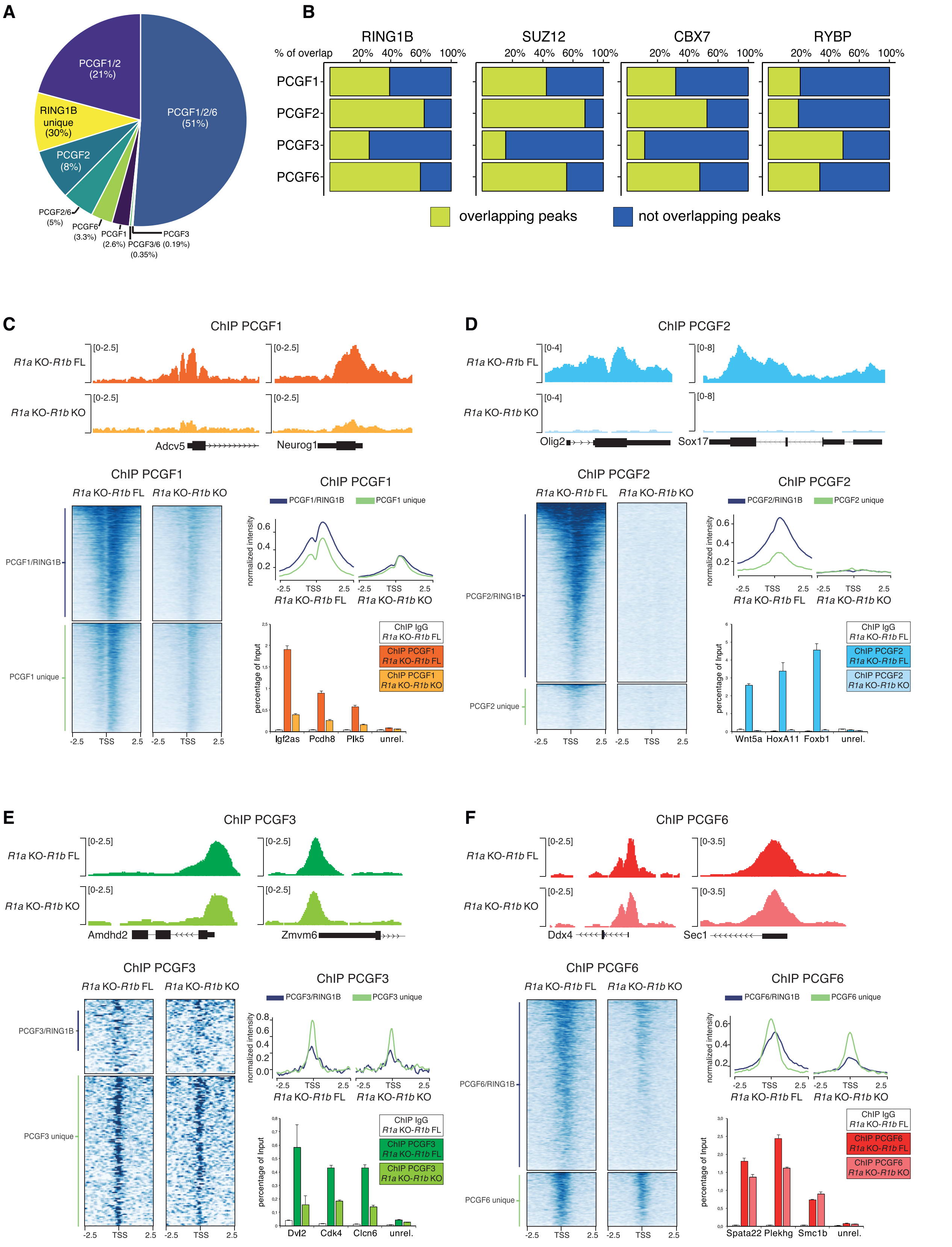


(legend on next page)

## PRC1 Subcomplexes Comprise Repressive or Activating Modules

Our results revealed that the presence of PCGF2 always correlated with full transcriptional repression, regardless of which PCGF protein was co-associated, at target loci involved in developmental processes. This is in line with the co-association of a classical Polycomb signature characterized by abundant H2Aub1 deposition, PRC2 binding, and high H3K27me3 levels. In contrast, PCGF6 unique targets presented a permissive transcriptional state, whereas PCGF3 targets a full transcriptional activity. In addition, our data show that PCGF3 and PCGF6 can both exist in a complex in the absence of RING1A/B association, which correlates with lack of classical PcG chromatin signature. While PCGF6-bound promoters presented a much lower enrichment, PCGF3 sites showed nearly undetectable RING1B and H2Aub1 levels at target loci (enriched for autophagy and lysosomal activity ontologies). However, it is also possible that the high transcriptional status of these targets per se prevents accumulation of repressive marks. Overall, these data showed that the levels of PRC1.2 at target sites correspond well to transcriptional activity, while recruitment of PCGF1, PCGF3, and PCGF6 complexes may be less dependent on the transcriptional status of their target genes. This relates to the passive models of PRC2 recruitment proposed by the Helin and Bernstein laboratories, showing that PRC2 promiscuously binds unmethylated CpG islands until it is excluded by active transcription (Mendenhall et al., 2010; Riising et al., 2014). Indeed, both PRC1.1 and PRC1.2 have (direct or indirect) affinities for CpG-dense regions: KDM2B provides a direct affinity for unmethylated CpGs to PRC1.1 (Farcas et al., 2012), and the H3K27me3 deposited by PRC2 provides a docking site for PRC1.2 (Cao et al., 2002; Fischle et al., 2003; Min et al., 2003). In contrast, the requirement of specific DNA binding activities of PCGF3 and PCGF6 complexes to actively transcribed genes may also suggest distinct biological functions that are different from PCGF1/2.

## PCGF3 and PCGF6 Activity Is Linked to Transcription Activation and Does Not Require RING1A/B Association

While PCGF3 and PCGF5 activated transcription in an artificial assay, PCGF6 behaved as a repressor, in apparent contradiction with its physiological association to actively transcribed targets. Since PCGF6 is recruited to DNA by E-box recognition, we envisioned an antagonistic mechanism between PCGF6 and MYC. MYC shares a large fraction of PCGF6 targets, and, upon loss of PCGF6 functions, a substantial set of transcribed PCGF6 targets increased their transcriptional activity. This suggests that PCGF6 may function as "attenuator" of transcription via yet-uncharacterized mechanisms of regulation that may not involve H2Aub1 deposition. We found that RING1A/B activity was dispensable for both assembly and chromatin recruitment of the PCGF3 and PCGF6 complexes, which could provide insight to a not-yet-identified mechanism regulating the activity of these complexes at target sites in the absence of RING1A/B. Indeed, RING1B recruitment and H2Aub1 deposition were very low at uniquely PCGF6 target sites and were only barely detectable at PCGF3 sites. Based on the crystal structure of the RING1B-PCGF4 (BMI1) heterodimer, PCGF proteins should interact with RING1A/B via dimerization of their respective RING domains (Buchwald et al., 2006). In fact, while this interaction is critical for PRC1.1 and PRC1.2 complexes, it seems to have a poor impact on assembly or recruitment of the PRC1.3 and PRC1.6. Only RYBP association was dependent on RING1A/B, suggesting that RING1A/B-RYBP are recruited to the PCGF3 and PCGF6 complexes as a separate module. How these interactions are regulated, and whether they play any role in the biological function of these complexes, remains to be clarified.

## PCGF6 Is Recruited to DNA by Cooperative Binding to E2F and E-Box Elements

Our data showed that the PCGF6 complex's affinity for DNA comes from the DNA binding activities of its distinct subunits. The complete displacement of PCGF6 from chromatin upon loss of MGA expression agrees with previous reports (Endoh et al., 2017; Stielow et al., 2018) but was a consequence of complex destabilization rather than a loss of DNA interaction. Thus, MGA also plays an important role as a scaffold to assemble the PCGF6 complex, very similar to the role of L3MBTL2 in this same complex (Stielow et al., 2018). Indeed, a minimal C-terminal truncation of the DNA binding domain of MGA can still assemble a normal PRC1.6, only partially reducing its chromatin recruitment. In these conditions, the additional loss of E2F6 further displaced the PCGF6 complex from chromatin, demonstrating that both DNA binding activities are required for efficient PCGF6 complex target recognition.

**Figure 5. PCGF3 and PCGF6 Activities Are Independent of RING1A/B**

(A) Percentage of occupancy of the different PCGF proteins at RING1B-bound promoters.

(B) Percentage of overlap of RING1B, SUZ12, CBX7, and RYBP at the indicated PCGF-bound promoters.

(C) Upper panel: Genomic snapshots of PCGF1 ChIP-seq profiles at selected genomic regions performed in *Ring1A*$^{-/-}$;*Ring1B*$^{fl/fl}$ (*R1A* KO-*R1B* FL) and *Ring1A*$^{-/-}$;*Ring1B*$^{-/-}$ (*R1A* KO-*R1B* KO) mESCs. Bottom left: Heatmap showing the normalized signal of PCGF1 ChIP-seq in *R1A* KO-*R1B* FL and *R1A* KO-*R1B* KO mESC over ±2.5 Kb of PCGF1/RING1B and PCGF1/2/RING1B common target loci, as well as PCGF1 and PCGF1/2 unique target loci. Bottom right: Cumulative quantification of the heatmaps and PCGF1 ChIP-qPCR analysis at selected regions performed in the same mESCs. IgG served as ChIP negative control. ChIP enrichments were normalized to input. Data represent mean ± SEM.

(D) As in (C), for PCGF2 ChIP-seq profiles analyzed at the indicated PCGF2-specific targets.

(E) Upper panel: Genomic snapshots of PCGF3 ChIP-seq profiles at selected genomic regions performed in *R1A* KO-*R1B* FL and *R1A* KO-*R1B* KO mESC. Bottom left: Heatmap showing the normalized signal of PCGF3 ChIP-seq in *R1A* KO-*R1B* FL and *R1A* KO-*R1B* KO mESCs over ±2.5 Kb of PCGF3/RING1B common target loci and PCGF3 unique target loci. Bottom right: Cumulative quantification of the heatmaps and PCGF3 ChIP-qPCR analyses at selected regions performed in the same ESCs. IgG served as ChIP negative control. ChIP enrichments were normalized to input. Data represent mean ± SEM.

(F) As in (E), for PCGF6 ChIP-seq profiles analyzed at the indicated PCGF6-specific targets.

See also Figure S8.

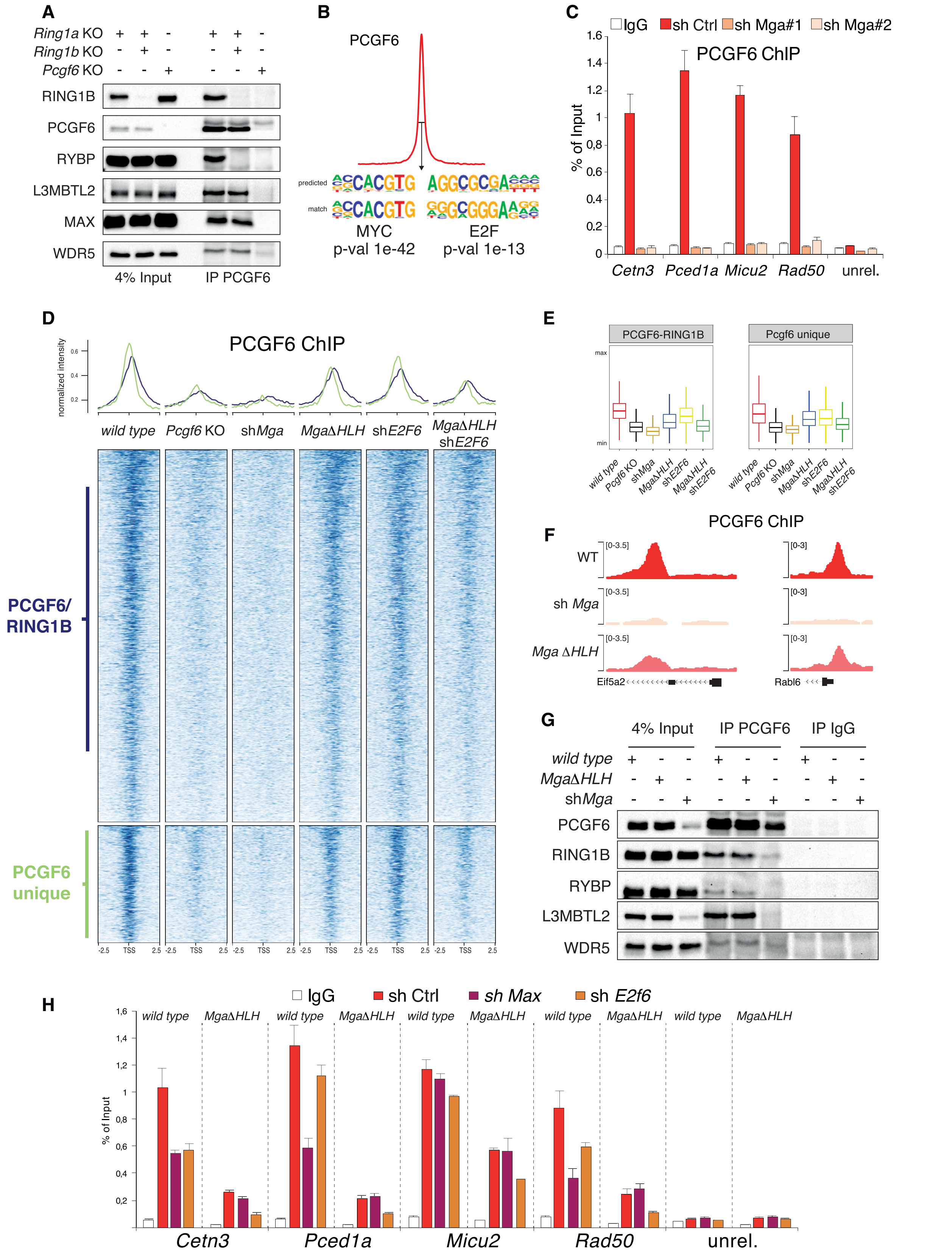


*(legend on next page)*

## PCGF3 Is Recruited to Chromatin by Its Interaction with USF1/2 DNA Binding Transcription Factors

Among the PRC1 subcomplexes, only the PRC1.3 and PRC1.5 complexes do not contain biochemical modules that provide specific affinity for chromatin states (e.g., CBX proteins) and/or DNA elements (e.g., KDM2B, MGA, or E2F6). Through genome-wide location analysis, we identified a strong enrichment for the USF1/2 DNA binding motif that corresponds to a variant E-box that contains a thymidine at the 5′ of the canonical CACGTG E-BOX motif recognized by MYC (TCACGTG). This may explain the moderate but specific enrichment of PCGF3 at some PCGF6 sites. We further demonstrate that USF1 interacts with PCGF3 independently of DNA and that its expression was essential for the recruitment of the PCGF3 complex to all its target sites. USF1/2 is functionally linked with active chromatin states (Pognonec and Roeder, 1991) and, together with the role of the PCGF3 component AUTS2 in mediating P300 recruitment (Gao et al., 2014), directly links the PCGF3 complex to transcriptional activation. It remains unclear how this activatory role is linked with PCGF3/5 mediated regulation of X chromosome inactivation (Almeida et al., 2017). In the future, it would be very interesting to dissect the biochemical basis of PCGF3-USF1 interaction and to know how it is regulated to provide functional specificities.

Altogether, our results provide a first comprehensive analysis of the different PRC1 subcomplexes activities, uncovering their functional specificities that classify them in either repressive or activating modules with defined specific mechanisms of recruitment. Importantly, our data further demonstrate that RING1A/B activity is not essential for the assembly or chromatin recruitment of all PRC1 subcomplexes, suggesting a new potential mechanism of regulation for PCGF3/5 and PCGF6. Based on these latter findings, we speculate that PCGF1 and PCGF2 exert Polycomb-related functions, while PCGF3/5 and PCGF6 activities can also exist in functional forms unrelated to classically defined Polycomb activities.

## STAR★METHODS

Detailed methods are provided in the online version of this paper and include the following:


- KEY RESOURCES TABLE
- CONTACT FOR REAGENT AND RESOURCE SHARING
- EXPERIMENTAL MODEL AND SUBJECT DETAILS
  - Cell culture and cell manipulation
  - Plasmid Generation
  - Protein Purification and Antibody Generation
  - Immunoblot, Immunoprecipitation, and Size-Exclusion Chromatography
  - Sample preparation and mass spectrometry analysis
  - Peptides and proteins identification by database searching
  - ChIP and ChIP-seq
  - ChIP-seq Analysis
  - RNA-seq
  - RNA-seq Analysis
  - Quantitative Real-Time PCR
  - Data availability


## SUPPLEMENTAL INFORMATION

Supplemental Information can be found online at https://doi.org/10.1016/j.molcel.2019.04.002.


## ACKNOWLEDGMENTS

We would like to acknowledge the IEO Genomic and Imaging units for support, Angela Bachi for logistic support with MS, Alessandro Cuomo for the help with MS analyses, Bruno Amati and members of his lab for sharing reagents and discussion, and Veronica Racker (veronica@rakerediting.com) for manuscript editing. We also thank all members of Pasini's laboratory for helpful discussion. D.F.-P. is a PhD student within the European School of Molecular Medicine (SEMM). The work of the Pasini lab was supported by the Italian Association for Cancer Research, AIRC (IG-2014-15798 and IG-2017-20290); by the Italian Ministry of Health (1/GR-2011-02348313); and by the European Recearch Council, ERC (EC-H2020-ERC-CoG-DissectPcG: 725268). A.S. was supported by a fellowship from Italian Foundation for Cancer Research (FIRC) and K.J.F. by a fellowship of the European Institute of Oncology Foundation (FIEO).


## AUTHOR CONTRIBUTIONS

D.P. and A.S. conceived the project. A.S. generated all the ESC lines, the PCGF1-6 antibodies, the samples for RNAseq, the major part of samples for ChIPseq, and several biochemical analyses. S.T. performed the purification and mass spectrometry analyses of the GAL4-PCGF complexes, the Re-ChIP

**Figure 6. PCGF6 Requires Cooperative E2F and E-Box Recognition for Target Recruitment**

(A) Western blot analyses using the indicated antibodies in PCGF6 immunoprecipitations from nuclear extracts prepared from the indicated mESC lines. Input served as loading control.

(B) *De novo* motif discovery analysis performed underneath the summit of PCGF6 peaks. Sequence weight matrixes of predicted compared to match DNA binding motifs are shown together with p values.

(C) PCGF6 ChIP-qPCR analyses on mESC expressing scrambled (sh Ctrl) or *Mga*-specific shRNAs at PCGF6 target and a negative region (neg). IgG served as ChIP negative control. ChIP enrichments are normalized to input. Data represent mean ± SEM.

(D) Normalized intensity profiles and heatmap of PCGF6 binding in WT mESCs or *Pcgf6* KO, a sh*Mga*, *Mga*⊿HLH mutant, sh*E2f6*, and a sh*E2f6* in *Mga*⊿HLH mutant around ±2.5 kb of the TSS of common and unique target loci.

(E) Boxplots of the normalized intensity profiles of ChIP-seq analyses for PCGF6 in WT or *Pcgf6* KO mESCs, a sh*Mga*, *Mga*⊿HLH mutant, sh*E2f6*, and a sh*E2f6* in *Mga*⊿HLH mutant over ±500 bp respective to the TSS of common and unique target loci.

(F) Genomic snapshots of the PCGF6 ChIP-seq profiles at selected genomic regions in WT, sh*Mga*, and *Mga*⊿HLH mESCs.

(G) Western blot analyses using the indicated antibodies in PCGF6 immunoprecipitations from nuclear extracts prepared from WT or sh*Mga*, *Mga*⊿HLH mESCs. IgG served as an unrelated control antibody. Input is shown as loading control.

(H) PCGF6 ChIP-qPCR analysis on WT mESCs and *Mga*⊿HLH mESCs expressing scrambled (Ctrl), *Max,* or *E2F6*-specific shRNAs at PCGF6 target and a negative region (unrel). IgG served as ChIP negative control. ChIP enrichments are normalized to input. Data represent mean ± SEM.

See also Figure S9.

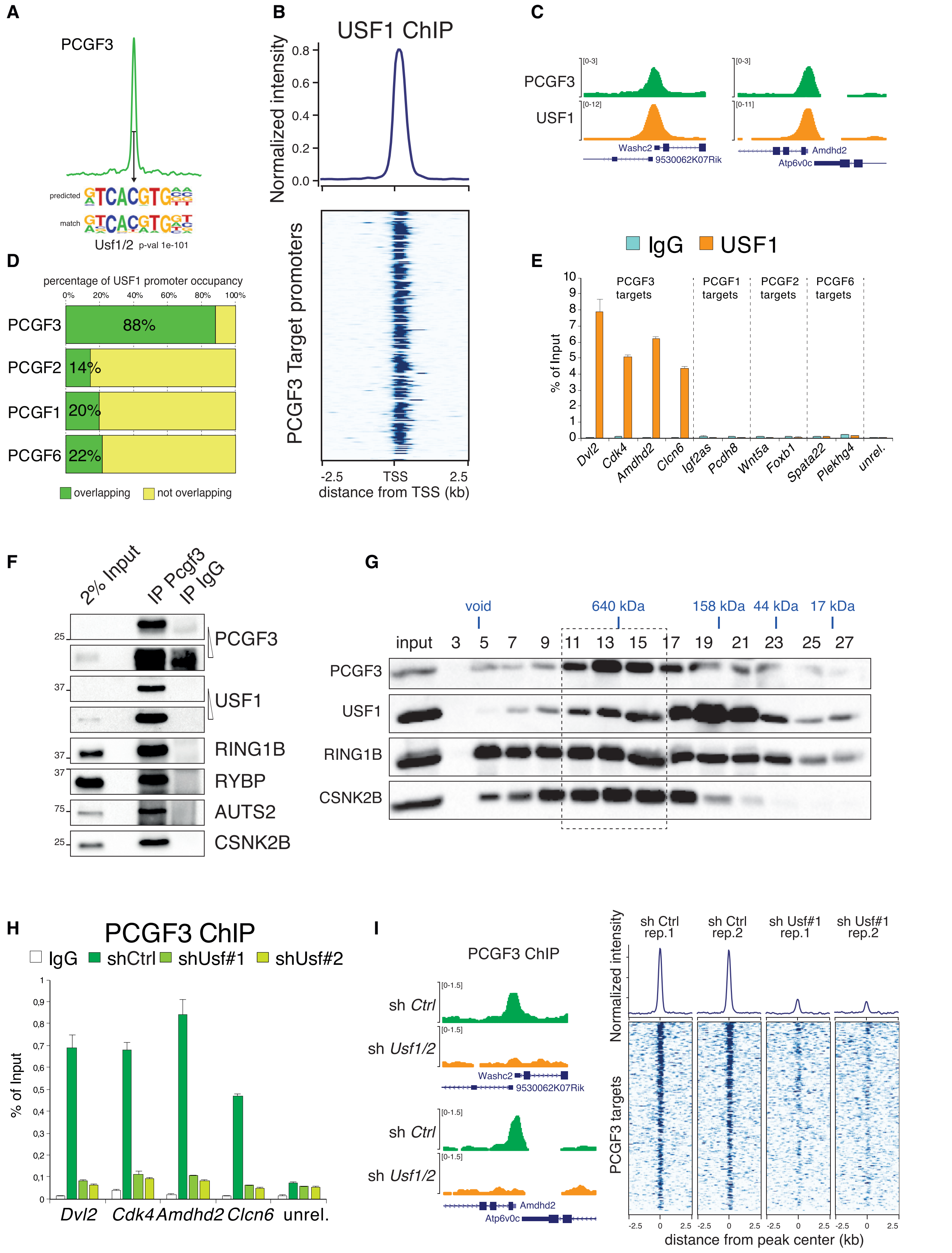


(legend on next page)

analyses, and all staining with ESC; set up the protein ChIP-RX normalization; and provided additional experimental support. K.J.F. prepared the samples for the second PCGF1-6 replicate, generated the PCGF6 GAL4 C-terminal fusion data, and provided additional experimental support. D.F.-P. performed all the computational analyses. M.Z. and E.L. provided support to the experimental work. S.T., M.S., and T.B. performed mass spectrometry analyses for RING1B-PCGF1-6 interactions. D.P., A.S., and D.F.P. made the figures and wrote the manuscript. S.T. and K.J.F. revised the manuscript text.

## DECLARATION OF INTEREST

All authors declare no competing financial interests.

Received: September 28, 2018
Revised: February 22, 2019
Accepted: March 28, 2019
Published: April 24, 2019

## REFERENCES

Akasaka, T., van Lohuizen, M., van der Lugt, N., Mizutani-Koseki, Y., Kanno, M., Taniguchi, M., Vidal, M., Alkema, M., Berns, A., and Koseki, H. (2001). Mice doubly deficient for the Polycomb Group genes Mel18 and Bmi1 reveal synergy and requirement for maintenance but not initiation of Hox gene expression. Development *128*, 1587–1597.

Almeida, M., Pintacuda, G., Masui, O., Koseki, Y., Gdula, M., Cerase, A., Brown, D., Mould, A., Innocent, C., Nakayama, M., et al. (2017). PCGF3/5-PRC1 initiates Polycomb recruitment in X chromosome inactivation. Science *356*, 1081–1084.

Aloia, L., Di Stefano, B., and Di Croce, L. (2013). Polycomb complexes in stem cells and embryonic development. Development *140*, 2525–2534.

Anders, S., Pyl, P.T., and Huber, W. (2015). HTSeq—a Python framework to work with high-throughput sequencing data. Bioinformatics *31*, 166–169.

Avgustinova, A., and Benitah, S.A. (2016). Epigenetic control of adult stem cell function. Nat. Rev. Mol. Cell Biol. *17*, 643–658.

Bernstein, E., Duncan, E.M., Masui, O., Gil, J., Heard, E., and Allis, C.D. (2006). Mouse polycomb proteins bind differentially to methylated histone H3 and RNA and are enriched in facultative heterochromatin. Mol. Cell. Biol. *26*, 2560–2569.

Blackledge, N.P., Farcas, A.M., Kondo, T., King, H.W., McGouran, J.F., Hanssen, L.L., Ito, S., Cooper, S., Kondo, K., Koseki, Y., et al. (2014). Variant PRC1 complex-dependent H2A ubiquitylation drives PRC2 recruitment and polycomb domain formation. Cell *157*, 1445–1459.

Bracken, A.P., and Helin, K. (2009). Polycomb group proteins: navigators of lineage pathways led astray in cancer. Nat. Rev. Cancer *9*, 773–784.

Buchwald, G., van der Stoop, P., Weichenrieder, O., Perrakis, A., van Lohuizen, M., and Sixma, T.K. (2006). Structure and E3-ligase activity of the Ring-Ring complex of polycomb proteins Bmi1 and Ring1b. EMBO J. *25*, 2465–2474.

Cao, R., Wang, L., Wang, H., Xia, L., Erdjument-Bromage, H., Tempst, P., Jones, R.S., and Zhang, Y. (2002). Role of histone H3 lysine 27 methylation in Polycomb-group silencing. Science *298*, 1039–1043.

Chen, H., and Boutros, P.C. (2011). VennDiagram: a package for the generation of highly-customizable Venn and Euler diagrams in R. BMC Bioinformatics *12*, 35.

Consortium, E.P.; ENCODE Project Consortium (2012). An integrated encyclopedia of DNA elements in the human genome. Nature *489*, 57–74.

Cooper, S., Grijzenhout, A., Underwood, E., Ancelin, K., Zhang, T., Nesterova, T.B., Anil-Kirmizitas, B., Bassett, A., Kooistra, S.M., Agger, K., et al. (2016). Jarid2 binds mono-ubiquitylated H2A lysine 119 to mediate crosstalk between Polycomb complexes PRC1 and PRC2. Nat. Commun. *7*, 13661.

de Napoles, M., Mermoud, J.E., Wakao, R., Tang, Y.A., Endoh, M., Appanah, R., Nesterova, T.B., Silva, J., Otte, A.P., Vidal, M., et al. (2004). Polycomb group proteins Ring1A/B link ubiquitylation of histone H2A to heritable gene silencing and X inactivation. Dev. Cell *7*, 663–676.

Di Croce, L., and Helin, K. (2013). Transcriptional regulation by Polycomb group proteins. Nat. Struct. Mol. Biol. *20*, 1147–1155.

Endoh, M., Endo, T.A., Endoh, T., Fujimura, Y., Ohara, O., Toyoda, T., Otte, A.P., Okano, M., Brockdorff, N., Vidal, M., and Koseki, H. (2008). Polycomb group proteins Ring1A/B are functionally linked to the core transcriptional regulatory circuitry to maintain ES cell identity. Development *135*, 1513–1524.

Endoh, M., Endo, T.A., Shinga, J., Hayashi, K., Farcas, A., Ma, K.W., Ito, S., Sharif, J., Endoh, T., Onaga, N., et al. (2017). PCGF6-PRC1 suppresses premature differentiation of mouse embryonic stem cells by regulating germ cell-related genes. eLife *6*, e21064.

Farcas, A.M., Blackledge, N.P., Sudbery, I., Long, H.K., McGouran, J.F., Rose, N.R., Lee, S., Sims, D., Cerase, A., Sheahan, T.W., et al. (2012). KDM2B links the Polycomb Repressive Complex 1 (PRC1) to recognition of CpG islands. eLife *1*, e00205.

Ferrari, K.J., Scelfo, A., Jammula, S., Cuomo, A., Barozzi, I., Stützer, A., Fischle, W., Bonaldi, T., and Pasini, D. (2014). Polycomb-dependent H3K27me1 and H3K27me2 regulate active transcription and enhancer fidelity. Mol. Cell *53*, 49–62.

Fischle, W., Wang, Y., Jacobs, S.A., Kim, Y., Allis, C.D., and Khorasanizadeh, S. (2003). Molecular basis for the discrimination of repressive methyl-lysine marks in histone H3 by Polycomb and HP1 chromodomains. Genes Dev. *17*, 1870–1881.

Gao, Z., Zhang, J., Bonasio, R., Strino, F., Sawai, A., Parisi, F., Kluger, Y., and Reinberg, D. (2012). PCGF homologs, CBX proteins, and RYBP define functionally distinct PRC1 family complexes. Mol. Cell *45*, 344–356.

**Figure 7. Interactions with USF1/2 Mediate PCGF3 Recruitment to Chromatin**

(A) *De novo* motif discovery analysis performed underneath the summit of PCGF3 peaks. Sequence weight matrixes of predicted compared to match DNA binding motifs are shown together with relative p values.

(B) Normalized ChIP-seq intensity profile of USF1 binding over ±2.5 kb regions of PCGF3 target loci.

(C) Genomic snapshots of PCGF3 and USF1 ChIP-seq profiles at PCGF3 targets in WT mESCs.

(D) Relative (percentage) of the different PCGF target genes co-occupied by USF1.

(E) ChIP-qPCR analysis for USF1 in WT mESCs at representative PCGF1, PCGF2, PCGF3, and PCGF6 targets. IgG served as a negative ChIP control. ChIP enrichments are normalized to input. Data represent mean ± SEM.

(F) Wstern blot analyses using the indicated antibodies in PCGF3 immunoprecipitations from nuclear extracts prepared from WT mESCs. Rabbit IgG served as an unrelated control antibody. Inputs are shown as loading control.

(G) Western blot analysis with the indicated antibodies of protein fractions from WT mESC nuclear extracts separated by size-exclusion chromatography. Molecular weights are indicated based on the elution profile of markers in the same conditions.

(H) PCGF3 ChIP-qPCR analysis on mESCs expressing scrambled (shCtrl) or combined *Usf1*- and *Usf2* (sh*Usf*)-specific shRNAs. IgG served as negative ChIP control. ChIP enrichments are normalized to input. Data represent mean ± SEM.

(I) Genomic snapshots of PCGF3 ChIP-seq profiles in scrambled (shCtrl) and combined *Usf1*- and *Usf2* (sh*Usf*)-specific shRNAs expressing mESCs (left panel) and its relative normalized ChIP-seq intensity profiles for two biological replicates (right panels) over a ±2.5 Kb region at PCGF3/RING1B common and PCGF3 unique target loci.

See also Figure S10.

Gao, Z., Lee, P., Stafford, J.M., von Schimmelmann, M., Schaefer, A., and Reinberg, D. (2014). An AUTS2-Polycomb complex activates gene expression in the CNS. Nature *516*, 349–354.

Grant, C.E., Bailey, T.L., and Noble, W.S. (2011). FIMO: scanning for occurrences of a given motif. Bioinformatics *27*, 1017–1018.

Guarnaccia, A.D., and Tansey, W.P. (2018). Moonlighting with WDR5: a cellular multitasker. J. Clin. Med. *7*, e21.

Hauri, S., Comoglio, F., Seimiya, M., Gerstung, M., Glatter, T., Hansen, K., Aebersold, R., Paro, R., Gstaiger, M., and Beisel, C. (2016). A high-density map for navigating the human polycomb complexome. Cell Rep. *17*, 583–595.

Heinz, S., Benner, C., Spann, N., Bertolino, E., Lin, Y.C., Laslo, P., Cheng, J.X., Murre, C., Singh, H., and Glass, C.K. (2010). Simple combinations of lineage-determining transcription factors prime cis-regulatory elements required for macrophage and B cell identities. Mol. Cell *38*, 576–589.

Huang, Y., Zhao, W., Wang, C., Zhu, Y., Liu, M., Tong, H., Xia, Y., Jiang, Q., and Qin, J. (2018). Combinatorial Control of recruitment of a variant PRC1.6 complex in embryonic stem cells. Cell Rep. *22*, 3032–3043.

Hutchins, A.P., Yang, Z., Li, Y., He, F., Fu, X., Wang, X., Li, D., Liu, K., He, J., Wang, Y., et al. (2017). Models of global gene expression define major domains of cell type and tissue identity. Nucleic Acids Res. *45*, 2354–2367.

Illingworth, R.S., Moffat, M., Mann, A.R., Read, D., Hunter, C.J., Pradeepa, M.M., Adams, I.R., and Bickmore, W.A. (2015). The E3 ubiquitin ligase activity of RING1B is not essential for early mouse development. Genes Dev. *29*, 1897–1902.

Kalb, R., Latwiel, S., Baymaz, H.I., Jansen, P.W., Müller, C.W., Vermeulen, M., and Müller, J. (2014). Histone H2A monoubiquitination promotes histone H3 methylation in Polycomb repression. Nat. Struct. Mol. Biol. *21*, 569–571.

Khan, A., Fornes, O., Stigliani, A., Gheorghe, M., Castro-Mondragon, J.A., van der Lee, R., Bessy, A., Chèneby, J., Kulkarni, S.R., Tan, G., et al. (2018). JASPAR 2018: update of the open-access database of transcription factor binding profiles and its web framework. Nucleic Acids Res. *46* (D1), D260–D266.

Kiermaier, A., Gawn, J.M., Desbarats, L., Saffrich, R., Ansorge, W., Farrell, P.J., Eilers, M., and Packham, G. (1999). DNA binding of USF is required for specific E-box dependent gene activation in vivo. Oncogene *18*, 7200–7211.

Kloet, S.L., Makowski, M.M., Baymaz, H.I., van Voorthuijsen, L., Karemaker, I.D., Santanach, A., Jansen, P.W.T.C., Di Croce, L., and Vermeulen, M. (2016). The dynamic interactome and genomic targets of Polycomb complexes during stem-cell differentiation. Nat. Struct. Mol. Biol. *23*, 682–690.

Langmead, B., Trapnell, C., Pop, M., and Salzberg, S.L. (2009). Ultrafast and memory-efficient alignment of short DNA sequences to the human genome. Genome Biol. *10*, R25.

Love, M.I., Huber, W., and Anders, S. (2014). Moderated estimation of fold change and dispersion for RNA-seq data with DESeq2. Genome Biol. *15*, 550.

Margueron, R., Li, G., Sarma, K., Blais, A., Zavadil, J., Woodcock, C.L., Dynlacht, B.D., and Reinberg, D. (2008). Ezh1 and Ezh2 maintain repressive chromatin through different mechanisms. Mol. Cell *32*, 503–518.

Mendenhall, E.M., Koche, R.P., Truong, T., Zhou, V.W., Issac, B., Chi, A.S., Ku, M., and Bernstein, B.E. (2010). GC-rich sequence elements recruit PRC2 in mammalian ES cells. PLoS Genet. *6*, e1001244.

Min, J., Zhang, Y., and Xu, R.M. (2003). Structural basis for specific binding of Polycomb chromodomain to histone H3 methylated at Lys 27. Genes Dev. *17*, 1823–1828.

Morey, L., Aloia, L., Cozzuto, L., Benitah, S.A., and Di Croce, L. (2013). RYBP and Cbx7 define specific biological functions of polycomb complexes in mouse embryonic stem cells. Cell Rep. *3*, 60–69.

Ogawa, H., Ishiguro, K., Gaubatz, S., Livingston, D.M., and Nakatani, Y. (2002). A complex with chromatin modifiers that occupies E2F- and Myc-responsive genes in G0 cells. Science *296*, 1132–1136.

Orkin, S.H., and Hochedlinger, K. (2011). Chromatin connections to pluripotency and cellular reprogramming. Cell *145*, 835–850.

Orlando, D.A., Chen, M.W., Brown, V.E., Solanki, S., Choi, Y.J., Olson, E.R., Fritz, C.C., Bradner, J.E., and Guenther, M.G. (2014). Quantitative ChIP-Seq normalization reveals global modulation of the epigenome. Cell Rep. *9*, 1163–1170.

Pasini, D., and Di Croce, L. (2016). Emerging roles for Polycomb proteins in cancer. Curr. Opin. Genet. Dev. *36*, 50–58.

Pasini, D., Cloos, P.A., Walfridsson, J., Olsson, L., Bukowski, J.P., Johansen, J.V., Bak, M., Tommerup, N., Rappsilber, J., and Helin, K. (2010). JARID2 regulates binding of the Polycomb repressive complex 2 to target genes in ES cells. Nature *464*, 306–310.

Pengelly, A.R., Kalb, R., Finkl, K., and Müller, J. (2015). Transcriptional repression by PRC1 in the absence of H2A monoubiquitylation. Genes Dev. *29*, 1487–1492.

Picelli, S., Björklund, A.K., Reinius, B., Sagasser, S., Winberg, G., and Sandberg, R. (2014). Tn5 transposase and tagmentation procedures for massively scaled sequencing projects. Genome Res. *24*, 2033–2040.

Pognonec, P., and Roeder, R.G. (1991). Recombinant 43-kDa USF binds to DNA and activates transcription in a manner indistinguishable from that of natural 43/44-kDa USF. Mol. Cell. Biol. *11*, 5125–5136.

Posfai, E., Kunzmann, R., Brochard, V., Salvaing, J., Cabuy, E., Roloff, T.C., Liu, Z., Tardat, M., van Lohuizen, M., Vidal, M., et al. (2012). Polycomb function during oogenesis is required for mouse embryonic development. Genes Dev. *26*, 920–932.

Ramírez, F., Ryan, D.P., Grüning, B., Bhardwaj, V., Kilpert, F., Richter, A.S., Heyne, S., Dündar, F., and Manke, T. (2016). deepTools2: a next generation web server for deep-sequencing data analysis. Nucleic Acids Res. *44* (W1), W160–W165.

Riising, E.M., Comet, I., Leblanc, B., Wu, X., Johansen, J.V., and Helin, K. (2014). Gene silencing triggers polycomb repressive complex 2 recruitment to CpG islands genome wide. Mol. Cell *55*, 347–360.

Scelfo, A., Piunti, A., and Pasini, D. (2015). The controversial role of the Polycomb group proteins in transcription and cancer: how much do we not understand Polycomb proteins? FEBS J. *282*, 1703–1722.

Shen, X., Liu, Y., Hsu, Y.J., Fujiwara, Y., Kim, J., Mao, X., Yuan, G.C., and Orkin, S.H. (2008). EZH1 mediates methylation on histone H3 lysine 27 and complements EZH2 in maintaining stem cell identity and executing pluripotency. Mol. Cell *32*, 491–502.

Simon, J.A., and Kingston, R.E. (2013). Occupying chromatin: Polycomb mechanisms for getting to genomic targets, stopping transcriptional traffic, and staying put. Mol. Cell *49*, 808–824.

Stielow, B., Finkernagel, F., Stiewe, T., Nist, A., and Suske, G. (2018). MGA, L3MBTL2 and E2F6 determine genomic binding of the non-canonical Polycomb repressive complex PRC1.6. PLoS Genet. *14*, e1007193.

Trapnell, C., Pachter, L., and Salzberg, S.L. (2009). TopHat: discovering splice junctions with RNA-Seq. Bioinformatics *25*, 1105–1111.

Trojer, P., Cao, A.R., Gao, Z., Li, Y., Zhang, J., Xu, X., Li, G., Losson, R., Erdjument-Bromage, H., Tempst, P., et al. (2011). L3MBTL2 protein acts in concert with PcG protein-mediated monoubiquitination of H2A to establish a repressive chromatin structure. Mol. Cell *42*, 438–450.

Yang, C.S., Chang, K.Y., Dang, J., and Rana, T.M. (2016). Polycomb group protein Pcgf6 acts as a master regulator to maintain embryonic stem cell identity. Sci. Rep. *6*, 26899.

Yu, G., Wang, L.G., Han, Y., and He, Q.Y. (2012). clusterProfiler: an R package for comparing biological themes among gene clusters. OMICS *16*, 284–287.

Zhang, Y., Liu, T., Meyer, C.A., Eeckhoute, J., Johnson, D.S., Bernstein, B.E., Nusbaum, C., Myers, R.M., Brown, M., Li, W., and Liu, X.S. (2008). Model-based analysis of ChIP-Seq (MACS). Genome Biol. *9*, R137.

Zhu, L.J., Gazin, C., Lawson, N.D., Pagès, H., Lin, S.M., Lapointe, D.S., and Green, M.R. (2010). ChIPpeakAnno: a Bioconductor package to annotate ChIP-seq and ChIP-chip data. BMC Bioinformatics *11*, 237.

# STAR★METHODS

## KEY RESOURCES TABLE

| REAGENT or RESOURCE | SOURCE | IDENTIFIER |
|---|---|---|
| Antibodies | | |
| Rabbit anti-Pcgf1 | this study | N/A |
| Rabbit anti-Pcgf2 | this study | N/A |
| Rabbit anti-Pcgf3 | this study | N/A |
| Rabbit anti-Pcgf6 | this study | N/A |
| Rabbit anti-Ring1b | Pasini laboratory | N/A |
| Rabbit anti-Wdr5 | Pasini laboratory | N/A |
| Mouse anti-Vinculin | Pasini laboratory | N/A |
| Rabbit anti-Rybp | Millipore | Cat# AB3637; RRID: AB_2285466 |
| Rabbit anti-Cbx7 | Abcam | Cat# ab21873; RRID: AB_726005 |
| Rabbit anti-Suz12 | Cell Signaling Technology | Cat# 3737S; RRID: AB_2196850 |
| Rabbit anti-H3K27me3 | Cell Signaling Technology | Cat# C36B11; RRID: AB_2616019 |
| Rabbit anti-H3K36me3 | Cell Signaling Technology | Cat# 4909BF; RRID: AB_2616016 |
| Rabbit anti-H3K4me3 | Active Motif | Cat# 39159; RRID: AB_2615077 |
| Rabbit anti-H2AK119ub1 | Cell Signaling Technology | Cat# 8240; RRID: AB_10891618 |
| Rabbit anti-L3mbtl2 | Active Motif | Cat# 39570; RRID: AB_2615062 |
| Rabbit anti-Max | Santa Cruz Biotechnology | Cat# sc-197; RRID: AB_2281783 |
| Mouse anti-Usf1 | Santa Cruz Biotechnology | Cat# sc-101197; RRID: AB_1131108 |
| Rabbit anti-Auts2 | ProteinTech | Cat# 25001-1-AP |
| Rabbit anti-Csnk2b | ProteinTech | Cat# 20234-1-AP |
| VeriBlot IP Detection Reagent (HRP) | Abcam | Cat# ab131366 |
| Rabbit anti-E2F6 | Abcam | Cat# ab53061 |
| Rabbit anti-MGA | Bethyl | Cat# A302-864A |
| Rabbit anti-GAL4 | Santa Cruz Biotechnology | Cat# sc-577 |
| Bacterial and Virus Strains | | |
| STBL3 | Thermo Fisher Scientific | Cat# C737303 |
| TOP10 | Thermo Fisher Scientific | Cat# C404010 |
| BL-21 | Thermo Fisher Scientific | Cat# C600003 |
| Experimental Models: Cell Lines | | |
| Human: HEK293T | ATCC | ATCC CRL-3216 |
| Human: HEK293T 5XGal4TK-Luc-neo Flp-In | Pasini et al., 2010 | N/A |
| Mouse: ES cell line Ring1A−/−;Ring1Bfl/fl; Rosa26::CreERT2 | Endoh et al., 2008 | N/A Strain of origin 129P2/Ola |
| Mouse: ES cell line E14 | Pasini laboratory | N/A Strain of origin 129P2/Ola |
| Mouse: ES cell line E14 Pcgf1 −/− | this study | N/A Strain of origin 129P2/Ola |
| Mouse: ES cell line E14 Pcgf2 −/− | this study | N/A Strain of origin 129P2/Ola |
| Mouse: ES cell line E14 Pcgf3/5−/− | this study | N/A Strain of origin 129P2/Ola |
| Mouse: ES cell line E14 Pcgf6 −/− | this study | N/A Strain of origin 129P2/Ola |
| Mouse: ES cell line E14 Pcgf2/4 −/− | this study | N/A Strain of origin 129P2/Ola |
| Mouse: ES cell line E14 Pcgf1/2/4 −/− | this study | N/A Strain of origin 129P2/Ola |
| Mouse: ES cell line E14 Pcgf3/5/6−/− | this study | N/A Strain of origin 129P2/Ola |
| Drosophila: S2 | ATCC | ATCC CRL-1963 |
| Oligonucleotides | | |
| qPCR primer sets | this study | available upon request |

(*Continued on next page*)

*Continued*

| REAGENT or RESOURCE | SOURCE | IDENTIFIER |
|---|---|---|
| Recombinant DNA | | |
| pX459 2.0 | this study; Table S1 | Addgene #62988 |
| pLKO.1 | this study; Table S1 | Addgene #8453 |
| Chemicals, Peptides, and Recombinant Proteins | | |
| Leukemia inhibitory factor | Pasini laboratory | N/A |
| CHIR99021 | Stemcell technologies | Cat# 72052 |
| PD0325901 | Stemcell technologies | Cat# 72182 |
| Lipofectamine 2000 | Invitrogen | Cat# 11668027 |
| Igepal | Sigma-Aldrich | Cat# I8896 |
| EGS | Sigma-Aldrich | Cat# E3257 |
| Deposited Data | | |
| E14-Cbx7 ChIP-Seq | Morey et al., 2013 | GEO: GSM1041373 |
| E14-Rybp ChIP-Seq | Morey et al., 2013 | GEO: GSM1041375 |
| E14-RNAPolII ChIP-Seq | Riising et al., 2014 | GEO: GSM1399506 |
| E14-Mga ChIP-seq | Stielow et al., 2018 | ArrayExpress: E-MTAB-6007 |
| E14-Kdm2b ChIP-seq | Farcas et al., 2012 | GEO: GSM1003694 |
| E14-H2AK119Ub1 ChIP-Seq | this study | GEO: GSE122715 |
| E14-H3K27m3 ChIP-Seq | this study | GEO: GSE122715 |
| E14-H3K36m3 ChIP-Seq | this study | GEO: GSE122715 |
| E14-H3K4m3 ChIP-Seq | this study | GEO: GSE122715 |
| E14-Pcgf1 ChIP-Seq | this study | GEO: GSE122715 |
| E14-Pcgf2 ChIP-Seq | this study | GEO: GSE122715 |
| E14-Pcgf3 ChIP-Seq | this study | GEO: GSE122715 |
| E14-Pcgf6 ChIP-Seq | this study | GEO: GSE122715 |
| E14-Ring1b ChIP-Seq | this study | GEO: GSE122715 |
| E14-Rybp ChIP-Seq | this study | GEO: GSE122715 |
| E14-Suz12 ChIP-Seq | this study | GEO: GSE122715 |
| E14-Usf1 ChIP-Seq | this study | GEO: GSE122715 |
| E14-Wdr5 ChIP-Seq | this study | GEO: GSE122715 |
| MgadHLH-Pcgf6 ChIP-Seq | this study | GEO: GSE122715 |
| Pcgf124KO-H2AK119Ub1 ChIP-Seq | this study | GEO: GSE122715 |
| Pcgf124KO-H3K27m3 ChIP-Seq | this study | GEO: GSE122715 |
| Pcgf124KO-Ring1b ChIP-Seq | this study | GEO: GSE122715 |
| Pcgf124KO-Suz12 ChIP-Seq | this study | GEO: GSE122715 |
| Pcgf356KO-H2AK119Ub1 ChIP-Seq | this study | GEO: GSE122715 |
| Pcgf356KO-H3K27m3 ChIP-Seq | this study | GEO: GSE122715 |
| Pcgf356KO-Ring1b ChIP-Seq | this study | GEO: GSE122715 |
| Pcgf356KO-Suz12 ChIP-Seq | this study | GEO: GSE122715 |
| Pcgf1KO-H2AK119Ub1 ChIP-Seq | this study | GEO: GSE122715 |
| Pcgf1KO-H3K27m3 ChIP-Seq | this study | GEO: GSE122715 |
| Pcgf1KO-H3K36m3 ChIP-Seq | this study | GEO: GSE122715 |
| Pcgf1KO-H3K4m3 ChIP-Seq | this study | GEO: GSE122715 |
| Pcgf1KO-Pcgf1 ChIP-Seq | this study | GEO: GSE122715 |
| Pcgf1KO-Pcgf2 ChIP-Seq | this study | GEO: GSE122715 |
| Pcgf1KO-Pcgf3 ChIP-Seq | this study | GEO: GSE122715 |
| Pcgf1KO-Pcgf6 ChIP-Seq | this study | GEO: GSE122715 |
| Pcgf1KO-Ring1b ChIP-Seq | this study | GEO: GSE122715 |

(*Continued on next page*)

***Continued***

| REAGENT or RESOURCE | SOURCE | IDENTIFIER |
|---|---|---|
| Pcgf1KO-Suz12 ChIP-Seq | this study | GEO: GSE122715 |
| Pcgf24KO-H2AK119Ub1 ChIP-Seq | this study | GEO: GSE122715 |
| Pcgf24KO-H3K27m3 ChIP-Seq | this study | GEO: GSE122715 |
| Pcgf24KO-H3K36m3 ChIP-Seq | this study | GEO: GSE122715 |
| Pcgf24KO-H3K4m3 ChIP-Seq | this study | GEO: GSE122715 |
| Pcgf24KO-Pcgf1 ChIP-Seq | this study | GEO: GSE122715 |
| Pcgf24KO-Pcgf2 ChIP-Seq | this study | GEO: GSE122715 |
| Pcgf24KO-Pcgf3 ChIP-Seq | this study | GEO: GSE122715 |
| Pcgf24KO-Pcgf6 ChIP-Seq | this study | GEO: GSE122715 |
| Pcgf24KO-Ring1b ChIP-Seq | this study | GEO: GSE122715 |
| Pcgf24KO-Suz12 ChIP-Seq | this study | GEO: GSE122715 |
| Pcgf35KO-H2AK119Ub1 ChIP-Seq | this study | GEO: GSE122715 |
| Pcgf35KO-H3K27m3 ChIP-Seq | this study | GEO: GSE122715 |
| Pcgf35KO-H3K36m3 ChIP-Seq | this study | GEO: GSE122715 |
| Pcgf35KO-H3K4m3 ChIP-Seq | this study | GEO: GSE122715 |
| Pcgf35KO-Pcgf1 ChIP-Seq | this study | GEO: GSE122715 |
| Pcgf35KO-Pcgf2 ChIP-Seq | this study | GEO: GSE122715 |
| Pcgf35KO-Pcgf3 ChIP-Seq | this study | GEO: GSE122715 |
| Pcgf35KO-Pcgf6 ChIP-Seq | this study | GEO: GSE122715 |
| Pcgf35KO-Ring1b ChIP-Seq | this study | GEO: GSE122715 |
| Pcgf35KO-Suz12 ChIP-Seq | this study | GEO: GSE122715 |
| Pcgf6KO-H2AK119Ub1 ChIP-Seq | this study | GEO: GSE122715 |
| Pcgf6KO-H3K27m3 ChIP-Seq | this study | GEO: GSE122715 |
| Pcgf6KO-H3K36m3 ChIP-Seq | this study | GEO: GSE122715 |
| Pcgf6KO-H3K4m3 ChIP-Seq | this study | GEO: GSE122715 |
| Pcgf6KO-Pcgf1 ChIP-Seq | this study | GEO: GSE122715 |
| Pcgf6KO-Pcgf2 ChIP-Seq | this study | GEO: GSE122715 |
| Pcgf6KO-Pcgf3 ChIP-Seq | this study | GEO: GSE122715 |
| Pcgf6KO-Pcgf6 ChIP-Seq | this study | GEO: GSE122715 |
| Pcgf6KO-Ring1b ChIP-Seq | this study | GEO: GSE122715 |
| Pcgf6KO-Suz12 ChIP-Seq | this study | GEO: GSE122715 |
| RingdKO-Pcgf1 ChIP-Seq | this study | GEO: GSE122715 |
| RingdKO-Pcgf2 ChIP-Seq | this study | GEO: GSE122715 |
| RingdKO-Pcgf3 ChIP-Seq | this study | GEO: GSE122715 |
| RingdKO-Pcgf6 ChIP-Seq | this study | GEO: GSE122715 |
| RingFL-Pcgf1 ChIP-Seq | this study | GEO: GSE122715 |
| RingFL-Pcgf2 ChIP-Seq | this study | GEO: GSE122715 |
| RingFL-Pcgf3 ChIP-Seq | this study | GEO: GSE122715 |
| RingFL-Pcgf6 ChIP-Seq | this study | GEO: GSE122715 |
| shCtr-Pcgf3-rep1 ChIP-Seq | this study | GEO: GSE122715 |
| shCtr-Pcgf3-rep2 ChIP-Seq | this study | GEO: GSE122715 |
| shE2F6MgaDHLH-Pcgf6 ChIP-Seq | this study | GEO: GSE122715 |
| shE2F6-Pcgf6 ChIP-Seq | this study | GEO: GSE122715 |
| shMGA-Pcgf6 ChIP-Seq | this study | GEO: GSE122715 |
| shUsf1-Pcgf3 ChIP-Seq | this study | GEO: GSE122715 |
| shUsf2-Pcgf3 ChIP-Seq | this study | GEO: GSE122715 |
| E14-Myc ChIP-Seq | this study | GEO: GSE122715 |

(*Continued on next page*)

*Continued*

| REAGENT or RESOURCE | SOURCE | IDENTIFIER |
|---|---|---|
| Input-E14-293T-DX ChIP-Seq | this study | GEO: GSE122715 |
| Input-E14-293T ChIP-Seq | this study | GEO: GSE122715 |
| Input-E14-293T-Gal4P6 ChIP-Se | this study | GEO: GSE122715 |
| Input-Pcgf1KO-293T-DX ChIP-Se | this study | GEO: GSE122715 |
| Input-Pcgf1KO-293T ChIP-Seq | this study | GEO: GSE122715 |
| Input-Pcgf1KO-293T-Gal4P6 ChI | this study | GEO: GSE122715 |
| Input-Pcgf24KO-293T-DX ChIP-S | this study | GEO: GSE122715 |
| Input-Pcgf24KO-293T ChIP-Seq | this study | GEO: GSE122715 |
| Input-Pcgf24KO-293T-Gal4P6 Ch | this study | GEO: GSE122715 |
| Input-Pcgf35KO-293T-DX ChIP-S | this study | GEO: GSE122715 |
| Input-Pcgf35KO-293T ChIP-Seq | this study | GEO: GSE122715 |
| Input-Pcgf35KO-293T-Gal4P6 Ch | this study | GEO: GSE122715 |
| Input-Pcgf6KO-293T-DX ChIP-Se | this study | GEO: GSE122715 |
| Input-Pcgf6KO-293T ChIP-Seq | this study | GEO: GSE122715 |
| Input-Pcgf6KO-293T-Gal4P6 ChI | this study | GEO: GSE122715 |
| Pcgf1-E14-293T ChIP-Seq | this study | GEO: GSE122715 |
| Pcgf1-Pcgf1KO-293T ChIP-Seq | this study | GEO: GSE122715 |
| Pcgf1-Pcgf24KO-293T ChIP-Seq | this study | GEO: GSE122715 |
| Pcgf1-Pcgf35KO-293T ChIP-Seq | this study | GEO: GSE122715 |
| Pcgf1-Pcgf6KO-293T ChIP-Seq | this study | GEO: GSE122715 |
| Pcgf2-E14-293T ChIP-Seq | this study | GEO: GSE122715 |
| Pcgf2-Pcgf1KO-293T ChIP-Seq | this study | GEO: GSE122715 |
| Pcgf2-Pcgf24KO-293T ChIP-Seq | this study | GEO: GSE122715 |
| Pcgf2-Pcgf35KO-293T ChIP-Seq | this study | GEO: GSE122715 |
| Pcgf2-Pcgf6KO-293T ChIP-Seq | this study | GEO: GSE122715 |
| Pcgf3-E14-rep2 ChIP-Seq | this study | GEO: GSE122715 |
| Pcgf3-Pcgf1KO-rep2 ChIP-Seq | this study | GEO: GSE122715 |
| Pcgf3-Pcgf24KO-rep2 ChIP-Seq | this study | GEO: GSE122715 |
| Pcgf3-Pcgf35KO-rep2 ChIP-Seq | this study | GEO: GSE122715 |
| Pcgf3-Pcgf6KO-rep2 ChIP-Seq | this study | GEO: GSE122715 |
| Pcgf6-E14-293T ChIP-Seq | this study | GEO: GSE122715 |
| Pcgf6-Pcgf1KO-293T ChIP-Seq | this study | GEO: GSE122715 |
| Pcgf6-Pcgf24KO-293T ChIP-Seq | this study | GEO: GSE122715 |
| Pcgf6-Pcgf35KO-293T ChIP-Seq | this study | GEO: GSE122715 |
| Pcgf6-Pcgf6KO-293T ChIP-Seq | this study | GEO: GSE122715 |
| E14-rep1 RNA-Seq | this study | GEO: GSE122715 |
| E14-rep2 RNA-Seq | this study | GEO: GSE122715 |
| P124KO-rep1 RNA-Seq | this study | GEO: GSE122715 |
| P124KO-rep2 RNA-Seq | this study | GEO: GSE122715 |
| P1KO-rep1 RNA-Seq | this study | GEO: GSE122715 |
| P1KO-rep2 RNA-Seq | this study | GEO: GSE122715 |
| P24KO-rep1 RNA-Seq | this study | GEO: GSE122715 |
| P24KO-rep2 RNA-Seq | this study | GEO: GSE122715 |
| P356KO-rep1 RNA-Seq | this study | GEO: GSE122715 |
| P356KO-rep2 RNA-Seq | this study | GEO: GSE122715 |
| P35KO-rep1 RNA-Seq | this study | GEO: GSE122715 |
| P35KO-rep2 RNA-Seq | this study | GEO: GSE122715 |
| P6KO-rep1 RNA-Seq | this study | GEO: GSE122715 |
| P6KO-rep2 RNA-Seq | this study | GEO: GSE122715 |

(*Continued on next page*)

| *Continued* | | |
|---|---|---|
| REAGENT or RESOURCE | SOURCE | IDENTIFIER |
| Software and Algorithms | | |
| Bowtie v1.2.2 | Langmead et al., 2009 | http://bowtie-bio.sourceforge.net/index.shtml |
| PICARD | N/A | http://broadinstitute.github.io/picard |
| MACS2 v2.1.1 | Zhang et al., 2008 | https://github.com/taoliu/MACS |
| ChIPpeakAnno v3.15 | Zhu et al., 2010 | https://bioconductor.org/packages/release/bioc/html/ChIPpeakAnno.html |
| VennDiagram v1.6.20 | Chen and Boutros, 2011 | https://www.rdocumentation.org/packages/VennDiagram |
| ClusterProfiler | Yu et al., 2012 | http://bioconductor.org/packages/release/bioc/html/clusterProfiler.html |
| HOMER | Heinz et al., 2010 | http://homer.ucsd.edu/ |
| DeepTools 2.0 | Ramírez et al., 2016 | https://deeptools.readthedocs.io/en/latest/ |
| TopHat v2.1.1 | Trapnell et al., 2009 | https://ccb.jhu.edu/software/tophat/ |
| HTseq-count v0.8.0 | Anders et al., 2015 | https://www.huber.embl.de/HTSeq |
| FIMO | Grant et al., 2011 | http://meme-suite.org/doc/fimo.html |

## CONTACT FOR REAGENT AND RESOURCE SHARING

Further information and requests for resources and reagents should be directed to and will be fulfilled by the Lead Contact, Diego Pasini (diego.pasini@ieo.it).

## EXPERIMENTAL MODEL AND SUBJECT DETAILS

### Cell culture and cell manipulation

All ESC lines (E14 and derivatives) were grown in a 0.1% gelatin-coated dish in GMEM supplemented with 15% FBS (Euroclone), 2 mM L-glutamine, 100 U/mL penicillin, 0.1 mg/mL streptomycin, 0.1 mM non-essential amino acids, 1 mM sodium pyruvate, 50 μm β-mercaptoethanol (GIBCO), leukemia inhibitory factor, 3 μm GSK3β inhibitor (CHIR99021, STEMCELL Technologies), 1 μm MEK1/2 Inhibitor (PD0325901, STEMCELL Technologies).

For knockdown experiments, cells were transduced with 5 μg/mL polybrene and lentivirus particles delivering pLKO.1 vector expressing specific shRNAs for 16 hr; cells were then puromycin-selected (2 μg/mL) and grown for 72 hr prior harvesting.

To generate stable Pcgf KO cell lines, 10 μg pX459 2.0 plasmids (Addgene) encoding Cas9 and sgRNAs were transfected using Lipofectamine 2000 (Invitrogen), according to the manufacturer's instruction. Puromycin selection was performed for 30 hr at 2 μg/mL, 2000 cells were seeded into a 15-cm dish, and clones were isolated 10 days later. Clones were screened by PCR for genomic DNA, and Western Blot for protein lysates. PCR from positive clones were Sanger-sequenced to confirm genome editing. For double- or triple KO cell lines, single KO clones were subjected to genome editing following the same procedure.

The $Ring1A^{-/-}$;$Ring1B^{fl/fl}$;*Rosa26::CreERT2* conditional mESC line was described previously (Endoh et al., 2008).

Gal4-DBD-Pcgf inducible cell lines were obtained by transfecting the specific pCDNA4/TO-*Gal4-Pcgf* vector into 293TREx containing a stably integrated (Flp-In) 5 × Gal4TK-Luc-neo construct described previously (Pasini et al., 2010). Transfected cells were seeded at limiting dilutions, and isolated clones were screened by western blot for either Gal4 or Pcgf. The Gal4-DBD-*Pcgf* chimera was expressed using by 1 μg/mL doxycycline (Sigma) medium added for 48 hr before collecting for ChIP and luciferase reporter gene assay. For the latter, cells were lysed in passive lysis buffer (Promega) and then quantified with Bradford protein assay (Bio-Rad), and the luciferase assay (Promega) was performed on a GloMax instrument (Promega) according to the manufacturer's instructions.

### Plasmid Generation

Coding sequences for mouse PCGF proteins were amplified from E14 cDNA and cloned into pCR8/Gateway/TOPO/TA (Invitrogen) following the manufacturer's protocol and sequenced verified. Different plasmids were subcloned in the desired compatible vectors by Gateway technology using LR recombinase (Invitrogen). pGEX-4T-*Pcgf* was generated to produce GST fusion proteins; and pCDNA4/TO-Gal4-*Pcgf*, for 293TREx Flp-In Gal4-*Pcgf*. For CRISPR/Cas9 genome editing, sgRNAs were cloned into the pX459 2.0 vector (Addgene). Details for sgRNAs and relative gene target are given in Table S1.

### Protein Purification and Antibody Generation

GST-PCGF fusion proteins were produced in *E. coli* (BL21) according to standard protocols, purified from crude lysates through fast-flow glutathione Sepharose 4B resin (GE Healthcare), and eluted with elution buffer containing 10 mM reduced glutathione. Eluted proteins were dialyzed against PBS, verified by SDS-PAGE Coomassie staining, and used to immunize rabbits (carried out at Eurogentech). Antibodies from crude sera were immunoaffinity purified and tested for specificity.

### Immunoblot, Immunoprecipitation, and Size-Exclusion Chromatography

Western blots were used to analyze lysates obtained with high-salt lysis buffer (20 mM Tris-HCl, pH 7.6, 300 mM NaCl, 10% glycerol, 0.2% [v/v] IGEPAL [Sigma-Aldrich]). For histone modification analyses, lysates were sonicated by Bioruptor (4 pulses at high intensity, 30 s on/off).

For immunoprecipitation experiments, nuclei were purified using nuclear preparation buffer (10 mM Tris, pH 7.6, 2 mM $MgCl_2$, 0.34 M sucrose, 0.25% [v/v] IGEPAL), lysed in high-salt lysis buffer supplemented with EDTA-free cocktail proteases inhibitors (Roche), and incubated for 5 min at 37°C with 25 U/mL benzonase (Merck); 200–500 μg nuclear lysates were incubated with 2 μg of antibody for 2 hr at 4°C, and protein A magnetic beads (Invitrogen) were added for 45 min to recover immunoprecipitated complex. Beads were washed three times in high-salt buffer prior elution with loading sample buffer.

Size-exclusion chromatography of nuclear lysate was performed over a Superose 6 column (GE Healthcare) in 150 mM NaCl-salt buffer mounted on a AKTA chromatography system (GE Healthcare).

For GAL4 immunoprecipitation, 293TRex GAL4-PCGFs nuclear extracts (3 mg) were incubated with 14 μg of anti GAL4 antibody for 3 h at 4 °C and then 40 μl slurry of Sepharose Protein G beads were added for 2 h at 4 °C. IgG were used as negative control. Beads were washed with nuclear extraction buffer and immunocomplexes were eluted in Laemmli buffer and resolved on NuPAGE 4%–12% precast protein gels (Invitrogen).

Size-exclusion chromatography of nuclear lysate was performed over a Superose 6 column (GE Healthcare) in 150 mM NaCl-salt buffer mounted on a AKTA chromatography system (GE Healthcare).

### Sample preparation and mass spectrometry analysis

Proteins from GAL4-PCGFs purification were separated by SDS–PAGE, using 4%–12% NuPAGE Novex Bis–Tris gels (Invitrogen) and NuPAGE MES SDS running buffer (Invitrogen) and then stained with Coomassie Blue using InstantBlue Comassie (Expedeon). Bands from gel were cut and digested with trypsin (Promega) and incubated for 16 h at 37°C for protein digestion. Then, peptide extraction was carried out and the resulting peptides mixture were combined, reduced in volume in a vacuum concentrator, desalted and concentrated using StageTip (Proxeon Biosystems) columns, washed with 30 μL of 0,1% Formic acid (FA) and finally eluted with 40 μL of 80% MeCN in 0,1% FA. The samples were concentrated in vacuum concentrator (Eppendorf concentrator 5301) for 5 min and peptides were dissolved in 7 μL of 0,1% FA. Approximately 5 μL of purified peptide mixture were analyzed on a LC–ESI–MS-MS Q Exactive HF hybrid quadrupole-Orbitrap mass spectrometer (Thermo Fisher Scientific). Full scan MS spectra were acquired in a range of m/z 300–1800.

### Peptides and proteins identification by database searching

Raw data files were analyzed using the peptide search engine Andromeda integrated into the MaxQuant software environment (version 1.5.2.8) with the following parameters: uniprot_cp_hum_2017_01 as protein database, methionine oxidation, Protein N-term Acetylation as variable modifications and carbamidomethylation as fixed, peptide false discovery rate (FDR) 0.01, minimum peptides 2, at least 1 unique, minimum length peptide 6 amino acids. iBAQ intensity values as calculated by MaxQuant were used to estimate relative abundance of proteins. LFQ ratio values are listed in Table S4.

### ChIP and ChIP-seq

ChIP assays were carried out as described previously (Ferrari et al., 2014). Briefly, 1% formaldehyde cross-linked chromatin (1 mg) was sonicated to an average size of 300–600 bp and incubated overnight at 4°C with 1–8 μg of the indicated antibodies. For ChIP-seq, column-purified DNA from a ChIP experiment was quantified using the Qubit dsDNA HS Assay Kit (Thermo Scientific, Q32854) and 10 ng DNA were processed at IEO NGS core unit employing an automated platform (Beckman Coulter) with the Illumina ChIP-seq sample prep kit (IP-102-1001) and multiplexing oligonucleotide kit (PE400-1001). DNA libraries were quality-checked and quantified on an automated sample processing workstation (Caliper Life Sciences) and used for cluster generation and sequencing by the HiSeq 2000 (Illumina) at 50 bp read length. For PCGF1 ChIP, chromatin was crosslinked for 50 min at RT with 2 mM EGS (Sigma) and then for 10 min with 1% formaldehyde. Quantitative ChIP experiments were performed relative to a reference exogenous genome (ChIP-Rx) (Orlando et al., 2014). For histone modifications, a total of 5% of *Drosophila* chromatin from S2 cell line was added to each ChIP reaction, while for PCGF1 and PCGF2 a total of 50% of human chromatin from 293T cell line was used and, for PCGF6 ChIP-Rx, a total of 50% of human chromatin from 293T cell line expressing GAL4-PCGF6 fusion protein was added to each PCGF6 ChIP reaction. Spiked chromatins were sheared at a size of 200–300 bp.

For re-ChIP assays, immunoprecipitated DNA after the first ChIP was eluted with 50 μL of 10 mM DTT for 30 min at 37°C and then diluted 10 × with ChIP buffer, allowing the second ChIP overnight.

## ChIP-seq Analysis

Reads were aligned to the mouse reference genome mm9, or mm9 and dm6 for histone ChIP-Rx, or mm9 and hg38 for PCGF ChIP-Rx samples, using Bowtie v1.2.2 (Langmead et al., 2009) with default parameters without allowing for multi-mapping (–m 1). PCR duplicates were removed using PICARD (http://broadinstitute.github.io/picard/). Ambiguous reads mapping to both mm9 and dm6 or hg38 were discarded. Peaks were called using MACS2 v2.1.1 (Zhang et al., 2008) with parameters -g mm–nomodel -p 1e-10 –B. Due to a higher IP efficiency in PCGF1 rep2, we used a more stringent p value cutoff (10e-30) to make the data comparable to PCGF1 rep1. To avoid false positives, peaks were discarded if they were in both PCGF wild-type samples and the respective PCGF knockout samples. Then, peaks from biological replicates were merged together. A list containing the final PCGF peaks used in the analyses can be found in Table S2. Genomic peak annotation was performed with the R package ChIPpeakAnno v3.15 (Zhu et al., 2010), considering the region ± 2.5 kb around the TSS as the promoter. Scanning of PCGF6 targets for E-box and E2F sites downloaded from JASPAR (Khan et al., 2018) was done using FIMO (Grant et al., 2011) with default parameters. All downstream analyses were performed considering peaks overlapping with promoter regions, unless otherwise specified. Peak lists were then transformed to gene target lists (Table S3), and overlaps were performed using the R package VennDiagram v1.6.20 (Chen and Boutros, 2011). Gene ontology analyses of PCGF targets were performed using the Bioconductor package clusterProfiler (Yu et al., 2012) setting as threshold an adjusted p value and q-value of 0.01. Motif discovery was performed using HOMER (Heinz et al., 2010) with default parameters using as input the regions ± 25 bp around peak summits reported by MACS2.

For heatmap and intensity plot representation of ChIP-seq signal, BigWig files with input signal subtracted were generated using the function bamCompare from deepTools 2.0 (Ramírez et al., 2016) with parameters–ratio subtract –bs 50–extendReads 200. To normalize for differences in sample library size, a scaling factor for each sample was calculated as (1/total mapped reads)*1000000 and was applied during BigWig file generation with the parameter –scaleFactors from bamCompare. For ChIP-Rx samples the scaling factor was calculated as described in (Orlando et al., 2014). Heatmaps were performed using the functions computeMatrix followed by plotHeatmap from deepTools excluding blacklisted regions by ENCODE (Consortium, 2012) (Consortium, 2012). To homogenize the scale of all heatmaps, boxplots and intensity plots, signal intensity was scaled to 0–1 (represented by min-max in the boxplot figures) by applying the formula 1/(P98 – P5) to all matrices generated by computeMatrix. In order to minimize any difference in the IP and library preparation efficiencies between the two batches of biological replicates, the matrices generated from replicates 1 and replicates 2 were averaged and plotted as a single heatmap.

## RNA-seq

RNA-seq was performed with minor modifications according to the SMART-seq2 protocol (Picelli et al., 2014). Briefly, poly-A containing mRNA molecules from 2 μg of total extracted RNA were copied into first-strand cDNA by reverse transcription and template-switching using oligo(dT) primers and an LNA-containing template-switching oligo (TSO); resulting cDNA was pre-amplified, purified, and tagmented with in-house produced Tn5 transposase. cDNA fragments generated after tagmentation were gap-repaired, amplified by PCR, and cleaned to obtain the final cDNA library.

## RNA-seq Analysis

Reads were aligned to the mouse reference genome mm9 using TopHat v2.1.1 (Trapnell et al., 2009) with parameters–no-coverage-search and–library-type fr-unstranded. PCR duplicates were removed using PICARD (http://broadinstitute.github.io/picard/). Gene counts were calculated using HTseq-count v0.8.0 (Anders et al., 2015) with parameters–stranded = no–mode = intersection-nonempty using RefSeq mm9 annotation downloaded from UCSC. Differential expression analyses were performed using the R package DESeq2 v1.20 (Love et al., 2014) using default parameters. Genes with an absolute log2 fold change of 2 and FDR < 0.05 were considered as differentially expressed (Table S5). Gene ontology analysis of differentially expressed genes was performed using the Bioconductor package clusterProfiler (Yu et al., 2012) with default parameters. Full results of gene ontology analyses are provided in Table S6.

## Quantitative Real-Time PCR

Total RNA was extracted from cells using the Quick-RNA kit (Zymo) according to manufacturer's protocol. RNA was used to generate cDNA by reverse transcriptase PCR using the M-MuLV Reverse Transcriptase (Promega). Relative mRNA expression levels were determined using the Go-Taq SYBR Green (Promega) on a Bio-Rad Real-Time PCR System with selected primer pairs. Expression levels were normalized to *Gapdh*, used as a control housekeeping gene, and computed as described previously (Ferrari et al., 2014).

## Data availability

ChIP-seq and RNA-seq datasets are available at GEO database this accession number: GSE122715

**Molecular Cell, Volume *74***

## Supplemental Information

# Functional Landscape of PCGF Proteins Reveals Both RING1A/B-Dependent- and RING1A/B-Independent-Specific Activities

**Andrea Scelfo, Daniel Fernández-Pérez, Simone Tamburri, Marika Zanotti, Elisa Lavarone, Monica Soldi, Tiziana Bonaldi, Karin Johanna Ferrari, and Diego Pasini**

Figure S1

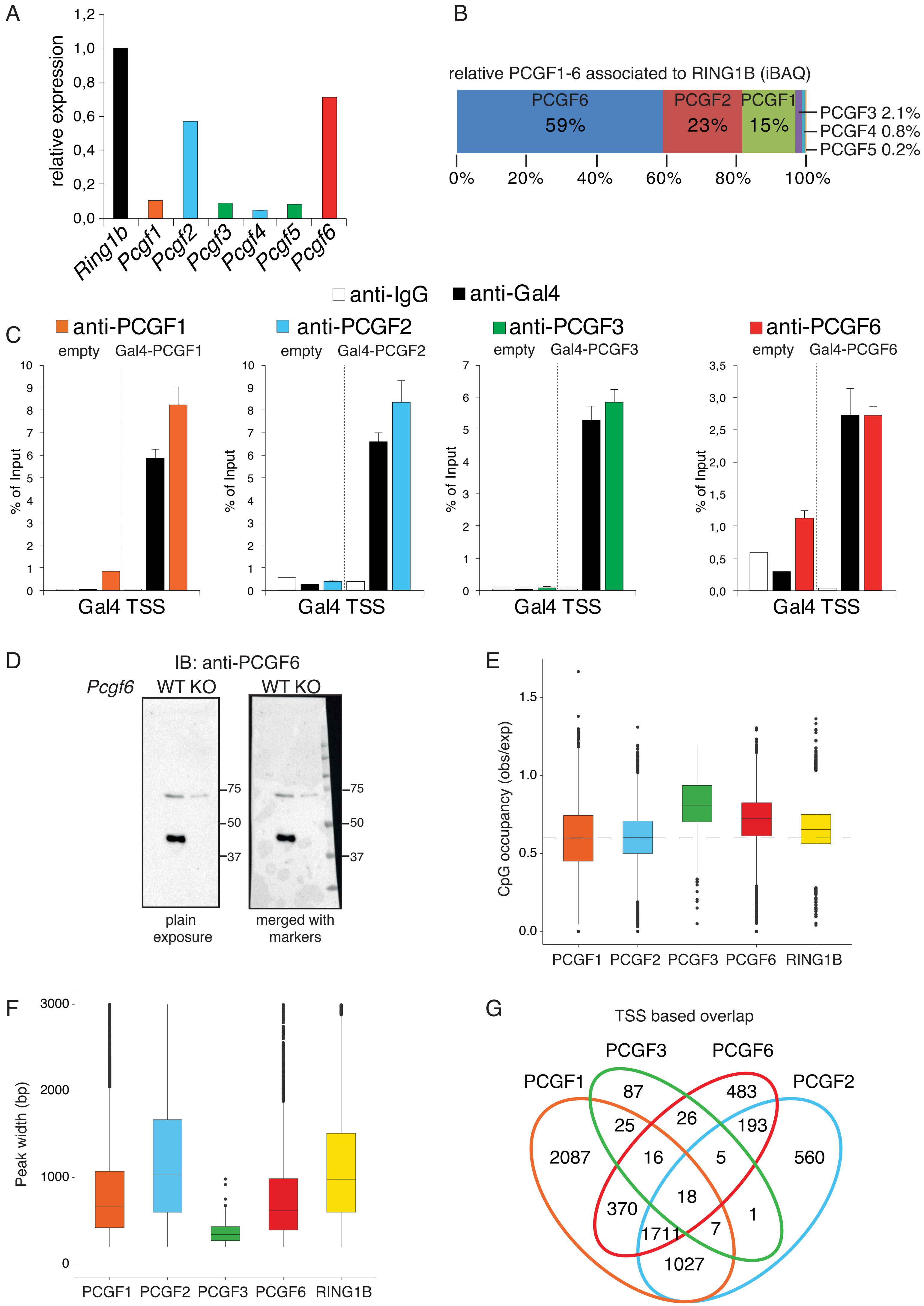
A
relative expression
1,2
1,0
0,8
0,6
0,4
0,2
0,0
Ring1b
Pcgf1
Pcgf2
Pcgf3
Pcgf4
Pcgf5
Pcgf6
B
relative PCGF1-6 associated to RING1B (iBAQ)
PCGF6
59%
PCGF2
23%
PCGF1
15%
PCGF3 2.1%
PCGF4 0.8%
PCGF5 0.2%
0%
20%
40%
60%
80%
100%
anti-IgG
anti-Gal4
C
anti-PCGF1
empty
Gal4-PCGF1
anti-PCGF2
empty
Gal4-PCGF2
anti-PCGF3
empty
Gal4-PCGF3
anti-PCGF6
empty
Gal4-PCGF6
% of Input
Gal4 TSS
D
IB: anti-PCGF6
Pcgf6
WT KO
WT KO
75
50
37
plain exposure
merged with markers
E
CpG occupancy (obs/exp)
1.5
1.0
0.5
0.0
PCGF1
PCGF2
PCGF3
PCGF6
RING1B
F
Peak width (bp)
3000
2000
1000
0
PCGF1
PCGF2
PCGF3
PCGF6
RING1B
G
TSS based overlap
PCGF1
PCGF3
PCGF6
PCGF2
2087
87
483
560
25
26
193
16
5
18
370
1
1711
7
1027

# Figure S2

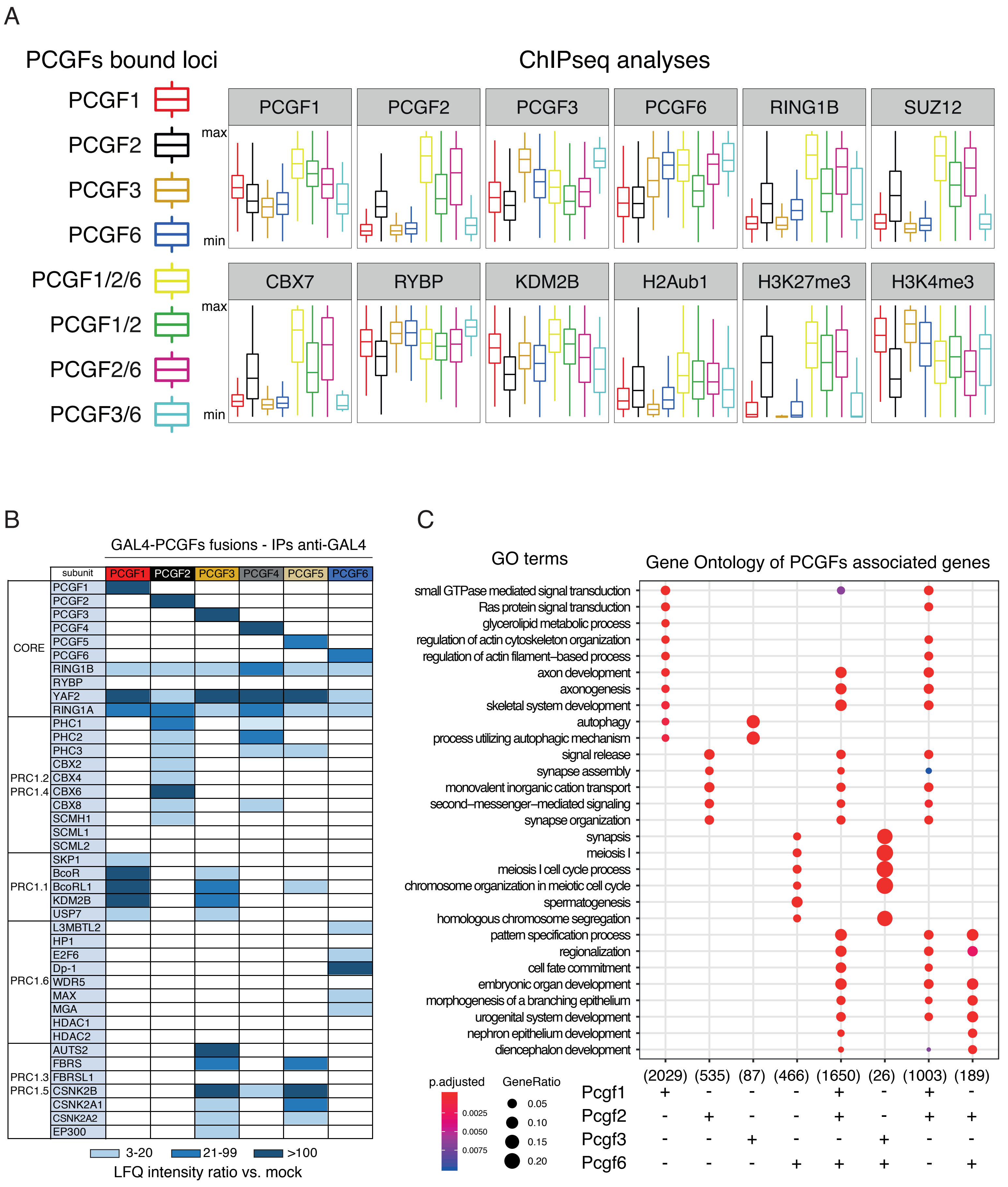
A
PCGFs bound loci
PCGF1
PCGF2
PCGF3
PCGF6
PCGF1/2/6
PCGF1/2
PCGF2/6
PCGF3/6
ChIPseq analyses
max
min
PCGF1
PCGF2
PCGF3
PCGF6
RING1B
SUZ12
CBX7
RYBP
KDM2B
H2Aub1
H3K27me3
H3K4me3
B
GAL4-PCGFs fusions - IPs anti-GAL4
subunit
PCGF1
PCGF2
PCGF3
PCGF4
PCGF5
PCGF6
CORE
PCGF1
PCGF2
PCGF3
PCGF4
PCGF5
PCGF6
RING1B
RYBP
YAF2
RING1A
PRC1.2
PRC1.4
PHC1
PHC2
PHC3
CBX2
CBX4
CBX6
CBX8
SCMH1
SCML1
SCML2
PRC1.1
SKP1
BcoR
BcoRL1
KDM2B
USP7
PRC1.6
L3MBTL2
HP1
E2F6
Dp-1
WDR5
MAX
MGA
HDAC1
HDAC2
PRC1.3
PRC1.5
AUTS2
FBRS
FBRSL1
CSNK2B
CSNK2A1
CSNK2A2
EP300
3-20
21-99
>100
LFQ intensity ratio vs. mock
C
GO terms
Gene Ontology of PCGFs associated genes
small GTPase mediated signal transduction
Ras protein signal transduction
glycerolipid metabolic process
regulation of actin cytoskeleton organization
regulation of actin filament–based process
axon development
axonogenesis
skeletal system development
autophagy
process utilizing autophagic mechanism
signal release
synapse assembly
monovalent inorganic cation transport
second–messenger–mediated signaling
synapse organization
synapsis
meiosis I
meiosis I cell cycle process
chromosome organization in meiotic cell cycle
spermatogenesis
homologous chromosome segregation
pattern specification process
regionalization
cell fate commitment
embryonic organ development
morphogenesis of a branching epithelium
urogenital system development
nephron epithelium development
diencephalon development
(2029) (535) (87) (466) (1650) (26) (1003) (189)
p.adjusted
0.0025
0.0050
0.0075
GeneRatio
0.05
0.10
0.15
0.20
Pcgf1 + - - - + - + -
Pcgf2 - + - - + - + +
Pcgf3 - - + - - + - -
Pcgf6 - - - + + + - +

Figure S3

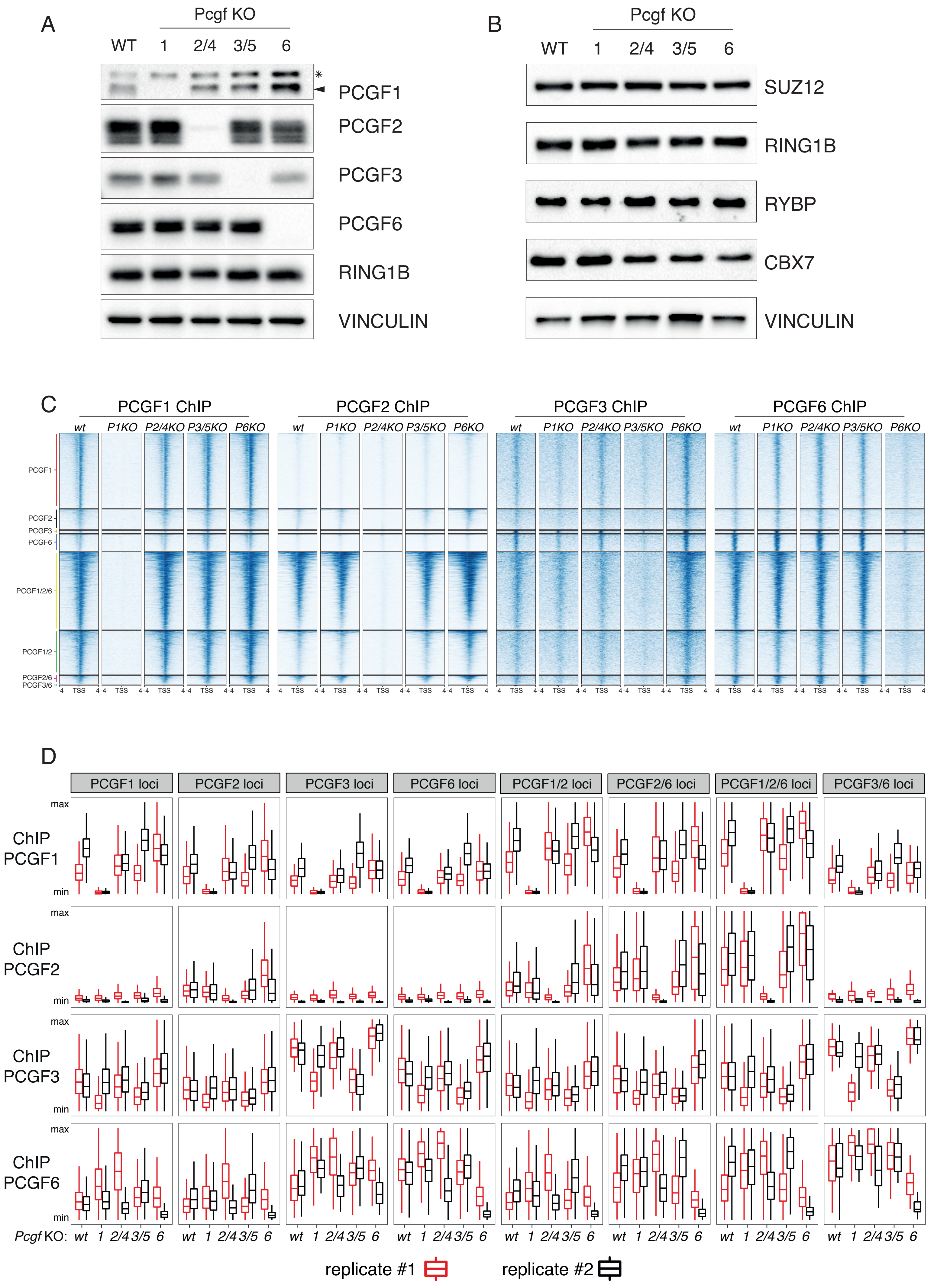
A
Pcgf KO
WT 1 2/4 3/5 6
PCGF1
PCGF2
PCGF3
PCGF6
RING1B
VINCULIN
B
Pcgf KO
WT 1 2/4 3/5 6
SUZ12
RING1B
RYBP
CBX7
VINCULIN
C
PCGF1 ChIP
PCGF2 ChIP
PCGF3 ChIP
PCGF6 ChIP
wt P1KO P2/4KO P3/5KO P6KO
PCGF1
PCGF2
PCGF3
PCGF6
PCGF1/2/6
PCGF1/2
PCGF2/6
PCGF3/6
-4 TSS 4
D
PCGF1 loci
PCGF2 loci
PCGF3 loci
PCGF6 loci
PCGF1/2 loci
PCGF2/6 loci
PCGF1/2/6 loci
PCGF3/6 loci
ChIP PCGF1
ChIP PCGF2
ChIP PCGF3
ChIP PCGF6
max
min
Pcgf KO: wt 1 2/4 3/5 6
replicate #1
replicate #2

# Figure S4

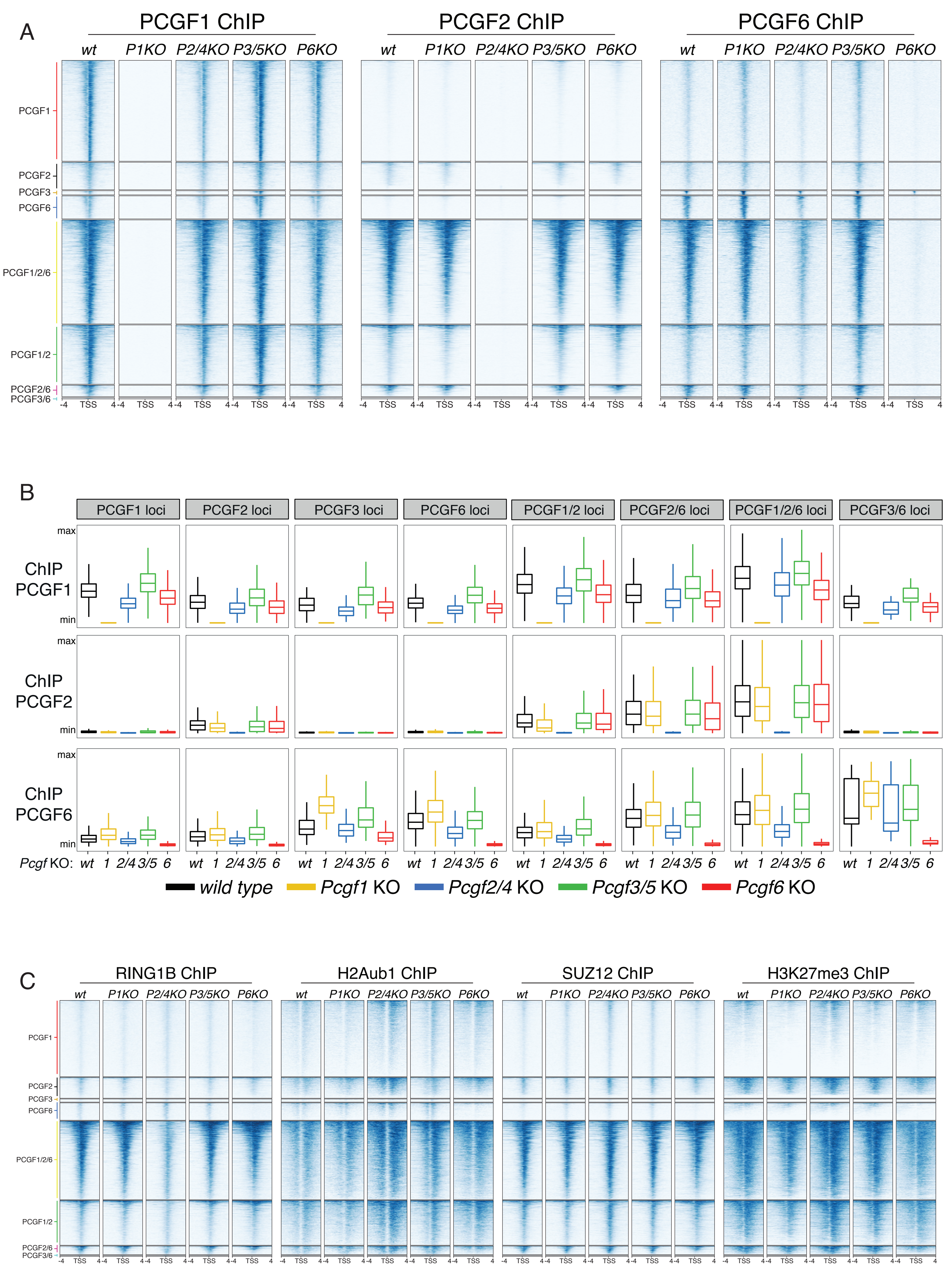

A
PCGF1 ChIP
PCGF2 ChIP
PCGF6 ChIP
wt
P1KO
P2/4KO
P3/5KO
P6KO
PCGF1
PCGF2
PCGF3
PCGF6
PCGF1/2/6
PCGF1/2
PCGF2/6
PCGF3/6
-4
TSS
4
B
PCGF1 loci
PCGF2 loci
PCGF3 loci
PCGF6 loci
PCGF1/2 loci
PCGF2/6 loci
PCGF1/2/6 loci
PCGF3/6 loci
max
min
ChIP PCGF1
ChIP PCGF2
ChIP PCGF6
Pcgf KO: wt 1 2/4 3/5 6
wild type
Pcgf1 KO
Pcgf2/4 KO
Pcgf3/5 KO
Pcgf6 KO
C
RING1B ChIP
H2Aub1 ChIP
SUZ12 ChIP
H3K27me3 ChIP

Figure S5

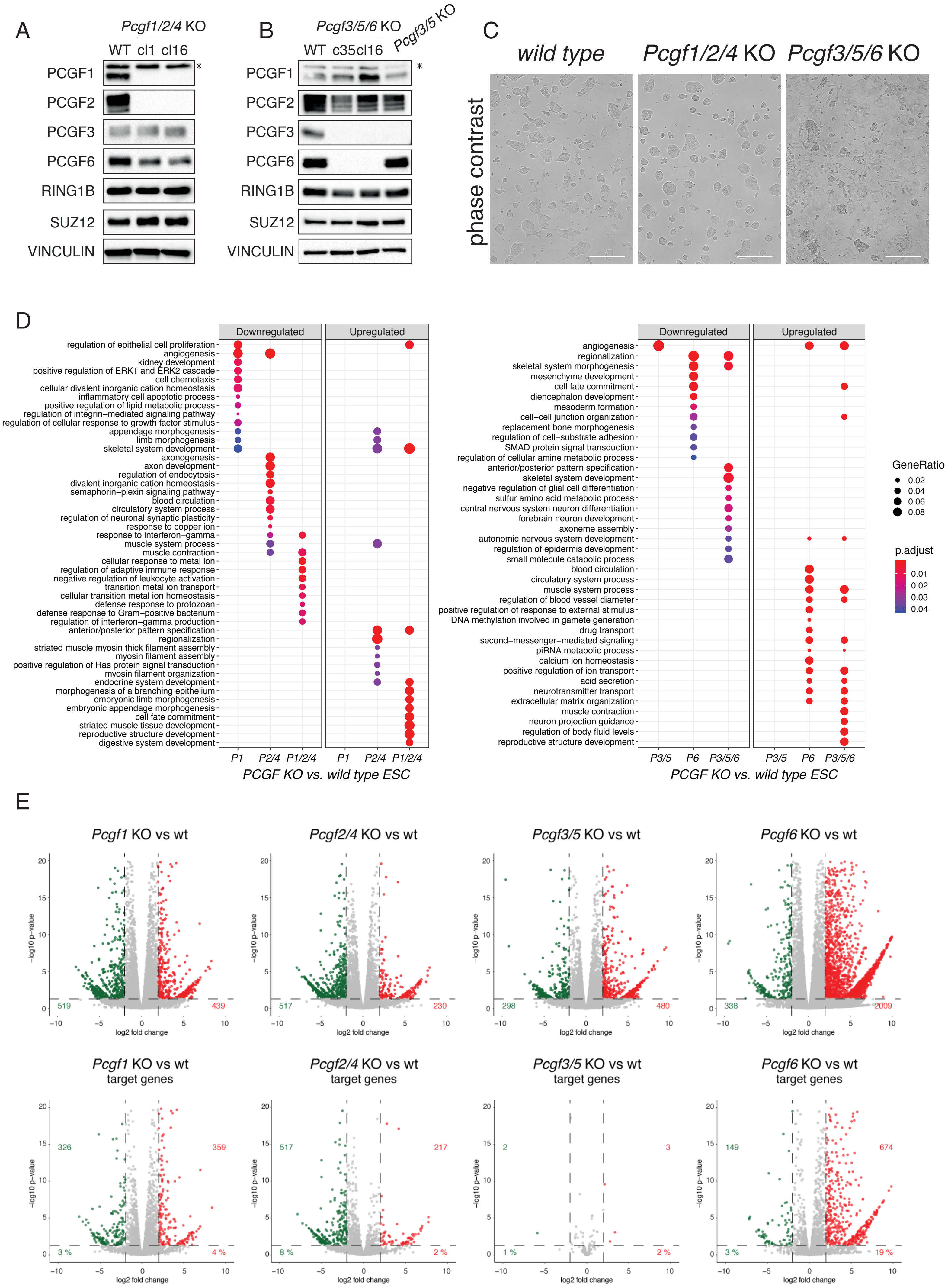
A
Pcgf1/2/4 KO
WT cl1 cl16
PCGF1
PCGF2
PCGF3
PCGF6
RING1B
SUZ12
VINCULIN
*
B
Pcgf3/5/6 KO
Pcgf3/5 KO
WT c35cl16
PCGF1
PCGF2
PCGF3
PCGF6
RING1B
SUZ12
VINCULIN
*
C
wild type
Pcgf1/2/4 KO
Pcgf3/5/6 KO
phase contrast
D
Downregulated
Upregulated
regulation of epithelial cell proliferation
angiogenesis
kidney development
positive regulation of ERK1 and ERK2 cascade
cell chemotaxis
cellular divalent inorganic cation homeostasis
inflammatory cell apoptotic process
positive regulation of lipid metabolic process
regulation of integrin−mediated signaling pathway
regulation of cellular response to growth factor stimulus
appendage morphogenesis
limb morphogenesis
skeletal system development
axonogenesis
axon development
regulation of endocytosis
divalent inorganic cation homeostasis
semaphorin−plexin signaling pathway
blood circulation
circulatory system process
regulation of neuronal synaptic plasticity
response to copper ion
response to interferon−gamma
muscle system process
muscle contraction
cellular response to metal ion
regulation of adaptive immune response
negative regulation of leukocyte activation
transition metal ion transport
cellular transition metal ion homeostasis
defense response to protozoan
defense response to Gram−positive bacterium
regulation of interferon−gamma production
anterior/posterior pattern specification
regionalization
striated muscle myosin thick filament assembly
myosin filament assembly
positive regulation of Ras protein signal transduction
myosin filament organization
endocrine system development
morphogenesis of a branching epithelium
embryonic limb morphogenesis
embryonic appendage morphogenesis
cell fate commitment
striated muscle tissue development
reproductive structure development
digestive system development
P1 P2/4 P1/2/4
PCGF KO vs. wild type ESC
angiogenesis
regionalization
skeletal system morphogenesis
mesenchyme development
cell fate commitment
diencephalon development
mesoderm formation
cell−cell junction organization
replacement bone morphogenesis
regulation of cell−substrate adhesion
SMAD protein signal transduction
regulation of cellular amine metabolic process
anterior/posterior pattern specification
skeletal system development
negative regulation of glial cell differentiation
sulfur amino acid metabolic process
central nervous system neuron differentiation
forebrain neuron development
axoneme assembly
autonomic nervous system development
regulation of epidermis development
small molecule catabolic process
blood circulation
circulatory system process
muscle system process
regulation of blood vessel diameter
positive regulation of response to external stimulus
DNA methylation involved in gamete generation
drug transport
second−messenger−mediated signaling
piRNA metabolic process
calcium ion homeostasis
positive regulation of ion transport
acid secretion
neurotransmitter transport
extracellular matrix organization
muscle contraction
neuron projection guidance
regulation of body fluid levels
reproductive structure development
P3/5 P6 P3/5/6
PCGF KO vs. wild type ESC
GeneRatio
0.02
0.04
0.06
0.08
p.adjust
0.01
0.02
0.03
0.04
E
Pcgf1 KO vs wt
519
439
Pcgf2/4 KO vs wt
517
230
Pcgf3/5 KO vs wt
298
480
Pcgf6 KO vs wt
338
2009
−log10 p−value
log2 fold change
Pcgf1 KO vs wt target genes
326
359
3 %
4 %
Pcgf2/4 KO vs wt target genes
517
217
8 %
2 %
Pcgf3/5 KO vs wt target genes
2
3
1 %
2 %
Pcgf6 KO vs wt target genes
149
674
3 %
19 %

# Figure S6

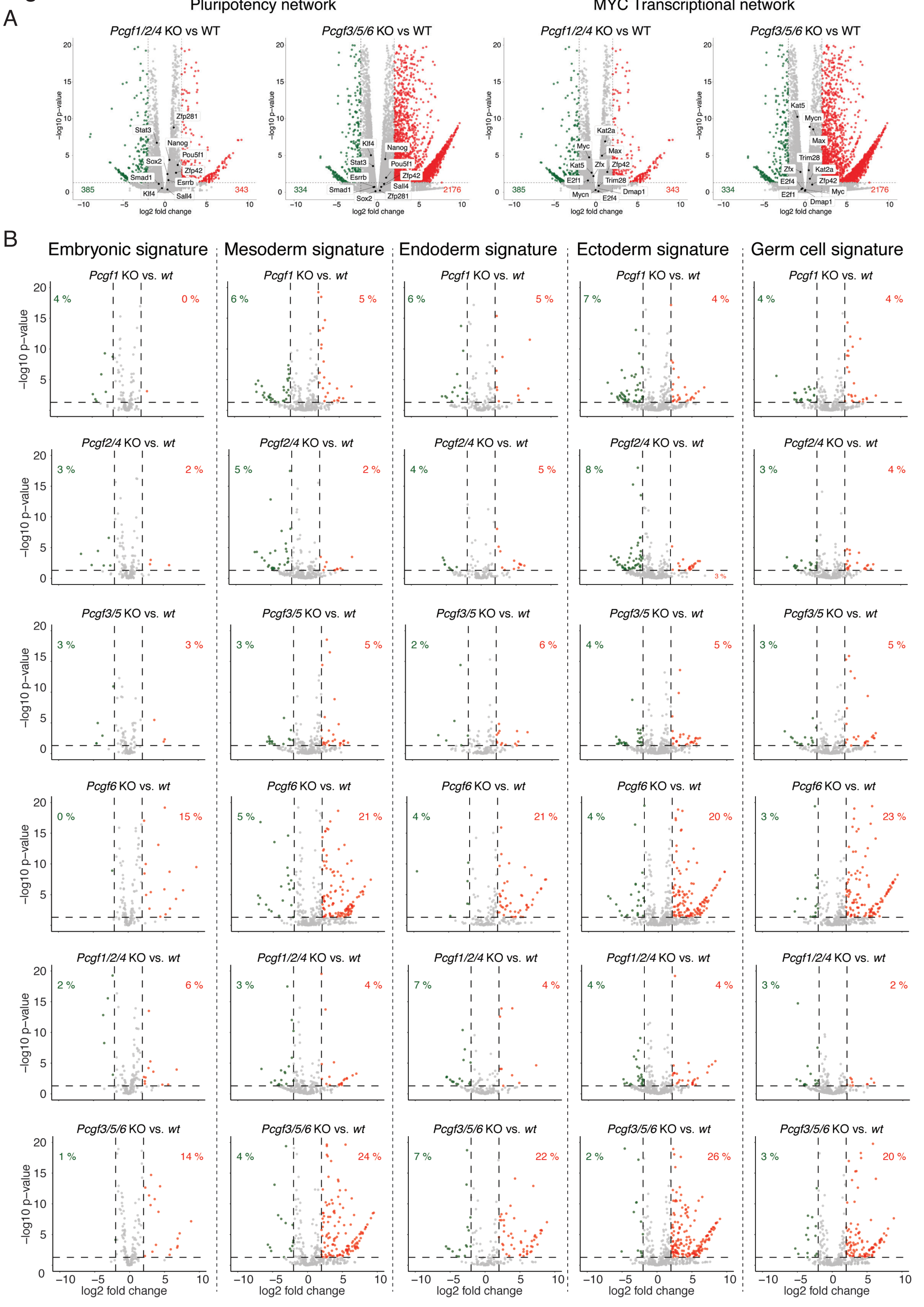

A
Pluripotency network
MYC Transcriptional network
Pcgf1/2/4 KO vs WT
Pcgf3/5/6 KO vs WT
log2 fold change
-log10 p-value
385
343
334
2176
Stat3
Zfp281
Nanog
Pou5f1
Sox2
Zfp42
Smad1
Esrrb
Klf4
Sall4
Kat2a
Myc
Max
Kat5
Zfx
E2f1
Trim28
Mycn
E2f4
Dmap1
B
Embryonic signature
Mesoderm signature
Endoderm signature
Ectoderm signature
Germ cell signature
Pcgf1 KO vs. wt
Pcgf2/4 KO vs. wt
Pcgf3/5 KO vs. wt
Pcgf6 KO vs. wt
Pcgf1/2/4 KO vs. wt
Pcgf3/5/6 KO vs. wt
−log10 p−value
log2 fold change

# Figure S7

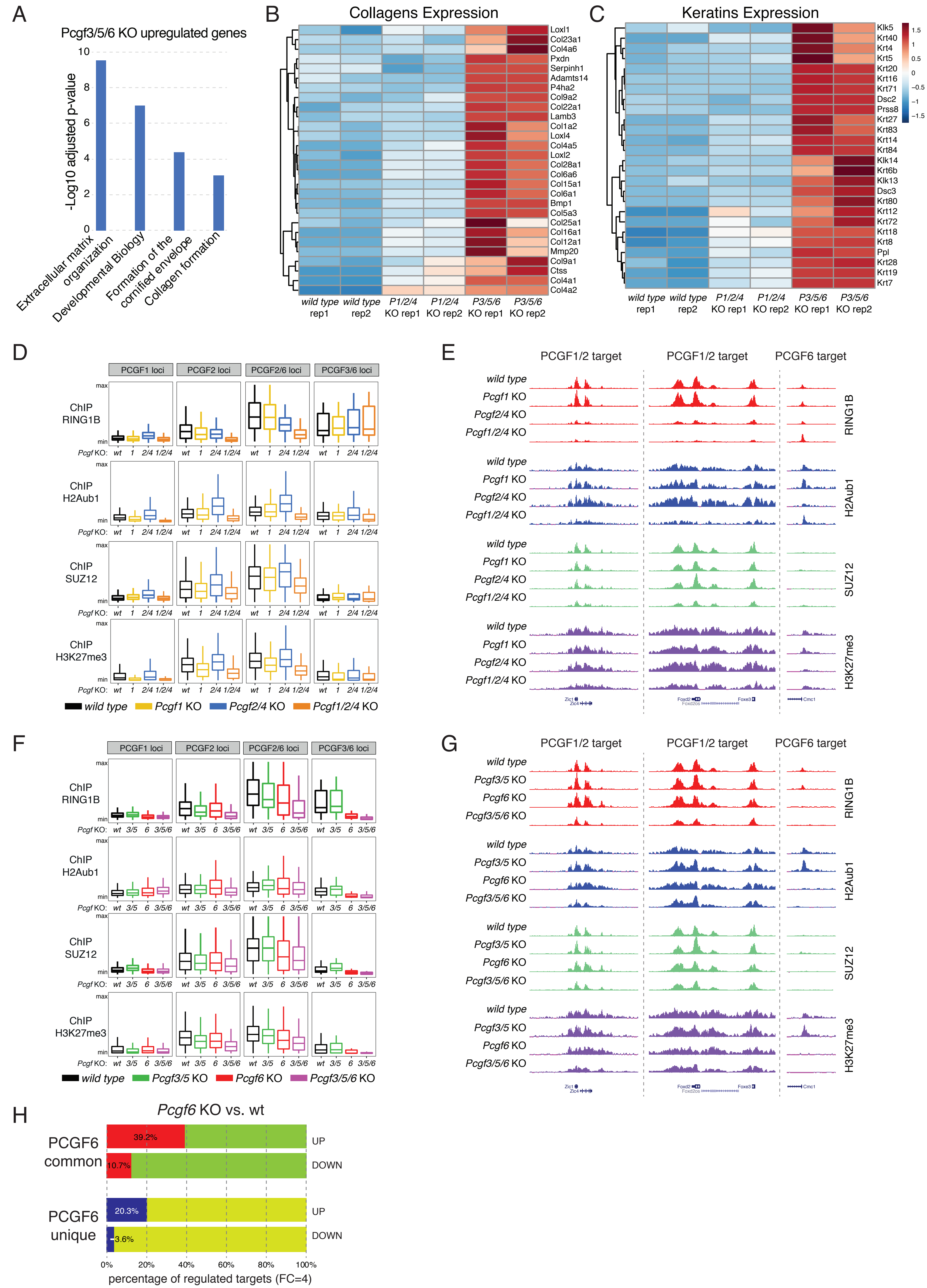

A
Pcgf3/5/6 KO upregulated genes
-Log10 adjusted p-value
Extracellular matrix organization
Developmental Biology
Formation of the cornified envelope
Collagen formation
B
Collagens Expression
Loxl1
Col23a1
Col4a6
Pxdn
Serpinh1
Adamts14
P4ha2
Col9a2
Col22a1
Lamb3
Col1a2
Loxl4
Col4a5
Loxl2
Col28a1
Col6a6
Col15a1
Col6a1
Bmp1
Col5a3
Col25a1
Col16a1
Col12a1
Mmp20
Col9a1
Ctss
Col4a1
Col4a2
wild type rep1
wild type rep2
P1/2/4 KO rep1
P1/2/4 KO rep2
P3/5/6 KO rep1
P3/5/6 KO rep2
C
Keratins Expression
Klk5
Krt40
Krt4
Krt5
Krt20
Krt16
Krt71
Dsc2
Prss8
Krt27
Krt83
Krt14
Krt84
Klk14
Krt6b
Klk13
Dsc3
Krt80
Krt12
Krt72
Krt18
Krt8
Ppl
Krt28
Krt19
Krt7
D
PCGF1 loci
PCGF2 loci
PCGF2/6 loci
PCGF3/6 loci
ChIP RING1B
ChIP H2Aub1
ChIP SUZ12
ChIP H3K27me3
Pcgf KO: wt 1 2/4 1/2/4
wild type
Pcgf1 KO
Pcgf2/4 KO
Pcgf1/2/4 KO
E
PCGF1/2 target
PCGF1/2 target
PCGF6 target
RING1B
H2Aub1
SUZ12
H3K27me3
F
Pcgf KO: wt 3/5 6 3/5/6
Pcgf3/5 KO
Pcgf6 KO
Pcgf3/5/6 KO
G
H
Pcgf6 KO vs. wt
PCGF6 common
PCGF6 unique
UP
DOWN
39.2%
10.7%
20.3%
3.6%
percentage of regulated targets (FC=4)

# Figure S8

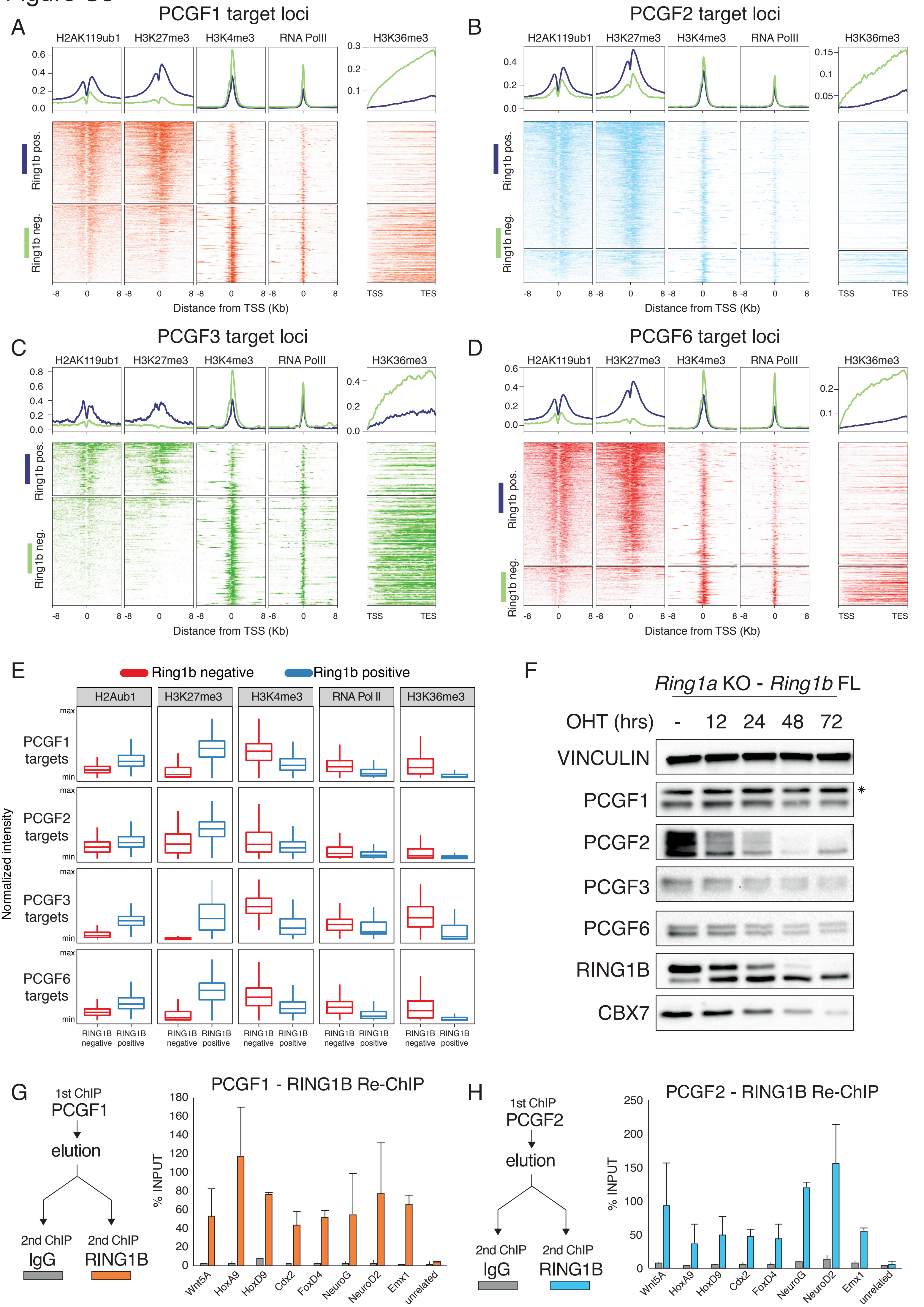
A
PCGF1 target loci
H2AK119ub1
H3K27me3
H3K4me3
RNA PolII
H3K36me3
Ring1b pos.
Ring1b neg.
TSS
TES
Distance from TSS (Kb)
B
PCGF2 target loci
C
PCGF3 target loci
D
PCGF6 target loci
E
Ring1b negative
Ring1b positive
H2Aub1
RNA Pol II
PCGF1 targets
PCGF2 targets
PCGF3 targets
PCGF6 targets
Normalized intensity
max
min
RING1B negative
RING1B positive
F
Ring1a KO - Ring1b FL
OHT (hrs)
-
12
24
48
72
VINCULIN
PCGF1
PCGF2
PCGF3
PCGF6
RING1B
CBX7
*
G
1st ChIP
PCGF1
elution
2nd ChIP
IgG
2nd ChIP
RING1B
PCGF1 - RING1B Re-ChIP
% INPUT
Wnt5A
HoxA9
HoxD9
Cdx2
FoxD4
NeuroG
NeuroD2
Emx1
unrelated
H
PCGF2
PCGF2 - RING1B Re-ChIP

# Figure S9

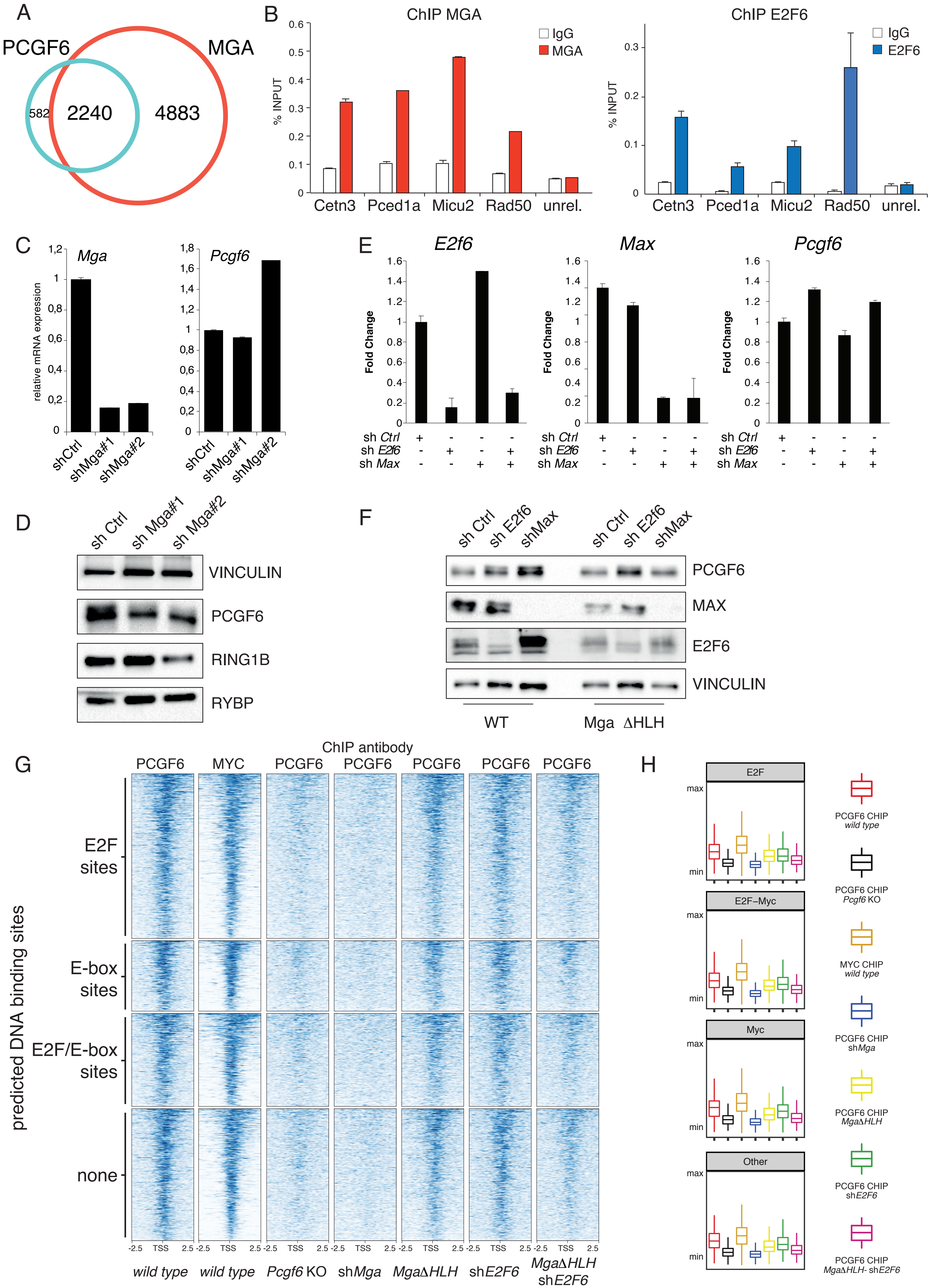

A
PCGF6
MGA
582
2240
4883
B
ChIP MGA
% INPUT
IgG
MGA
Cetn3
Pced1a
Micu2
Rad50
unrel.
ChIP E2F6
E2F6
C
relative mRNA expression
Mga
Pcgf6
shCtrl
shMga#1
shMga#2
E
E2f6
Max
Pcgf6
Fold Change
sh Ctrl
sh E2f6
sh Max
D
sh Ctrl
sh Mga#1
sh Mga#2
VINCULIN
PCGF6
RING1B
RYBP
F
sh Ctrl
sh E2f6
shMax
PCGF6
MAX
E2F6
VINCULIN
WT
Mga ΔHLH
G
ChIP antibody
PCGF6
MYC
predicted DNA binding sites
E2F sites
E-box sites
E2F/E-box sites
none
-2.5
TSS
2.5
wild type
Pcgf6 KO
shMga
MgaΔHLH
shE2F6
MgaΔHLH shE2F6
H
E2F
E2F–Myc
Myc
Other
max
min
PCGF6 CHIP wild type
PCGF6 CHIP Pcgf6 KO
MYC CHIP wild type
PCGF6 CHIP shMga
PCGF6 CHIP MgaΔHLH
PCGF6 CHIP shE2F6
PCGF6 CHIP MgaΔHLH- shE2F6

# Figure S10

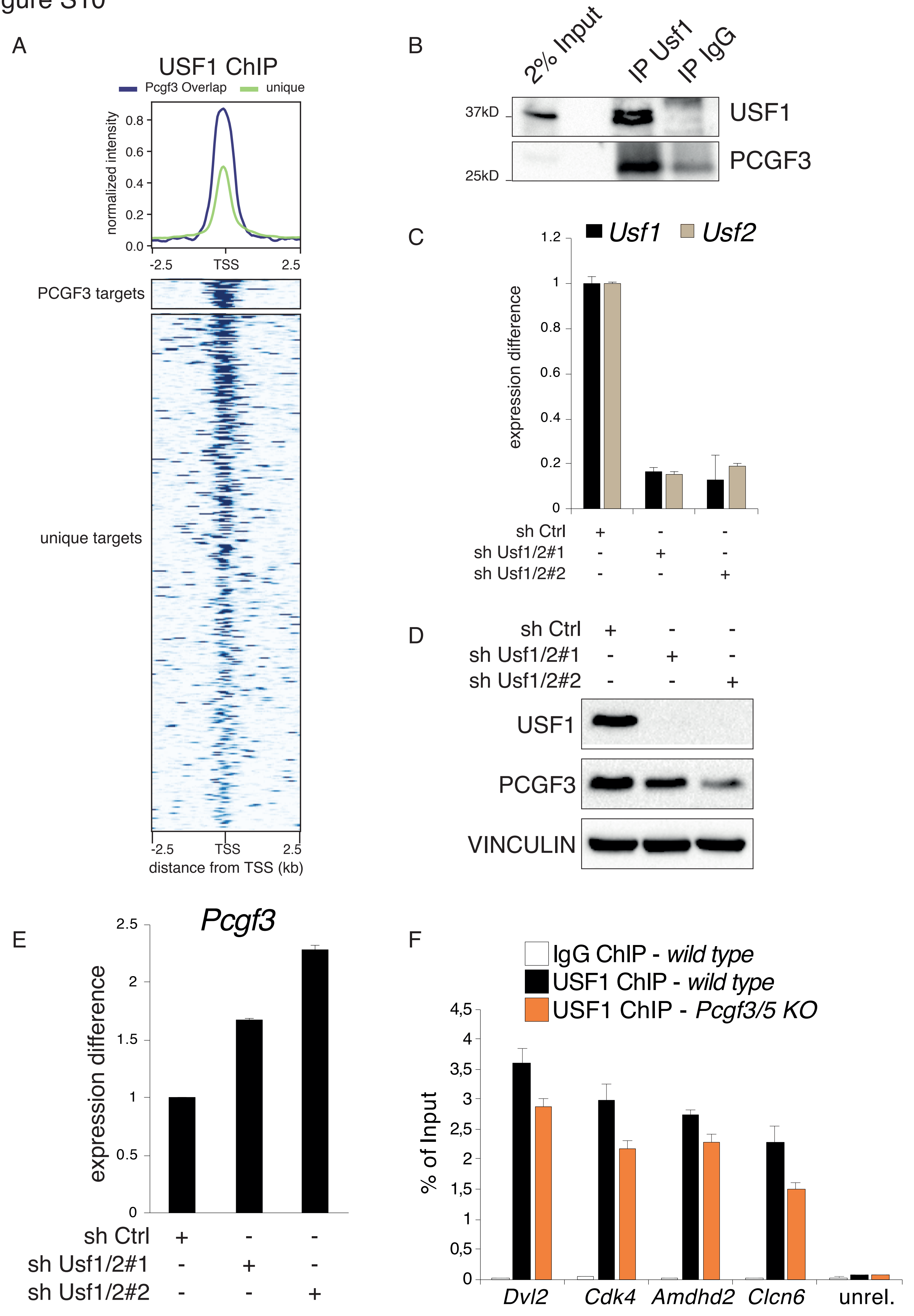
A
USF1 ChIP
Pcgf3 Overlap
unique
normalized intensity
0.8
0.6
0.4
0.2
0.0
-2.5
TSS
2.5
PCGF3 targets
unique targets
distance from TSS (kb)
B
2% Input
IP Usf1
IP IgG
37kD
25kD
USF1
PCGF3
C
Usf1
Usf2
expression difference
1.2
1
0.8
0.6
0.4
0.2
0
sh Ctrl + - -
sh Usf1/2#1 - + -
sh Usf1/2#2 - - +
D
sh Ctrl + - -
sh Usf1/2#1 - + -
sh Usf1/2#2 - - +
USF1
PCGF3
VINCULIN
E
Pcgf3
expression difference
2.5
2
1.5
1
0.5
0
sh Ctrl + - -
sh Usf1/2#1 - + -
sh Usf1/2#2 - - +
F
IgG ChIP - wild type
USF1 ChIP - wild type
USF1 ChIP - Pcgf3/5 KO
% of Input
4,5
4
3,5
3
2,5
2
1,5
1
0,5
0
Dvl2
Cdk4
Amdhd2
Clcn6
unrel.

## Supplemental Figure Legends

**Figure S1. Genome-wide PCGF Binding Specificity. Related to Figure 1**

(A) RT-qPCR analysis showing the mRNA expression levels of the indicated *Pcgf* genes in WT mESC relative to *Ring1b* expression. *Gapdh* served as normalizing expression control. Data represent mean ± SEM.

(B) Relative abundance based on intensity Based Absolute Quantification (iBAQ) values of PCGF peptides identified in RING1B-Flag-bio tandem purifications coupled to mass-spectrometry experiment.

(C) ChIP-qPCR analysis at the *Gal4*-TK-Luciferase promoter of ChIP experiments in the indicated 293Trex clones expressing inducible Gal4 (empty) or Gal4-PCGF fusion proteins, comparing independently the efficiency of the GAL4 to the PCGF1, PCGF2, PCGF3 or PCGF6 antibodies. Rabbit IgG served as a negative control. ChIP enrichments are presented as percentage of input. Data represent mean ± SEM.

(D) Uncropped western blot images of immunoblotting with PCGF6 antibody on WT and *Pcgf6* KO mESC extracts.

(E) Observed/expected ratio of CpG dinucleotides contained within the peaks of the indicated ChIP-seq analyses. Ratio of 0.6 (black-dashed line) or higher is associated with the presence of CpG islands.

(F) Average width of the peaks identified in the indicated ChIP-seq profiles.

(G) Venn diagram showing the overlap between the target genes for the different indicated PCGF proteins.

**Figure S2. Genomic and Proteomic Landscape of PRC1 Family Complexes. Related to Figure 2**

(A) Boxplots of the input subtracted normalized intensity profiles of ChIP-seq analyses for the indicated proteins and histone modifications over ±4 kb of the indicated loci stratified for PCGF co-occupancy in wild-type WT mESCs. CBX7 and RYBP datasets from mESCs were obtained from (Morey et al., 2013) and KDM2B dataset from (Farcas et al., 2012).

(B) Heatmap of PRC1-associated complexes. The GAL4-PCGF (1-6) PRC1 subunits indicated at the top of the table were immunoprecipitated and the recovered proteins were subjected to Liquid Chromatography-Mass Spectrometry (LC-MS). The LFQ (Label Free quantification) ratio is color-coded and displayed as a heatmap with color intensity representing the degree of enrichment obtained in every specific immunoprecipitation. Proteins are grouped in accordance to which GAL4-PCGF they associate with.

(C) Gene ontology analysis for the indicated PCGF unique and co-occupied target genes. The most represented categories are highlighted. Dot size is proportional to the number of genes corresponding to that gene ontology category, color scale indicates statistical significance (adjusted p-value < 0.01 and q-value < 0.01).

**Figure S3. Functional Cross-talk Between Different PCGF Proteins. Related to Figure 3**

(A, B) Western blot analyses with the indicated antibodies of protein lysates from WT and indicated *Pcgf* KO mESC clones. Vinculin served as a loading control. *, unspecific signal.

(C) Heatmaps of the input subtracted normalized intensity profiles of ChIP-seq analyses for PCGF1, PCGF2, PCGF3, or PCGF6 performed in WT mESCs, *Pcgf1*, *Pcgf2/4*, *Pcgf3/5*, or *Pcgf6* KO ESC clones. The regions plotted correspond to ±4 kb around TSS of unique and co-occupied target genes, as indicated.

(D) Boxplots of the input subtracted normalized intensity profiles from two distinct biological replicates of ChIP-seq analyses for PCGF1, PCGF2, PCGF3, or PCGF6 performed in WT, *Pcgf1*, *Pcgf2/4*, *Pcgf3/5*, or *Pcgf6* KO mESC clones. Signal enrichment was calculated over ±500 bp around the TSS of the indicated loci stratified for PCGF co-occupancy in WT mESCs.

**Figure S4: Chip-Seq Normalization by Spike-in RX Human. Related to Figure 3**

(A) Heatmaps of the spike-in input subtracted normalized intensity profiles of ChIP-seq analyses for PCGF1, PCGF2, or PCGF6 performed in WT mESCs, *Pcgf1*, *Pcgf2/4*, *Pcgf3/5*, or *Pcgf6* KO ESC clones. The regions plotted correspond to ±4 kb around TSS of unique and co-occupied target genes, as indicated.

(B) Boxplots of the spike-in input subtracted normalized intensity profiles of ChIP-seq analyses for PCGF1, PCGF2, or PCGF6 performed in WT mESCs, *Pcgf1*, *Pcgf2/4*, *Pcgf3/5*, or *Pcgf6* KO ESC clones. The regions plotted correspond to ±500 bp around TSS of unique and co-occupied target genes, as indicated.

(C) Heatmaps of the input subtracted normalized intensity profiles of ChIP-seq analyses for RING1B, H2Aub1, SUZ12, or H3K27me3 performed in WT mESCs, *Pcgf1*, *Pcgf2/4*, *Pcgf3/5*, or *Pcgf6* KO ESC clones. The regions plotted correspond to ±4 kb around TSS of unique and co-occupied target genes, as indicated.

**Figure S5. Phenotypic and Transcriptomic Analyses of *Pcgf* KOs mESC. Related to Figure 4.**

(A, B) Western blot analyses with the indicated antibodies of protein lysates from WT and indicated *Pcgf* KO mESC clones. Vinculin served as a loading control. *, unspecific signal.

(C) Representative phase-contrast field of WT, *Pcgf1/2/4* and *Pcgf3/5/6* triple KO mESCs. Scale bars correspond to 200 μm.

(D) Gene ontology analysis for the differentially expressed genes in the indicated *Pcgf* KOs. The most represented categories are highlighted. Dot size is proportional to the number of genes corresponding to that Gene ontology category, color scale indicates statistical significance (adjusted p-value < 0.01 and q-value < 0.01).

(E) Volcano plots of –log10 (P-value) against log2 fold change representing the differences in gene expression between *Pcgf1*, *Pcgf2/4*, *Pcgf3/5*, or *Pcgf6* KO mESC clones and WT for all protein coding genes (upper panels) and for PCGF1, PCGF2, PCGF3 and PCGF6 targets, respectively (bottom panels).

**Figure S6. Transcriptomic Analysis of Pluripotency and Differentiation Signatures in *Pcgf* KOs mESC. Related to Figure 4.**

(A) Volcano plots of –log10 (P-value) against log2 fold change representing pluripotency related genes, MYC transcriptional network genes from RNA-seq analyses performed in WT and *Pcgf1/2/4* or *Pcgf3/5/6* triple KO mESCs.

(B) Volcano plots of –log10 (P-value) against log2 fold change of genes from multiple embryonic layers signatures obtained from (Hutchins et al., 2017) from RNA-seq analyses performed in WT and *Pcgf1, Pcgf2/4, Pcgf3/5, Pcgf6, Pcgf1/2/4* or *Pcgf3/5/6* KO mESCs.

**Figure S7. Analysis of Polycomb Signature at PCGF Targets *i*n Triple KO mESCs. Related to Figure 4.**

(A) Gene ontology enrichments based on adjusted p-values ($-\log_{10}$ p) of genes upregulated in *Pcgf3/5/6* triple KO mESCs.

(B) Z-score expression heatmaps for collagen related genes from RNAseq analyses performed in WT, *Pcgf1/2/4* or *Pcgf3/5/6* triple KO mESCs

(C) As in (B) for several indicated keratins related genes.

(D) Boxplots of the input subtracted normalized ChIP-seq intensity profiles of RING1B, H2AK119ub1 (H2Aub1), SUZ12, H3K27me3 performed in WT and *Pcgf1*, *Pcgf2/4* and *Pcgf1/2/4* KO mESC clones. Signal enrichment is calculated over ±500 bp (±4 kb for H2Aub1 and H3K27me3) around the TSS of the indicated group of PCGF target genes.

(E) Genomic snapshots of the ChIP-seq profiles quantified in (A) at selected PCGF1/2 common and PCGF6 unique target gene loci, performed in WT and in the indicated KO mESCs.

(F) Boxplots of the input subtracted normalized ChIP-seq intensity profiles of RING1B, H2AK119ub1 (H2Aub1), SUZ12, H3K27me3 performed in WT, *Pcgf6*, *Pcgf3/5*, and *Pcgf3/5/6* KO mESC clones. Signal enrichment is calculated over ±500 bp (±4 kb for H2Aub1 and H3K27me3) around the TSS of the indicated group of PCGF target genes.

(G) Genomic snapshots of the ChIPseq profiles quantified in C at selected PCGF1/2 common and PCGF6 unique target gene loci performed in WT and the indicated KO mESCs.

(H) Percentage of deregulated PCGF6 targets genes co-occupied by RING1B (first two bars) or PCGF6 unique respect to RING1B (last two bars).

**Figure S8. PCGF3 and PCGF6 Recruit Modest RING1B Activity and Present Features of Active Transcription. Related to Figure 5.**

(A–D) Heatmap analysis representing the input subtracted normalized ChIP-seq intensities over ±8 kb centered at TSS of PCGF1 (A), PCGF2 (B), PCGF3 (C), PCGF6 (D) target loci stratified for RING1B co-occupancy in WT mESCs. H3K36me3 intensities are shown along the entire gene length (from TSS to TES).

(E) Boxplots of the input subtracted normalized intensity profiles of the indicated ChIPseq analyses performed in A-D. Signal enrichment is calculated over ±8 kb around the TSS of the indicated group of PCGF target genes.

(F) Western blot analysis with the indicated antibodies of protein lysates prepared from *Ring1A*$^{-/-}$;*Ring1B*$^{fl/fl}$;*Rosa26::CreERT2* (R1A KO-R1B FL) mESC treated with 4-OHT at the indicated time points. Vinculin served as loading control. *, unspecific signal.

(G, H) qPCR analysis at selected target regions of sequential RING1B ChIP (re-ChIP) experiments upon PCGF1 (G) or PCGF2 (H) ChIP in WT mESC. IgG served as control for ChIP assay. ChIP enrichments are

normalized to input. Data represent mean ± SEM.

**Figure S9. Loss of E2F6, MGA, or MAX Does Not Affect PCGF6 Expression. Related to Figure 6.**

(A) Venn diagram of PCGF6 (blue) and MGA (red) target genes.

(B) ChIP-qPCR validation of MGA and E2F6 binding at PCGF6 targets. IgG served as control for ChIP assay. ChIP enrichments are normalized to input. Data represent mean ± SEM.

(C) RT-qPCR analysis of *Mga* and *Pcgf6* mRNA levels in WT mESC expressing scramble (shCtrl) or specific shRNAs targeting *Mga*. *Gapdh* served as normalizing expression control. Fold change over shCtrl is shown. Data represent mean ± SEM.

(D) Western blot analysis with the indicated antibodies of protein lysates from cells described in C. Vinculin served as loading control.

(E) RT-qPCR analysis of *E2f6*, *Max* and *Pcgf6* mRNA levels in WT mESC expressing scramble (sh Ctrl) or shRNAs specifically targeting *E2f6* and *Max* in the indicated combinations. *Gapdh* served as normalizing expression control. Fold change over sh Ctrl is shown. Data represent mean ± SEM.

(F) Western blot analysis with the indicated antibodies of protein lysates from WT and *Mga∆HLH* mESC mutant expressing scramble (sh Ctrl) or shRNAs targeting specifically *E2f6* and *Max*. Vinculin served as loading control.

(G) Heatmaps of input subtracted normalized intensity profiles of MYC in WT mESC and PCGF6 binding in WT or *Pcgf6* KO, a sh*Mga*, *Mga*∆HLH mutant, sh*E2f6*, and a sh*E2f6* in *Mga*∆HLH mutant mESCs around ±2.5 kb of the TSS of PCGF6 targets stratified by the presence of E2F, E-box, E2F and E-box and unrelated binding motifs predicted by FIMO.

(H) Boxplots of the input subtracted normalized intensity profiles of ChIP-seq analyses performed in (G) over ±500 bp respect to the TSS.

**Figure S10. USF1 Interacts with PCGF3, which is not Affected by USF1 Loss. Related to Figure 7.**

(A) Input subtracted normalized intensity profiles and heatmap of USF1 binding in WT mESCs around ±2.5 kb of the TSS of USF1-Pcgf3 common and USF1 unique target loci.

(B) Western blot analysis with the indicated antibodies of USF1 immuno-precipitations using WT mESC nuclear extracts. Mouse IgG served as unrelated antibody. Input is shown as loading control.

(C) RT-qPCR analysis of *Usf1* and *Usf2* mRNA levels in WT mESC expressing scramble (sh Ctrl) or shRNAs targeting specifically *Usf1* and *Usf2* in the indicated combinations. *Gapdh* served as normalizing expression control. Fold-change over sh Ctrl is shown. Data represent mean ± SEM.

(D) Western blot analysis with the indicated antibodies of protein lysates from cells described in (C). Vinculin served as loading control.

(E) RT-qPCR analysis of *Pcgf3* mRNA levels in WT mESC expressing scramble (sh Ctrl) or shRNAs specifically targeting *Usf1* and *Usf2* in the indicated combinations. *Gapdh* served as normalizing expression control. Fold change over sh Ctrl is shown. Data represent mean ± SEM.

(F) USF1 ChIP-qPCR analysis at selected PCGF3 target regions performed in WT and *Pcgf3/5* KO mESCs. IgG served as a negative control for ChIP. ChIP enrichments are normalized to input. Data represent mean ± SEM.

## Supplemental Table Legends

**Table S1. shRNA and sgDNA sequences. Related to Star Methods**

List of oligo sequences used for RNAi and CRISPR/cas9 manipulations of mESCs.

**Table S2. PCGF Enriched Genomic Regions. Related to Figure 1**

List of peaks called for the different PCGF ChIP in WT mESCs.

**Table S3. PCGF Target genes. Related to Figure 1**

List of target promoters bound by each PCGF protein in WT mESCs.

**Table S4. Gal4-PCGF fusion proteins MS results. Related to Figure 2**

Values of the LFQ ratios of the different PRC1.1-6 components obtained by MS/MS analyses in the Gal4-PCGF1-6 fusions immuno-purifications (anti-Gal4) from 293TRex cells.

**Table S5. Gene Expression in PCGF KO ESC. Related to Figure 4**

Expression values of all RefSeq genes in the different PCGF KO mESC lines.

**Table S6. Gene Ontology of Differentially Expressed Genes. Related to Figure 4**

List of significant GO terms enriched in differentially expressed genes in the different PCGF KO mESC lines.
